# Viruses and Human Milk: Transmission or Protection?

**DOI:** 10.1016/j.advnut.2023.08.007

**Published:** 2023-08-20

**Authors:** Rachele Francese, Chiara Peila, Manuela Donalisio, Cristina Lamberti, Simona Cirrincione, Nicoletta Colombi, Paola Tonetto, Laura Cavallarin, Enrico Bertino, Guido E. Moro, Alessandra Coscia, David Lembo

**Affiliations:** 1 Department of Clinical and Biological Sciences, Laboratory of Molecular Virology and Antiviral Research, University of Turin, Orbassano (TO), Italy; 2 Department of Public Health and Pediatrics, Neonatal Intensive Care Unit, University of Turin, Turin, Italy; 3 Institute of the Science of Food Production - National Research Council, Grugliasco, TO, Italy; 4 Biblioteca Federata di Medicina "Ferdinando Rossi", University of Turin, Turin, Italy; 5 Italian Association of Human Milk Banks (AIBLUD), Milan, Italy

**Keywords:** human milk, virus detection, antiviral activity, mother-to-child transmission, pasteurization

## Abstract

Human milk (HM) is considered the best source of nutrition for infant growth and health. This nourishment is unique and changes constantly during lactation to adapt to the physiological needs of the developing infant. It is also recognized as a potential route of transmission of some viral pathogens although the presence of a virus in HM rarely leads to a disease in an infant. This intriguing paradox can be explained by considering the intrinsic antiviral properties of HM. In this comprehensive and schematically presented review, we have described what viruses have been detected in HM so far and what their potential transmission risk through breastfeeding is. We have provided a description of all the antiviral compounds of HM, along with an analysis of their demonstrated and hypothesized mechanisms of action. Finally, we have also analyzed the impact of HM pasteurization and storage methods on the detection and transmission of viruses, and on the antiviral compounds of HM. We have highlighted that there is currently a deep knowledge on the potential transmission of viral pathogens through breastfeeding and on the antiviral properties of HM. The current evidence suggests that, in most cases, it is unnecessarily to deprive an infant of this high-quality nourishment and that the continuation of breastfeeding is in the best interest of the infant and the mother.

**Statement of Significance**

In this review, we provide a comprehensive overview of numerous aspects related to a wide range of viral pathogens and human milk, including viral detection in human milk and viral transmission through breastfeeding, the antiviral properties of human milk, and the impact of milk processing on the human milk value. Through well-structured and informative tables, the reader will be guided toward a quick and easy road to understand this subject.

## Introduction

**FIGURE 1:**
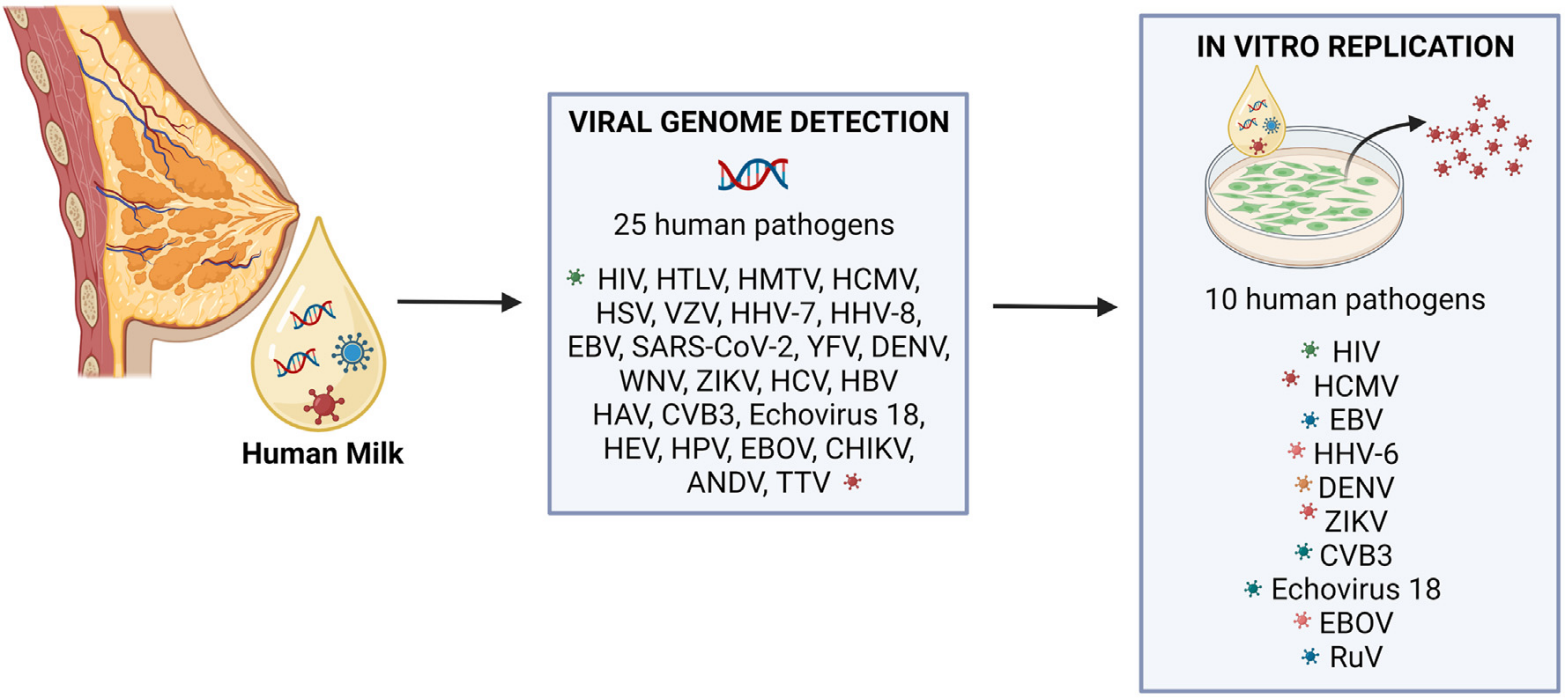
The detection of viruses in human milk.

**FIGURE 2:**
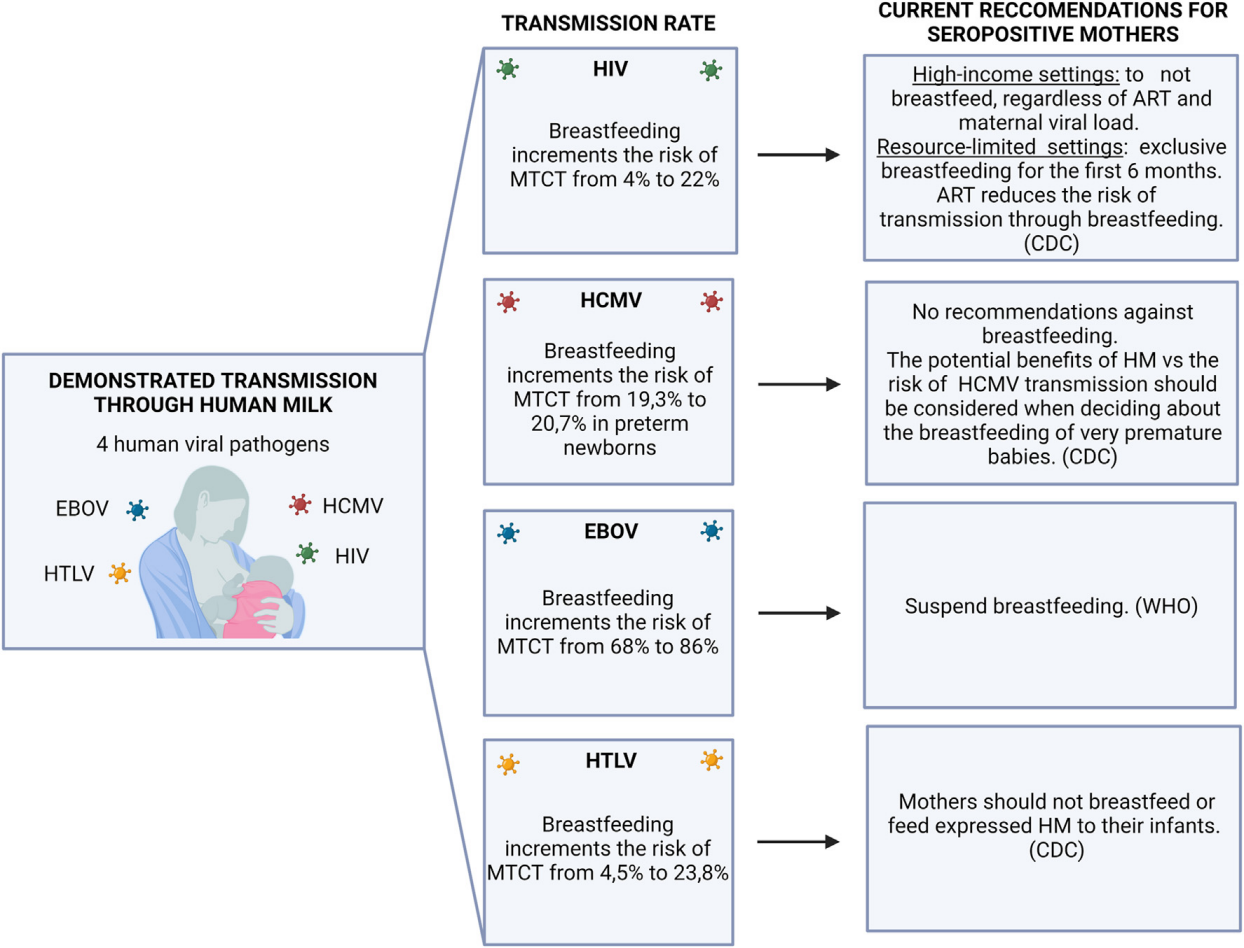
Viruses transmitted through human milk.

Human milk (HM) is considered the ideal nutrition for newborn children and infants: it is a dynamic species-specific biological fluid that varies to a great extent from one woman to another, and during lactation by adapting to the physiological needs of the infant [[1], [2], [3]]. The World Health Organization recommends exclusive breastfeeding until 6 mo of age as the best form of nutrition for the growth and development of infants [[4], [5], [6]]. These recommendations also consider the beneficial effects of breastfeeding on the short- and long-term health outcomes of the mother and infant [1,7]. HM plays the most critical role in the protection against infections for both term and preterm newborns [8,9]. However, an intriguing paradox has been described regarding the protective activity of HM against viral infections: several viral pathogens have been detected in HM (see Table 1) [[10], [11], [12], [13]], but the content of these pathogens rarely leads to an infection or disease in infants [[14], [15], [16]]. This evidence can be explained by considering the unique universe of bioactive components of HM: not only immunological factors, but also aspecific anti-infective components, which provide the first line of defense against pathogens, have been found in this biofluid [17]. However, this complex system is still partially unknown, probably due to the extremely variable nature of HM. To the best of our knowledge, no comprehensive review of the literature on this topic, including a comparison of the related results, is currently available. In this study, we describe which viruses have been detected in HM so far (Figure 1) and what the potential transmission risk of these viruses through breastfeeding is (Figure 2). We have provided a description of all the known antiviral compounds of HM, along with an analysis of their demonstrated or hypothesized mechanisms of action. Finally, because donor milk is considered the best choice when a mother’s own milk is unavailable, we have also analyzed the impact of pasteurization and storage methods on the detection and transmission of viruses, and on the antiviral compounds of HM.

**TABLE 1:** Viruses detected in HM

Virus family	Virus	Genome detection	Infectious particle detection	References
*Retroviridae*	HIV	+	+	[10,[18], [19], [20], [21], [22], [23], [24], [25], [26], [27], [28], [29], [30], [31]]
	HTLV-I/HTLV-II	+		[11,12,[32], [33], [34], [35], [36], [37], [38],^39^]
	HMTV	+		[40]
*Herpesviridae*	HCMV	+	+	[[41], [42], [43], [44], [45], [46], [47], [48], [49], [50], [51], [52], [53], [54], [55], [56], [57]]
	HSV-1/ HSV-2	+		[58,59]
	VZV	+		[42,58,60]
	HHV-6		+	[61]
	HHV-7	+		[62]
	HHV-8	+		[63,64]
	EBV	+	+	[[65], [66], [67], [68], [69], [70], [71]]
*Coronaviridae*	SARS-CoV-2	+		[72,73]
*Flaviviridae*	YFV	+		[74]
	DENV	+	+	[75,76]
	WNV	+		[77,78]
	ZIKV	+	+	[[79], [80], [81], [82], [83], [84]]
	HCV	+		[85,86]
*Hepadnaviridae*	HBV	+		[[88], [89], [90], [91], [92], [93], [94], [95], [96], [97], [98], [99], [100], [101], [102], [103], [104], [105], [106], [107], [108], [109], [110], [111], [112], [113], [114], [115], [116], [117], [118], [119], [120], [121], [122], [123], [124], [125], [126], [127], [128], [129], [130], [131], [132], [13], [133], [134], [135], [136], [137], [138], [139], [140], [141], [142], [14], [144], [145], [146], [147], [148], [149], [150], [151], [152], [153], [154], [155], [156], [157], [158], [159], [160], [161], [162], [163], [164], [165], [166], [167], [168], [169], [170], [171], [172], [17], [174], [175], [176], [177], [178], [179], [180], [181], [182], [183], [184], [185], [186], [187], [188], [189], [190], [191], [192], [193], [194], [195], [196], [197], [198], [199], [200], [201], [202], [203], [204], [205], [206], [207], [208], [209], [210], [211], [212], [213], [214], [215], [216], [217], [218], [219], [220], [221], [222], [223], [224], [225], [226], [227], [228], [229], [230], [231], [232], [233], [234], [235], [236], [237], [238], [239], [240], [241], [242], [243], [244], [245], [246], [247], [248], [249], [250], [251], [252], [253], [254], [255], [256], [257], [258], [259], [260], [261], [262], [263], [264], [265], [266], [267], [268], [269], [270], [271], [272], [273], [274], [275], [276], [277], [278], [279], [280], [281], [282], [283], [284], [285], [286], [304], [287], [288], [289], [290], [291], [292], [293], [294], [295], [296], [297], [298], [299], [91], [300], [301], [302], [303], [304], [305], [306], [307], [30], [309], [310], [311], [312], [313], [314], [315], [316], [317], [318], [319], [320], [321], [322], [323], [324], [325], [326], [327], [328], [329], [330], [331], [332], [333], [334], [335], [336], [337], [338], [339], [340], [341], [33], [343], [344], [345], [346], [347], [348], [349], [350], [351], [352], [353], [354], [355], [356], [357], [358], [359], [360], [361], [362], [363], [364], [365], [366], [367], [368], [369], [370], [371], [372], [373], [374], [291], [375], [376], [377], [378], [379], [380], [381], [382], [383], [360], [361], [384], [385], [386], [387], [388], [362], [363], [389], [390], [391], [392], [393], [394], [371], [395], [396], [397], [364], [398], [399], [400], [401], [402], [403], [404], [405], [406], [407], [408], [409], [410], [411], [412], [413], [414], [415], [416], [417], [418], [419], [420], [421], [422], [423], [424], [425], [426], [427], [429], [430], [431], [432], [433], [434], [435], [436], [437], [438], [439], [440], [441], [442], [443], [444], [370], [445], [446], [447], [448]]
*Picornaviridae*	HAV	+		[87]
	CVB3	+	+	[88]
	Echovirus 18	+	+	[89]
*Hepeviridae*	HEV	+		[90]
*Papillomaviridae*	HPV	+		[13,67,91]
*Filoviridae*	EBOV	+	+	[[92], [93], [94], [95]]
*Togaviridae*	CHIKV	+		[96]
*Hantaviridae*	ANDV	+		[97]
*Anelloviridae*	TTV	+		[98]
*Matonaviridae*	RuV		+	[99]
	Bacteriophages	+		[[100], [101], [102]]

ADNV, Andes virus; CHIKV, chikungunya virus; CVB3, coxsackievirus B3; DENV, dengue virus; EBOV, Ebola virus; EBV, Epstein–Barr virus; HAV, hepatitis A virus; HAVHHV-6, human herpes virus 6; HBV, hepatitis B virus; HCMV, human cytomegalovirus; HCV, hepatitis C virus; HEV, hepatitis E virus; HHV-7, human herpes virus 7; HHV-8, human herpes virus 8; HM, human milk; HMTV, human mammary tumor virus; HPV, human papilloma virus; HSV-1/HSV-2, herpes simplex virus types 1 and 2; HTLV-I/HTLV-II, human T-lymphotropic virus; RuV, Rubella virus SARS-CoV-2, severe acute respiratory syndrome coronavirus 2; TTV, TT virus; VZV, varicella-zoster virus; WNV, West Nile virus; YFV, Yellow fever virus; ZIKV, Zika virus.

Notes: References include English articles with full-text available. Clinical studies, observational studies, and in vitro studies were considered.

## Methods

This is a narrative review. The literature review was performed by conducting electronic searches in PUBMED (National Library of Medicine (United States), National Center for Biotechnology Information, available from https://pubmed.ncbi.nlm.nih.gov) and EMBASE (Elsevier Ltd., available from https://www.embase.com). The main strings used for the electronic search are reported in the Supplementary Materials.

### Viruses Detected in HM

Cell-associated viruses, or cell-free virions, can be detected in the HM of infected mothers by evaluating the presence of its components, such as the genome and antigens, or directly from the infectious viral particles. As shown in Table 1 and Figure 1, a long list of viruses has been detected in HM.

### *Retroviridae* family

Human immunodeficiency virus type 1 (HIV-1) is one of the most studied viruses transmitted by human breastfeeding, and in-depth knowledge of its milk reservoirs has been achieved. HIV-1 can be found in HM as both cell-free and cell-associated virions, and both have been associated with postnatal transmission [[18], [19], [20]]. The virus can be present in cells in 2 potential forms: as proviral DNA in latent, nonproductive, HIV-infected cells, and as an infectious particle in productive infected cells.

The virus can enter HM by transudation from the vascular compartment, and cell-associated or cell-free virions may continually traffic from blood to milk [18]. Furthermore, it has been widely evidenced that the virus can also replicate locally in mammary gland tissues, and the breast has been considered a retroviral reservoir that contributes to infectious inoculum in milk [21,22].

Detectable HIV-1 ribonucleic acid (RNA) in whey has been reported in most untreated lactating women. Elevated levels of HIV-1 RNA in breast milk have generally been associated with higher maternal plasma virus loads, lower maternal CD4+ T-cell counts, the detection of viral DNA in maternal genital secretions, and breast inflammation [23].

The cell-free viral loads in HM, measured by means of quantitative reverse transcriptase-polymerase chain reaction (RT-PCR), are usually much lower than in the plasma of mothers [24,25]. Lewis et al. [26] reported that, if no antiretroviral drugs are used, the plasma of most HIV-1 infected people contains 10^4^–10^6^ copies of HIV-1/mL, whereas tested HM samples were found to contain 240–8100 copies/mL. Fluctuations over time in the RNA virus level in breast milk have been detected. Rousseau et al. [23] found that HIV-1 levels were higher in colostrum than those in mature breast milk. Several studies have reported that the prevalence of cell-free HIV-1 is generally higher in mature milk than that in earlier milk samples [20,26].

HM contains 10^4^–10^6^ cells/mL, including cellular types that are susceptible to HIV-1 infection: productively infected lymphocytes, macrophages, and luminal epithelial cells have been detected in both early and mature milk samples [[26], [27], [28]]. It has been estimated that the ingestion of 100 mL of colostrum or 800 mL of mature milk from an infected woman may expose a newborn to an average of 25,000 infected cells [20]. HIV replication occurs in activated CD4+ cells, despite the presence of a maternal antiretroviral treatment [20,29]. An important cellular reservoir in breast milk, which even persists during an efficient antiretroviral therapy, is composed of latently infected CD4+ T lymphocytes, which harbor HIV-1 DNA [30,31]. Interestingly, certain findings have evidenced that multiple independent lineages of HIV-1 persist in HM and plasma [28].

Human T lymphotrophic virus type 1 (HTLV-1) is a highly oncogenic retrovirus. Unlike HIV, cell-free HTLV-1 virions have not been detected in HM. The first studies showed the expression of viral antigens in infected mononuclear cells from seropositive mothers [[32], [33], [34], [35]]. Subsequently, the presence of both HTLV-1 and HTLV-2 genomes was detected in HM cell pellets by means of PCR [[36], [37], [38]]. Regarding what milk cells can transmit the virus, macrophages are considered the most frequent carriers in colostrum, and 0.1%–1% cells in early milk have been estimated to be positive to HTLV-1 capsid protein expression [22,103]. Moreover, a prominent role may also be attributed to lymphocytes and epithelial cells in mature milk; however, the infection of lymphocytes in HM has still to be determined.

In a similar way as for HIV, the glandular epithelium of the breast can act as a viral reservoir of in vivo productively infected cells: infected alveolar luminal epithelial cells and transformed T cells have been detected in breasts, and basal mammary epithelial cells have been found to be susceptible to infection [103,39].

The *Retroviridae* family also includes the human mammary tumor virus (HMTV), whose genome has been detected in breast cancers. Nartey et al. [40] detected viral env sequences, by means of PCR, in milk samples from lactating women with an increased risk of breast cancer.

### *Herpesviridae* family

Human cytomegalovirus (HCMV), a beta-herpesvirus, was first isolated from HM in human fibroblast tissue cultures in the early 1970s [41]. These initial studies evidenced a prevalence of virolactia, that is, the presence of an infectious virus in unseparated milk specimens. An increased percentage of viral shedding was then demonstrated among HCMV-seropositive mothers, which depended on the time of detection and the used method [[42], [43], [44], [45], [46], [47], [48]]. Today, HCMV secretion into HM is monitored, using the PCR technology, by evaluating the rate of HCMV DNAlactia, that is, the presence of viral DNA in samples. It is now well established that nearly all HCMV-seropositive women will reactivate and shed HCMV during lactation. A predominant role of cell-free virus transmission via HM has been evidenced by studying the kinetics of maternal HCMV reactivation during lactation by PCR and viral cultures; indeed, maternal risk factors for HCMV transmission to breastfed preterm infants were early onset of viral DNA in milk whey (median 3.5 d postpartum in transmitters) and infectious virus in milk whey (median 10 d postpartum in transmitters) [49].

Studies performed on milk samples from seropositive breastfeeding mothers of preterm infants evidenced that HCMV reactivation, based on virolactia and viral DNAlactia, was a self-limiting process that exhibits unimodal kinetics characterized by a longitudinal increase, a peak level, and a decrease during lactation [45,46,48,50,51]. This process of reactivation occurs in all lactating women, but the peak level of viral DNA copy numbers and viral load, as well as the time scale of viral shedding in milk show a great interindividual heterogeneity [44,47,[51], [52], [53]]. The viral shedding may start during the first week postpartum in colostrum as early as on day 3 [52]. The highest viral load is reached at ∼4–8 wk and the HCMV secretion into milk usually ends from 9 to 12 wk after delivery. However, several studies detected the HCMV shedding until 9 mo after birth [44,51]. In this contest, a longitudinal cell-free and cell-associated HCMV monitoring was performed detecting virolactia in both compartments during early lactation. This study confirmed that the active viral replication appeared to be a self-limited process during lactation: virolactia ended on day 44 in milk cells and on day 58 in the whey [50]. It should be pointed out that there is a great consensus about the fact that HCMV reactivation may occur locally in the breast during lactation [45,46,49]. Regarding the infected cellular compartment, HCMV DNA as well as viral pp67 mRNA has been detected in CD14-positive breast milk cells [50]. Interestingly, a metagenomic sequencing conducted to characterize the DNA virome of HM samples evidenced that 60.1% of the virome was represented by *Herpesviridae,* and that HCMV was the dominant one. Moreover, different HCMV isolates have been demonstrated by means of phylogenetic analysis [[54], [55], [56], [57]].

The presence of beta-herpesviruses human herpesvirus 6 and 7 (HHV-6 and HHV-7) has rarely been described in HM. Their DNA has been detected, by means of PCR, in milk cells, and the activated virus might be present in the milk whey of HIV-infected mothers [61,62].

Herpes simplex virus types 1 and 2 (HSV-1, HSV-2) postnatal infections are rare and are mainly related to maternal HSV-positive breast lesions [58,59]. HSV DNA has been localized in mononuclear cells and, albeit to a lesser extent, in epithelial cells. Furthermore, a single study has reported the detection of varicella-zoster virus (VZV) DNA, by means of PCR, in the HM of a mother affected by herpes zoster, although it was not possible to exclude contamination from skin lesions [58,104,60].

Regarding gamma-herpesviruses, the presence of the Epstein–Barr virus (EBV) in HM has been demonstrated by evaluating both the viral DNA and infectious particles [[65], [66], [67], [68], [69], [70]]. The shedding of EBV in HM has been associated with EBV DNA in maternal plasma [71]. Several studies have examined the association of EBV in milk with HIV-1 shedding and mastitis. The presence of EBV in milk may impact HIV shedding, but discordant data have emerged about the association of coinfections with clinical or subclinical mastitis [66,69]. Daud et al. [68] demonstrated, by exposing peripheral blood mononuclear cells to breast milk, that the virus was infectious in HM.

The detection of HHV-8, also known as Kaposi sarcoma-associated herpesvirus, in HM has been documented in 2 papers [63,64]. However, the very low detected DNA load suggests that contact with HM is not a likely source of transmission to infants.

### SARS-CoV-2

The detection of severe acute respiratory syndrome coronavirus 2 (SARS-CoV-2) RNA in breastmilk is the most commonly used way of establishing the potential transmission of the virus via breastmilk, but its significance, related to infectivity, is not yet clearly understood. Chambers et al. [72] evaluated the replication competency of SARS-CoV-2 in breastmilk using viral culture methods. Of all the tested samples, including one that was positive to RT-PCR testing, none showed evidence of a cytopathic effect in the cell culture, thus suggesting that RNA may not represent a replication-competent virus in breastmilk. In a recent paper [73], HM from 110 lactating women was analyzed, by means of RT-PCR (285 samples), and by viral culture (160 samples). Those containing SARS-CoV-2 viral RNA (vRNA) were examined for the presence of subgenomic RNA (sgRNA), a putative marker of infectivity. SARS-CoV-2 vRNA was detected in the milk of 7 (6%) women with either a confirmed infection or a symptomatic illness, including 6 out of 65 (9%) women who had resulted positive to a SARS-CoV-2 diagnostic test. No infectious virus was detected in any of the cultures and none of them had detectable sgRNA. This study is the first ever to have used viral cultures to examine a large number of breast milk specimens [105].

### *Flaviviridae* family

Among the arthropod-transmitted flaviviruses, the presence of the Yellow Fever virus (YFV) and the West Nile virus (WNV) genome has rarely been detected in HM [[74], [77], [78]]. The viral genome and infectious viral particles have both been detected regarding the dengue virus (DENV). Breast milk DENV-1 loads have been found to be in the same range as the ones found in the motherʼs blood and breast milk culture, thus confirming the ability of the virus to replicate in cells [75,76].

The literature is more extensive regarding the shedding of the Zika virus (ZIKV) in HM. It has clearly been demonstrated, by means of RT-qPCR, that HM is positive to ZIKV, with a high viral load, and the virus has been detected in both colostrum and mature milk [[79], [80], [81], [82]]. Both cell-free and cell-associated viruses are excreted into this biofluid [83]. Evidence of a cytopathic effect in infected milk cells has confirmed the presence of infective viral particles in HM [81,82,84].

### Hepatitis viruses

The presence of the hepatitis B virus (HBV) in HM has clearly been demonstrated by detecting viral proteins [[106], [107], [108], [109]]. HBsAg and HBeAg titers in HM samples have been found to be lower than those in the sera, although positively correlated [107,110]. Since the advent of molecular biology techniques, viral DNA has been detected in both colostral whey and colostral cells. However, a high level of viral DNA in HM does not seem to rule out breastfeeding for fully vaccinated children [111]. Breastfeeding from hepatitis C virus (HCV) mothers is considered safe for infants, because only a few somewhat older papers reported vRNA in colostral samples, albeit in significantly low levels [85,86].

Finally, the detection of the hepatitis A virus (HAV) and hepatitis E virus (HEV) genome in HM from women with acute viral infections has only been documented once [87,90].

### Other known or emerging viruses

Other viruses have been detected in HM, but the debate is still open for many of them. Among the Picornaviridae members, coxsackievirus B3 (CVB3) and echovirus 18 have been evidenced in mothers’ milk by detecting both RNA and the infectious virus [88,89].

Human papillomaviruses (HPV) have been searched for in HM by means of PCR, hybridization, and sequencing. A direct role of HPV in breast carcinogenesis is unlikely because the milk positivity for cutaneous and mucosal HPV types is generally low [67,112,113]. The attenuated live Rubella virus (RuV) has been detected in breast milk from mothers after postpartum immunization, but there are no studies that demonstrate the shedding of the wild-type virus in milk [99].

Among the emerging viruses, the presence of the Ebola virus (EBOV) in HM has been documented by detecting the vRNA and infectious particles in cell cultures [[92], [93], [94]]. The viral load was found to be lower in HM than in blood when they were tested concomitantly, but the virus was found to persist in milk for at least 26 d after the onset of the symptoms [95].

Among the *Hantaviridae* members, the Andes virus (ANDV) is the only one that is transmitted between humans through close contact. Ferres et al. detected the ANDV genome and proteins in HM cells [97]. One paper has reported the detection of the chikungunya virus (CHIKV), a mosquito-borne virus, in HM, by means of RT-PCR, thus raising clinical questions [96].

The most abundant eukaryotic viruses in the human virome belong to the *Anelloviridae* family, and they have been detected in a variety of biological fluids. Among these viruses, the Torque teno virus (TTV) has been found in breast milk [98]. Most of abovementioned viruses are human pathogens that can be detected in HM during an acute or latent infection of a mother. However, the most ubiquitous and abundant viruses present in HM are bacteriophages, which that infect the bacteria that compose the HM microbioma [100]. According to Pannaraj, who conducted a sequencing study, the dominant phage families in HM are Myoviridae, Siphoviridae, and Podoviridae, but the virome pattern of HM is variable and depends on the mode of delivery, the birth weight, and the lactational stage [101,102].

### Virus Transmission through Breastfeeding

In most cases, the presence of an infectious disease in a mother does not rule out breastfeeding. However, there are a few specific viruses that require special consideration (Figure 2). The data in Table 2 [14,15,50,69,[114], [115], [116], [117], [118], [119], [120], [121], [122], [123], [124], [125], [126], [127], [128], [129], [130], [131], [132], [133], [134], [135], [136], [137], [138], [139], [140], [141], [142], [143], [144], [145], [146], [147], [148], [149], [150], [151], [152], [153], [154], [155], [156], [157], [158], [159], [160], [161], [162], [163], [164], [165], [166], [167], [168], [169], [170], [171], [172], [173], [174], [175], [176], [177], [178], [179], [180], [181], [182], [183], [184], [185], [186], [187], [188], [189], [190], [191], [192], [193], [194], [195], [196], [197], [198], [199], [200], [201], [202], [203], [204], [205], [206], [207], [208], [209], [210], [211], [212], [213], [214], [215], [216], [217], [218], [219], [220], [221], [222], [223], [224], [225], [226], [227], [228], [229], [230], [231], [232], [233], [234], [235], [236], [237], [238], [239], [240], [241], [242], [243], [244], [245], [246], [247], [248], [249], [250], [251], [252], [253], [254], [255], [256], [257], [258], [259], [260], [261], [262], [263], [264], [265], [266], [267], [268], [269], [270]] indicate the viruses that could be transmitted through breastfeeding, and the current recommendations in force.

**TABLE 2:** Viruses transmitted through breastfeeding

Virus family	Virus	Demonstrated transmission through breastfeeding	Uncertain/ low risk of transmission through breastfeeding	Current recommendations	References
*Retroviridae*	HIV	X		High-income settings with access to clean water and affordable replacement feeding (infant formula): to not breastfeed, regardless of ART and maternal viral load. Resource-limited settings: to breastfeed exclusively for the first 6 mo of life and continue breastfeeding for at least 12 mo, with the addition of complementary food. These mothers should be given ART to reduce risk of transmission through breastfeeding. (CDC)	[103,69,[114], [115], [116], [117], [118], [119], [120], [121], [122], [123], [124], [125], [126], [127], [128], [129], [130], [131], [132], [133], [134], [135], [136], [137], [138], [139], [140], [141], [142], [143], [144], [145], [146], [147], [148], [149], [150], [151], [152], [153], [154], [155], [156], [157], [158], [159], [160], [161], [162], [163], [164], [165], [166], [167], [168], [169],^271^,^272^]
	HTLV	X		Mothers should NOT breastfeed or only feed expressed breast milk to their infants. (CDC)	[11,12,33,37,103,[170], [171], [172], [173], [174], [175], [176], [177], [178], [179], [180], [181], [182], [183], [184], [185], [186], [187], [188], [189], [190],^273^,^274^,^275^,^405^]
*Herpesviridae*	HCMV	X		There are no recommendations against breastfeeding by mothers who are HCMV-seropositive. However, infants born at a gestational age of <30 wk and <1500 g who acquire HCMV from breast milk may be at risk of developing a late-onset sepsis-like syndrome. The potential benefits of human milk versus risk of HCMV transmission should be considered when deciding about the breastfeeding of very premature babies by mothers known to be HCMV-seropositive. (CDC)	[14,15,[43], [44], [45],^49^,^50^,^52^,[191], [192], [193], [194], [195], [196], [197], [198], [199], [200], [201], [202], [203], [204], [205], [206], [207], [208], [209], [210], [211], [212], [213], [214], [215], [216], [217], [218], [219], [220], [221],^276^,^277^,[278], [279], [280], [281],^282^,^283^]
	HSV-1/HSV-2	X (Through breast lesions)		Mothers with active lesions on the breast should temporarily stop breastfeeding from the affected breast (CDC)	[[222], [223], [224],^284^,^285^]
	VZV		X	Continue to breastfeed. In the case of a potential risk of perinatal VZV, expressed breast milk can be given to a newborn, if there are no skin lesions on the breasts during the period of the mother's infectivity. (CDC)	[286]
*Coronaviridae*	SARS-CoV-2		X	Continue to breastfeed (CDC, WHO, UNICEF, AMB, EMBA, UENPS, SIN)	[73,[225], [226], [227], [228], [229], [230],[287], [288], [289],^290^,^291^]
*Flaviviridae*	YFV		X	Vaccination is recommended, if vaccination is indicated as suitable for a breastfeeding woman, and travel cannot be avoided or postponed (WHO)	[231,232,292,293]
	DENGUE		X	Continue to breastfeed (CDC)	[75,76,233,234]
	WNV		X	Continue to breastfeed (CDC)	[235,294]
	ZIKV		X	Continue to breastfeed (WHO)	[[79], [80], [81], [82],[236], [237], [238], [239], [240], [241], [242], [243], [244], [245], [246], [247],^295^,^296^]
	HCV	X if the nipples and/or surrounding areola are cracked and bleeding		There is no documented evidence that breastfeeding spreads HCV. However, HCV is spread by infected blood. Therefore, if the HCV-positive mother’s nipples and/or surrounding areola are cracked and bleeding, she should stop nursing temporarily. (CDC)	[85,86,[248], [249], [250], [251], [252], [253], [254], [255], [256], [257],^297^]
*Hepadnaviridae*	HBV		X	All infants born to HBV-infected mothers should receive hepatitis B immune globulin and the first dose of hepatitis B vaccine within 12 h of birth. There is no need to delay breastfeeding until the infant is fully immunized. (CDC)	[107,[258], [259], [260], [261], [262], [263], [264],^298^]
*Hepeviridae*	HEV		X	Continue to breastfeed. Women with a symptomatic HEV infection, especially with high viral loads of viremia, should not breastfeed. (Chibber, 2004)	[299]
*Picornavirus*	HAV		X	Continue to breastfeed	[87,265]
	CBV3		X	Continue to breastfeed	[88]
*Papillomaviridae*	HPV		X	Continue to breastfeed (LactMed® + Biblio)	[112,266,91]
*Filoviridae*	EBOLA	X		Suspend breastfeeding (WHO)	[92,93,95,[267], [268], [269]]
*Hantaviridae*	ANDV		X	Continue to breastfeed	[97]
*Matonaviridae*	RuV		X	Continue to breastfeed (LactMed® + Biblio)	[99,270,300,301]

ADNV, Andes virus; CVB3, coxsackievirus B3; DENV, dengue virus; EBOV, Ebola virus; HAV, hepatitis A virus; HBV, hepatitis B virus; HCMV, human cytomegalovirus;

HCV, hepatitis C virus; HEV, hepatitis E virus; HPV, human papilloma virus; HSV-1/HSV-2, herpes simplex virus types 1 and 2; HTLV-I/HTLV-II, human T-lymphotropic virus; RuV, Rubella virus; SARS-CoV-2, severe acute respiratory syndrome coronavirus 2; TTV, TT virus; VZV, varicella-zoster virus; WNV, West Nile virus; YFV, Yellow fever virus; ZIKV, Zika virus.

Notes: references include English articles with full-text available. Clinical studies, observational studies and in vitro studies were considered.

### *Retroviridae* family

#### HIV

Mother-to-child transmission (MTCT) of HIV can occur during pregnancy and during delivery, but also in the postpartum period through breastfeeding. In 2015, ∼150,000 new HIV infections and 110,000 HIV-related deaths occurred globally among children <15 y of age, with MTCT being the leading cause of new HIV infections. Breastfeeding by an HIV-1 positive mother increases the transmission risk from 4% to 22%, in addition to risk of prenatal and perinatal transmission.

In resource-poor situations, where the complete lack of HM can increase morbidity and mortality, there are other potential interventions that can be adopted to limit HIV-1 MTCT. The avoidance of breastfeeding by HIV-positive mothers should continue in countries where an alternative source of nutrition can easily be provided [271,272].

HIV-2 causes a clinical disease that is similar to HIV-1 infection, but which has a significantly slower progression to immune suppression. HIV-2 transmission through HM is less common than HIV-1 transmission, but risk of and possible factors contributing to transmission have not yet been fully quantified. Unless additional data become available concerning HIV-2 and HM, it is appropriate to follow the current guidelines (reported in Table 2) for breastfeeding related to HIV-1 infection [271,272].

#### HTLV

HTLV-I causes adult T-cell leukemia/lymphoma and HTLV-I associated myelopathy or tropical spastic paraparesis related to various other chronic conditions. HTLV-II causes at least 2 forms of chronic ataxia. Transmission occurs through sexual contact, from blood and blood products, and from HM. HTLV-I and -II transmission is more frequent in breastfed infants than in formula-fed ones. A longer period of breastfeeding is correlated with a greater risk of HTLV-I transmission to the infant. The complete avoidance of breastfeeding is an effective intervention to prevent MTCT (see the current recommendations in Table 2). Given the nature of HTLV-I and II (that is, early infection, late onset, progressive disease, and no available therapy), it is appropriate to emphasize prevention [[273], [274], [292]].

### *Herpesviridae* family

#### HCMV

HCMV is the most common cause of congenital infections. However, infection through HM rarely results in a significant disease in full-term infants. The acquisition of transplacental maternal anti-HCMV antibodies protects the full-term infants of HCMV-positive mothers. A primary HCMV infection rarely occurs in the mother during delivery and lactation, but there is an increasing risk of illness in the infant due to a lack of anti-HCMV antibodies. Susceptible infants (that is, premature infants, infants from HCMV-seronegative mothers, and immunodeficient infants) may be at a higher risk of developing a symptomatic postnatal HCMV disease. Short-term consequences of acquired HCMV infection in preterm infants include fever, pneumonitis, thrombocytopenia, lymphocytosis, hepatosplenomegaly, hepatitis, encephalitis, and an acute sepsis-like illness. Furthermore, infants born at a gestational age of <30 wk and of <1500 g who acquire HCMV from HM may be at risk of developing a late-onset sepsis-like syndrome and may have long-term pulmonary and neurodevelopmental morbidity, especially those with symptomatic infection [302]. Two meta-analyses have examined the rate of breast milk-acquired HCMV infections in preterm or very low birth weight infants (VLBWi) with HCMV-seropositive mothers. The obtained results suggest that there is a higher HCMV infection rate in VLBWi or premature infants fed fresh HM than in other feeding groups [275,276].

Of note, risk of HCMV infection, acquired by exposure to raw maternal milk, was most prominent in extremely premature newborns, born at ≤ 25 wk’ gestation, regardless of maternally acquired neutralizing antibody levels. Indeed, risk of acquired viral infection for infants born at 23–24 wk was evaluated 8 times higher compared with risk of those born at 29–30 wk [277]. The postnatal acquisition of HCMV in VLBW premature infants may occur from different routes that are not mutually exclusive. In detail, possible routes of entry include transmucosal in the oropharynx and/or nasopharynx, transjejunal via intestinal epithelial cells, Peyer’s patches, or M-cells and transolfactory. Among these, the transolfactory route has not been validated in human infants but identified in murine models [303].

Summarizing, HCMV-seropositive mothers can breastfeed full-term infants safely. However, the potential benefits of HM versus risk of HCMV transmission should be considered carefully when deciding whether to breastfeed very premature babies born from mothers known to be HCMV-seropositive.

#### HSV

HSV-1 and HSV-2 cause severe perinatal infections and, albeit less frequently, prenatal and postnatal infections. Certain case reports have demonstrated the presence of HSV infections in infants related to maternal HSV breast lesions and direct inoculation of the virus from primary gingivostomatitis during breastfeeding. Mothers with active lesions on the breast should therefore temporarily stop breastfeeding from the affected breast [284,285].

Breastfeeding or the use of expressed HM in the absence of breast lesions in a mother with other signs of active HSV infection is considered appropriate, albeit with careful contact precautions.

#### VZV

VZV infection causes chickenpox, as the primary infection, and zoster or shingles as a recurrent or reactivating infection. Only one case of suspected transmission of VZV through breastfeeding has been reported, although there was not adequate proof to exclude other more common modes of transmission. If a mother develops varicella, breastfed and formula-fed infants are equally at risk from close contact with her. As there is a lack of evidence on this topic, expressed HM can be given to a newborn in the case of potential risk of perinatal VZV, if there are no skin lesions on the mother’s breasts [286].

### SARS-CoV-2

One of the main concerns during the current pandemic has been connected to the safety of both asymptomatic and paucisymptomatic COVID19+ breastfeeding women. To date, all the reports suggest that the vertical transmission of SARS-CoV-2 is unlikely [[287], [288], [289], [304], [305], [306]]. However, the possible transmission of the virus, via breastfeeding, is still under debate. In a systematic review [290], 12 out of 183 women from 48 studies were found to be positive to the SARS-CoV-2 genome in their breastmilk, and 6 infants from these 12 mothers tested positive (50%) for SARS-CoV-2, although only 1 required respiratory support.

### *Flaviviridae* family

#### YFV

YFV and its protective vaccine can be transmitted through breastmilk, although the frequency of transmission is still unclear. YFV vaccine-associated neurologic disease is rare, but potentially severe adverse effects have been observed following immunization. The clinical manifestations of YFV infection ranges from asymptomatic to severe, and they include jaundice and hemorrhaging. The administration of the YFV vaccine to breastfeeding women should be avoided, except in situations where exposure to YFV cannot be avoided or postponed [292,293].

#### DENV

DENV causes dengue fever, dengue haemorrhagic fever, and dengue shock syndrome in infants of <1 y of age, but rarely in neonates below 3 mo of age. There is no evidence of the transmission of DENV through HM, or of a more severe form of the disease in breastfed infants than in formula-fed infants. No person-to-person transmission of DENV without a mosquito vector has been documented. Therefore, a mother with dengue disease should continue breastfeeding as long as they are able to [75].

#### WNV

WNV infection leads to ∼1 case of severe neurologic disease for every 20 cases of nonspecific febrile illness and every 150–300 cases of seroconversion. Children and infants of less than 1 y of age with an infection and clinical illness have rarely been reported. Transmission mainly occurs through mosquito bites, and only 1 case of possible WNV transmission through breastfeeding has been reported. Although this may constitute a particular case, the absence of illness in that infant, the transient nature of maternal viremia with WNV, and the rarity of such a transmission event all suggest that there is no reason for a mother to avoid breastfeeding when she is infected with WNV [294].

#### ZIKV

ZIKV is generally transmitted by daytime active *Aedes* mosquitoes, sexual contact, blood transfusions, and vertical transmission. In most cases, ZIKV infection is asymptomatic. Even though ZIKV has been detected in some HM samples, the data are not sufficient to conclude that ZIKV is transmitted via breastfeeding. More evidence is needed to distinguish breastfeeding transmission from other perinatal transmission routes [295].

### Hepatitis viruses

#### HCV

The natural course of pediatric HCV infection is characterized by a high rate of spontaneous clearance, an asymptomatic clinical course, and normal or mild histologic changes. Transmission occurs through blood and blood products. However, the transmission of HCV infection through breast milk has not yet been proved, and the transmission rates seem similar in formula-fed and breastfed infants. Additional controlled trials are needed to delineate the importance of the different factors that contribute to or limit MTCT. The CDC guidelines do not currently consider maternal HCV infection a contraindication to breastfeeding, although they suggest that cracked or bleeding nipples may increase risk of transmission [297].

#### HBV

Chronic HBV infection develops in 90% of the infants who are infected before or during birth. Transmission is primarily through blood or body fluids. The transmission of HBV may occur through HM, but there is no difference in the seroconversion rates of formula-fed and breastfed infants. An appropriate administration of hepatitis B immunoglobulin and HBV vaccine at birth for infants born to hepatitis B surface antigen-positive mothers prevents transmission in more than 95% of the cases, regardless of the mode of feeding [298].

#### HEV

HEV infection causes self-limiting acute hepatitis. The transmission of HEV occurs primarily through the fecal–oral route. Vertically transmitted HEV infection, via the intrauterine and perinatal routes, is known to cause acute hepatitis in newborn babies. Only 1 study present in the literature has demonstrated the presence of HEV in colostrum, but the authors concluded that there are no data to support withholding breast milk from infants born to HEV-infected mothers [299].

#### HAV

The infection in infants is usually mild, and there is no evidence of chronic HAV infection in infants. An infant has usually been exposed to HAV before the mother is diagnosed. The transmission of HAV through breast milk has only been implicated in 1 case report [87]. The infant of a mother with recently diagnosed HAV infection should receive immunoglobulin and an HAV vaccine.

### Other known or emerging viruses

#### CVB3

CVB3 can cause severe neonatal disease, and it has a high mortality rate. Clinical manifestations of the severe forms are generally nonspecific and sepsis-like. The infection often progresses into organ-localized syndromes, such as acute hepatitis and myocarditis. The transmission of CVB3 from mothers to neonates is relatively common and it can occur in the intrauterine, peripartum, and postpartum periods. Only in 1 case report was CVB3 found in the mother’s milk of 2 severely infected neonates [88].

#### HPV

HPV infections are common and are associated with a wide spectrum of benign mucosal and cutaneous lesions, cancer precursors, and cancer. The MTCT of HPV is known to occur during labor, delivery, and during breastfeeding. Although HPV infection can occur through HM, the likelihood of this happening is very low. Moreover, maternal vaccination with the HPV vaccine does not rule out breastfeeding [91].

#### EBOV

The mortality rate of EBOV infection is high in all age groups, but it is particularly high in fetuses and neonates. Although it is not contagious until the symptoms appear, the transmission of EBOV occurs via contact with the body fluids, including, but not limited to, urine, semen, saliva, sweat, and breast milk, of people who are infected. Transmission is likely to occur in the utero, because samples from amniotic fluid, placentas, and fetuses have all tested positive to EBOV, with the possibility of transmission also during delivery and breastfeeding [93].

#### ANDV

The main route of infection in humans is through the inhalation of aerosolized viral particles present in contaminated rodent excreta, but the virus can also be transmitted from person to person. One case report proved the presence of ANDV in HM. A newborn infant developed the main clinical manifestation of the presence of ANDV, the hantavirus cardiopulmonary syndrome, and subsequently died. The conclusion of this report was that breastfeeding could be considered as an additional transmission mechanism [97].

#### RuV

One case of neonatal rubella has been reported after postpartum infection with a wild-type virus in a breastfed infant. However, the infection was not proved to result from breast milk transmission, and other routes of infection were considered to be more likely [300]. In 1 small study on the transmission of the RuV vaccine after maternal immunization, an infectious vaccine virus was isolated from the nasopharynx and throat of breastfed infants with transient seroconversion, but not from the nonbreastfed controls. None of the infants infected with the vaccine strain developed a clinical disease [301].

### HM Antiviral Activity

**FIGURE 3:**
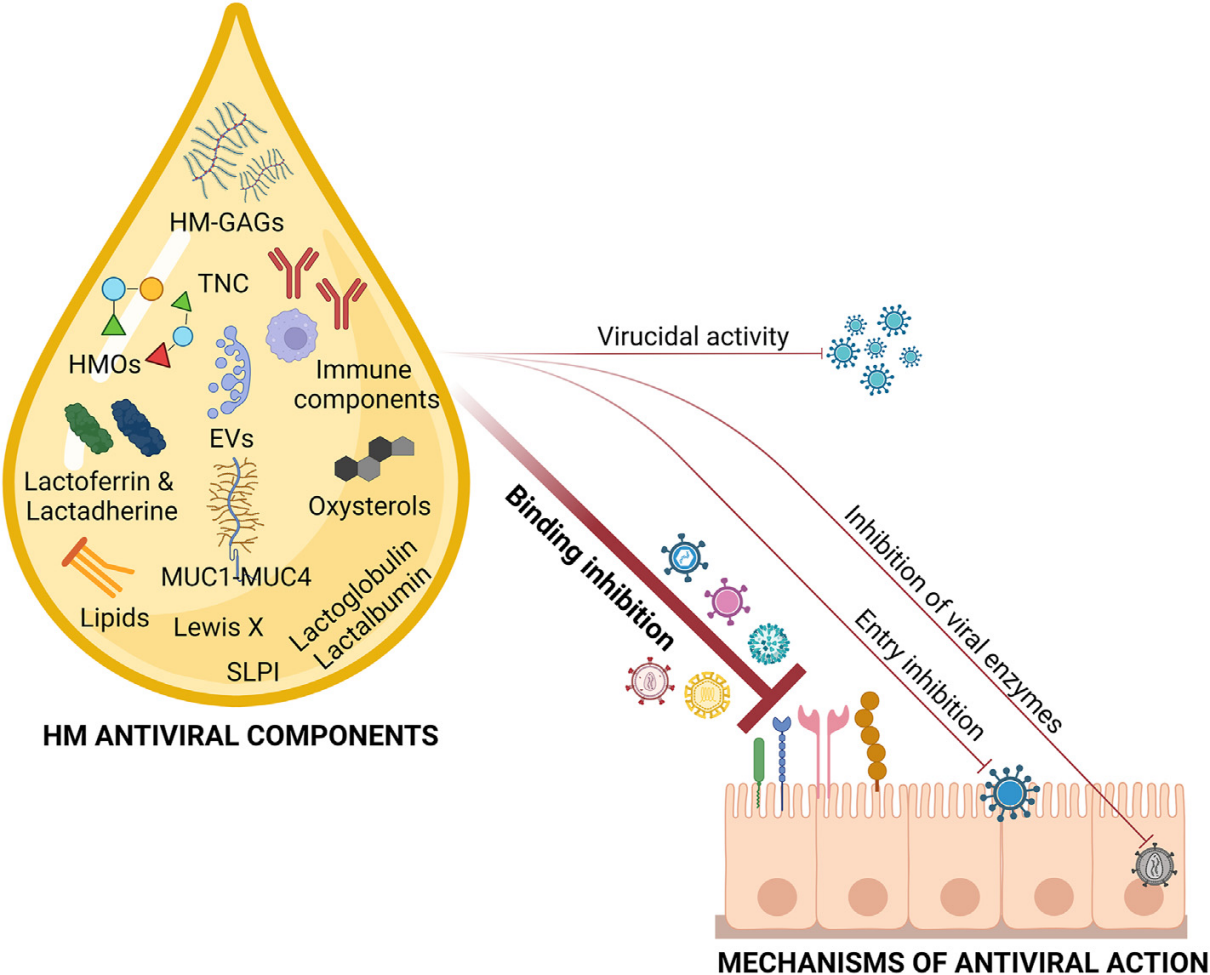
Mechanism of action of the antiviral components of human milk.

A multitude of antiviral components mediates the protective activity of HM and others will likely be discovered in the future. Table 3 [[307], [308], [309], [310], [311], [312], [313], [314], [315], [316], [317], [318], [319], [320], [321], [322], [323], [324], [325], [326], [327], [328], [329], [330], [331], [332], [333], [334], [335], [336], [337], [338], [339], [340], [341], [342], [343], [344], [345], [346], [347], [348], [349], [350], [351], [352], [353], [354], [355], [356], [357], [358], [359]] and Figure 3 summarize the major antiviral compounds that have been detected in HM so far, and their demonstrated or hypothesized mechanisms of action.

**TABLE 3:** Antiviral compounds in HM

HM Antiviral compound	In vitro activity	In vivo activity	Mechanism of action	References
Oligosaccharides (HMOs)	Norovirus, HRoV, FluV, HIV, Calicivirus	FluV, HRoV, Norovirus, HIV	Decoy receptor; binding inhibition;	[[307], [308], [309], [310], [311], [312], [313], [314], [315], [316], [317], [318], [319], [320], [321], [322], [323],^360^]
Glycosaminoglycans (HM-GAGs)	HIV, RSV, HCMV, ZIKV, USUV	/	Binding inhibition	[[324], [325], [326], [327],^361^]
Lactoferrin (Lf)	HRoV, HIV, HCMV, RSV, HSV-1, HSV-2, HBV, HCV, HGV, Poliovirus, ADV, EV71, Echovirus 6, SARS-CoV-2	HCV	Inhibition of viral adsorption and entry; inhibitory activity toward HIV-1 RT; binding of HCV envelop protein;	[325,[328], [329], [330], [331], [332], [333], [334], [335], [336], [337], [338], [339], [340], [341], [342], [343],^362^,^363^]
Lactadherine	HRoV	HRoV	Inhibition of HRV binding to cells	[[344], [345], [346],^364^]
Mucins	HIV, poxvirus, Norwalk virus, HRoV, SARS-CoV-2	/	MUC1 binds the DC-SIGN receptor, blocking HIV-1 DCs infection—binding inhibition, entry inhibition	[343,[347], [348], [349], [350], [351],^365^]
Oxysterols	HRoV, HRV	/	/	[366]
Tenascin-C (TNC)	HIV-1	/	Interaction with the HIV-1 Envelope (Env) variable 3 (V3) loop	[[367], [368], [369]]
Lipid compounds	ZIKV, HCV, VSV, HSV, DENV, JEV	/	Virucidal activity; alteration of the viral envelope	[83,[352], [353], [354],^370^]
Extracellular vesicles (HM-EVs)	HIV, HCMV, RSV, HRoV, ZIKV, USUV	/	Competition with HIV-1 for binding to DC-SIGN on MDDCs; inhibition of HCMV attachment; inhibition of RSV and HRoV entry	[327,355,356,371]
Secretory leucocyte protease inhibitor (SLPI)	HIV	/	/	[357]
Lewis X	HIV	/	Binding inhibition	[358]
β-Lactoglobulin	HIV	/	Partial inhibition of HIV integrase and reverse transcriptase activity	[339,359]
α-Lactalbumin	SARS-CoV-2	/	Inhibition of viral attachment and entry	[291]

ADNV, Andes virus; ADV, Adenovirus; EV71, enterovirus 71; CVB3, coxsackievirus B3; DENV, dengue virus; EBOV, Ebola virus; FluV, influenza virus; HAV, hepatitis A virus; HBV, hepatitis B virus; HCMW, Human cytomegalovirus; HCV, hepatitis C virus; HEV, hepatitis E virus; HGV, hepatitis G virus; HM, human milk; HPV, human papilloma virus; HRoV, human rotavirus; HSV-1/HSV-2, Herpes simplex virus types 1 and 2; HTLV-I/HTLV-II, human T-lymphotropic virus; JEV, Japanese encephalitis virus; RSV, respiratory syncytial virus; RuV, Rubella virus; SARS-CoV-2, severe acute respiratory syndrome coronavirus 2; TTV, TT virus; USUV, Usutu virus; VZV, Varicella-zoster virus; WNV, West Nile virus; YFV, Yellow fever virus; ZIKV, Zika virus.

Notes: References include English articles with full-text available*.* In vitro and in vivo studies were considered. Only articles that have analyzed the antiviral activity of native compounds, that is, directly extracted from HM, have been included in this study. Articles that analyzed the antiviral properties of synthetic HM compounds or compounds modified by medicinal chemistry techniques have been excluded.

It should be pointed out that only articles that have analyzed the antiviral activity of native compounds, i.e., directly extracted from HM, have been included in this study. Articles that analyzed the antiviral properties of synthetic HM compounds or compounds modified by medicinal chemistry techniques have been excluded.

### Immunoglobulins and immunological factors

The direct antimicrobial action of HM is expressed through all the different types of immunoglobulins, including secretory IgA (SIgA) and SIgG. IgG and IgM antibodies are derived from the maternal immune response, and an infant will therefore be passively immunized during the period its own immune system matures. The antibody levels reflect the infant’s needs, and their concentrations therefore decrease within lactation. A set of innate, multifunctional molecules also provide significant protection against infections. Factors such as Toll-like receptors (TLR-2 and TLR-4) provide efficient microbial recognition, and work in synergy with the CD14 co-receptor and soluble CD14. Cytokines and chemokines, as well as a variety of cells, including macrophages, T cells, stem cells, and lymphocytes, can also be included in the list of immunologic factors detected in HM. For a detailed discussion of HM immunological components, the readers can refer to some previously published and highly cited works [17,[372], [373], [374]]. In the current review, we decided to focus on the less investigated HM aspecific antiviral components. Nevertheless, considering the most recent literature, special attention should be paid to the anti-SARS-CoV-2 immune components in HM. Sixty-one out of 89 women from 10 studies showed anti-SARS-CoV-2 antibodies in their milk, predominantly IgA [290], and a second systematic review [291] that summarized data from 161 lactating mothers with COVID-19 infection reported that, despite a mild or asymptomatic form of the disease, most mothers produced detectable SARS-CoV-2 specific antibodies in their milk, thus suggesting that breastmilk has the ability to neutralize SARS-CoV-2 activity. In addition, it has been reported that SARS-CoV-2 antibodies in HM remain detectable for at least 10 mo after infection [375]. Finally, only a few studies have investigated the antibody response of HM following mRNA vaccination [[375], [376], [377], [378], [379]]. Mothers who received SARS-CoV-2 mRNA vaccines [377,378] showed a robust secretion of IgA, IgM, and IgG in their milk against this virus for up to 6 wk after the vaccination. However, the composition and neutralization activity of the HM antibody differ between COVID+ lactating mothers and vaccinated ones. Infection is associated with a highly variable IgA-dominant response, whereas vaccination is associated with an IgG-dominant response [379].

### Nonimmunological components of HM endowed with antiviral properties

#### Oligosaccharides and polysaccharides

Human milk oligosaccharides (HMOs) are multifunctional and complex carbohydrates (3–15 units), and they represent the third largest class of HM components, after lactose and lipids. Their concentrations vary between 5–20 g/L. More than 200 HMO structures have been identified, with some maternal factors, such as genetics and the stage of lactation, determining the amount and composition of HMO. Each oligosaccharide is built on a lactose backbone, is expanded by the addition of galactose, N-acetylglucosamine, fucose, or sialic acid, and is branched and elongated in different ways to generate ∼200 different structures [380,381]. HM-glycosaminoglycans (HM-GAGs) are another major class of complex carbohydrates in HM. They are highly sulfated, complex, linear polysaccharides constituted by repeating disaccharidic units, which are present in HM as a complex mixture made up of chondroitin sulfate/dermatan sulfate, heparan sulfate/heparin, and a minor percentage of hyaluronic acid [382]. Quantitative analyses have shown that their concentrations vary between term and preterm mothers and decrease with lactation. HM collected at 30–45 d postpartum from healthy full-term mothers has been found to contain 0.4 g/L of total GAGs [382,383].

Both HMOs and HM-GAGs have been shown to exert antiviral activity against numerous viruses of pediatric clinical relevance, and the antiviral efficacy of HMOs has also been demonstrated in in vivo models (see Table 3). Despite HMOs having been studied more than HM-GAGs, it has emerged that both components share certain characteristics regarding their antiviral mechanism of action: they act as soluble decoy receptors or as competitive inhibitors to inhibit the attachment of viruses to cells. This mechanism has mostly been attributed to their complex chemical structure and their glycan moieties, which are similar to the glycans on the host cell surface.

Because HMOs have a similar structure to the mucin (MUC) glycans found on the mucosal layer, they can act as soluble decoys, that is, by binding to viruses and preventing their early attachment to cells, or they can exert an indirect action, that is, binding and masking the receptors on the epithelial cell surface, thus avoiding the initiation of viral infection [381]. Similar mechanisms have been proposed for HM-GAGs. Indeed, heparan sulfate proteoglycans (HSPGs), common attachment receptors for different viral pathogens [[4], [5], [6]], are composed of a core protein that is covalently linked to heavily sulfated GAG chains, which can result in competition with HM-GAGs for binding to viral particles. Weichert et al. [360] demonstrated, through X-ray crystallography, that 2 HMOs, that is, 2′-fucosyllactose and 3-fucosyllactose, bound the receptor pockets onto the norovirus capsid, through the structural mimicry of histo-blood group antigens, which are essential binding factors for norovirus infections. Similarly, our group previously demonstrated in vitro, that HM-GAGs are endowed with significant anti-HCMV and anti-respiratory syncytial virus (RSV) activities, and that they are able to alter the binding of a virus to a cell. We showed that the fast-moving heparin is the most active GAG, and that viral inhibition by individual HM-GAGs depends on a specific structural configuration of the GAG [361]. Interestingly, both HMOs and HM-GAGs can reach the intestinal lumen undigested, thus indicating that they are not part of the nutritive components of HM and underlying their role as essential bioactive factors [384,385].

The potential antiviral action of HM-GAGs against SARS-CoV-2 has recently been hypothesized, but an in vitro and/or in vivo demonstration is still lacking [386,387].

#### Lactoferrin

Lactoferrin (Lf) is a highly conserved glycoprotein that belongs to the transferrin family, which is able to reversibly chelate 2 Fe(III) per molecule with high affinity. It is secreted by glandular epithelial cells and by neutrophils. The highest levels (∼8 mg/mL) are found in human colostrum, although it is also present, albeit at lower levels, in mature milk (∼3 mg/mL) in most exocrine secretions, and in the secondary granules of mature neutrophils. Lf is endowed with significant antimicrobial properties in vitro against bacterial, naked, and enveloped viruses (see Table 3). It has emerged that LF mainly acts by binding to HSPGs on the surface of the host cell, thereby reducing viral attachment and any subsequent viral entry, or by binding directly to viral proteins to inhibit viral adsorption on target cells or interfering with the intracellular transport of the virus and intercepting the delivery of viral genomes to the cytoplasm. In addition, Lf has shown the ability to inhibit viral enzymes, such as HIV-1 RT. Despite the antiviral activity of Lf having been well-characterized in vitro, very little evidence is available from in vivo models or clinical trials. Interestingly, Briana et al. [388], who considered the Lf concentration in HM during SARS-CoV-2 infection, found that symptomatic mothers displayed lower Lf concentrations than asymptomatic mothers and healthy controls, thus suggesting that the concentration of this whey protein may be modulated by viral infections, and underlying its potential role as an in vivo antiviral agent. However, another recent study on HCMV has found no correlation between the HCMV load in milk and the Lf concentration, or no major difference in the Lf concentration in the milk of transmitting and nontransmitting women. To the best of our knowledge, only 1 report has suggested that HTLV-1 infection may be able to induce the expression of Lf in a paracrine manner in the lactic compartment, thus indicating that a mutual interaction between HTLV-1 and Lf would benefit from the milk-borne transmission of this virus [362]. Finally, lactoferricin, a pepsin-digested Lf derivative, has also shown in vitro antiviral activity against SARS-CoV-2, HIV, HSV-1, HSV-2, and HCMV [[363], [389], [390], [391], [392], [393]].

#### Extracellular vesicles (HM-EVs)

HM-EVs consist of lipid bilayer-enclosed vesicles that display a heterogeneous size, ranging from 50 to 200 nm, and which are secreted by breast epithelial cells, HM-macrophages, and lymphocytes. EVs can generally be taken up by other cells in which they release their molecular cargo (for example, miRNA, mRNA, RNAs, enzymes, and signaling proteins) and they play a role in intercellular signaling, the immune response, stem cell differentiation, the neuronal function, and tissue regeneration [394]. Recent publications have reported antiviral properties of HM-EVs against both viruses of pediatric clinical relevance and emerging viruses (see Table 3). These studies described a mechanism of competition between HM-EVs and viruses for cell-attachment and/or cell-entry. Näslund et al. [371] reported that HM-exosomes (a specific subclass of cell-derived EVs), bind monocyte-derived dendritic cells (MDDCs) via DC-SIGN, thus preventing HIV-1 infection and its subsequent transfer to CD4+ T cells. In a previous work of ours, we demonstrated that HM-EVs impair the attachment of HCMV to cells. In addition, the proteomic analysis of the EV surfaceome revealed a contribution of immune components (IgA, C9, and C3 proteins) to antiviral activity and identified several proteins which, with their glycomoieties, seem to play a crucial role in the interference mechanism between HCMV and the host cells [395]. In a later work, we also demonstrated that HM-EVs affect the entry of RSV and HRoV into the host cell through a mechanism that remains unclear from the molecular point of view. Some researchers have shown that HM-exosomes are long-lasting in vivo and that they accumulate in the intestinal mucosa, spleen, liver, heart, or brain when administered orally or intravenously to mice and pigs, thus also suggesting a potential antiviral action at distant body sites [396,397].

#### Mucins

MUC are large glycoproteins that consist of 50% of carbohydrates (most of which are O-linked) that are detected in a variety of biofluids in which they confer viscosity proportionally to their concentration. Among the various MUC subtypes, MUC1 and MUC4 are abundant in HM (420 μg/mL, primarily within milk fat globules) and have shown antiviral activity against HIV, HRoV, poxvirus, and Norwalk virus (see Table 3) [364,398]. Their action is related to their sialylated residues, which are very similar to those of the cell membrane and allow them to bind the virus and trap it, thereby preventing cell infection. Their mechanism of action has been well-characterized against HIV. Saeland et al. [365] demonstrated that epithelial mucin MUC1 efficiently binds to the DC-SIGN HIV receptor of dendritic cells (DCs), thus blocking the interaction of the HIV-1 gp120 glycoprotein with DC and preventing the DC-SIGN-mediated transmission of HIV-1 from DCs to CD4+ T cells. Indeed, the O-linked glycans within the MUC domain are known to contain Lewis X structures that are explicitly recognized by DC-SIGN, thereby allowing MUC1 to act as a competitive inhibitor. In addition, it is possible to speculate that MUC plays an indirect antiviral role in determining the increasing viscosity of HM that has been observed for maternal infections by ZIKV [296]. Nevertheless, the antiviral activity of MUC remains to be demonstrated through in vivo models.

#### Lipids

HM lipids are the second largest class of HM components after lactose. They are present in large vesicles and consist of triacylglycerols surrounded by a phospholipid membrane. After infant digestion, monoglycerides and free fatty acids are produced, and they have been suggested to be endowed with antiviral action, depending on their length, degree of saturation, and presence of active radicals. To date, only a few studies have focused on the antiviral activity of free fatty acids in HM (Table 3), and they have shown, in vitro, that their action against enveloped viruses is time and storage-condition dependent. These studies generally indicated that the milk fat globule membrane (MFGM) is altered upon refrigeration, thus exposing the triglyceridic core to milk lipases and determining a time-dependent production of free fatty acids. The latter would then alter the integrity of the viral envelope, by destroying the viral particle. Interestingly, recent studies have investigated the lipid content in HM considering the mother’s infections. Gardner et al. [399] showed that some changes take place in the fatty acid composition of HM in response to cold-like symptoms, and Mabaya et al. [400] reported deficiencies in breast milk omega-3 fatty acids in HIV-infected women. Overall, these studies clearly suggested a potential involvement of HM lipid components in the protection of infants against infection.

##### Other antiviral components

###### Oxysterols

Oxysterols are cholesterol oxidation derivatives that can be detected in human blood circulation. 25-hydroxycholesterol (25OHC) and 27-hydroxycholesterol (27OHC) are known to be endowed with a broad spectrum of antiviral activities [366]. However, only one report [366] has indicated their presence in HM to date, and has shown that 25OHC, 27OHC, and 24S-hydroxycholesterol are detectable at all the different stages of lactation, with a remarkable peak of 27OHC in colostrum (126 μg/L for 27OHC, 11 μg/L for 24OHC, and 9 μg/L for 25OHC). Antiviral assays have revealed that colostrum contains a concentration of 27OHC, which has been able to inhibit HRoV and HRV in vitro, thus suggesting that a passive transfer of this molecule to an infant via breastfeeding might be protective.

###### Tenascin C

Tenascin C (TNC) is a large, hexameric, extracellular matrix glycoprotein that is involved in fetal development and wound healing, and which is found in HM and genital fluids. HM-TNC has been associated with the anti-HIV activity of this biofluid, and it is known to contribute to the previously described phenomenon. It has been found that more than 90% of infants exposed to HIV-1 via breastfeeding remain uninfected. Fouda et al. [367] demonstrated that TNC neutralizes HIV-1 variants by binding HIV-1 envelope protein (Env) to a site that is induced upon engagement of its primary receptor, CD4. Subsequently, Mangan et al. [368] showed that this anti-HIV activity is dependent on the TNC fibrinogen-like globe and fibronectin-type III domains, oligomerization, and the glycan structure, and they also identified critical Env V3 residues. Despite this in vitro evidence, Mansour et al. [369], in 2016, found that that an endogenous TNC concentration in mucosal fluids may be inadequate to block HIV-1 transmission in vivo.

###### Lactadherine

Few reports are available about the antiviral activity of lactadherine, an MFGM glycoprotein that has been detected in a concentration of ∼70 μg/mL in an HM sample [364]. In that work, its antiviral action against HRoV (Table 3), through a mechanism that involves the inhibition of viral binding to cells, was demonstrated in vitro.

Secretory Leucocyte Protease Inhibitor (SLPI), Lewis X antigen, α-Lactalbumin and β-Lactoglobulin have been included in Table 3, despite the lack of information about their antiviral activity. However, it should be underlined that numerous studies have reported that the antiviral activity and the spectrum of action of α-lactalbumin and β-lactoglobulin can be enhanced by specific chemical modifications of the native compound.

### HM Pasteurization and Storage Methods

#### The impact of pasteurization and storage methods on the viral load and detectable live viruses in HM

Already back in 1979, Holder pasteurization (HoP) was described as being able to reduce contamination in HM by pathogenic bacteria and viruses, and that it decreased the concentration and activity of HM protective factors [401]. Later, Goldblum et al. [402] demonstrated that heating HM at 72°C for 5–15 s could eliminate HCMV, whereas many of the immunological and nutritional components were preserved. Deodhar et al. showed that freezing (5 d at −20°C) HM was not sufficient to eradicate microorganisms. Hamprecht et al. [403] have recently compared 3 different HM treatments for the eradication of HCMV: *1*) freezing (−20°C over a period of 18 h–10 d), *2*) HoP (62.5°C for 30 min), and *3*) a new high-temperature short-time (HTST) pasteurization treatment (72°C for 5–10 s). Both of the thermal treatments were able to eliminate viral infectivity, whereas the freezing treatment preserved the vRNA.

Other studies have found that freezing HM reduces infectivity, but it does not completely prevent viral transmission [110,[278], [279], [280], [281], [404], [405], [406]]. Donalisio et al. [407] have recently confirmed that HM freezing partially reduces the viral concentration and that HoP also reduces the biological antiviral component contents in the milk.

However, many studies [[282], [283], [408], [409], [410], [411], [412], [413]] have demonstrated that the storage of HM at −20°C for more than 3 d reduces the HCMV titer by 78%–99%, and that storage for more than 7 d reduces HCMV infectivity by 90%–100%.

Microwave and UV-C radiation processing have also been used successfully to prevent HCMV transmission. [[414], [415], [416], [417]].

These contradicting results may depend on the different viral loads in the HM before the treatment and on different characteristics of the considered infants. A low viral load, a heavy weight, and an advanced gestational age minimize the chances of infection, thus even making frozen milk (from 3 to 7 d) safe.

It has recently been demonstrated that HTST pasteurization (72°C for 10 s) is effective in eradicating bacteria- and lipid-enveloped viruses [418]. Klotz et al. [419] showed that HTST inactivated HCMV in HM samples, but it was not as effective as standard HoP. Bapistella et al. [420] demonstrated that the reduction in the transmission rate in very preterm infants is similar for HoP and HTST pasteurization. Maschmann et al. [421]. found that very short-term low-temperature (VSTLT) pasteurization (62°C for 5 s) was effective in inactivating HCMV [422]. It has also been found that a variant of HoP (30 min at 60°C) is able to eradicate HCMV [423].

Consistent evidence on the heat instability of HIV-1 in culture media and body fluids, including HM, has indicated that the pasteurization of breastmilk inactivates HIV-1 [424]. In 2001, Jeffery et al. [425] described pretoria pasteurization as a method by which HIV-infected women could express and pasteurize their breastmilk in a domestic setting. Flash heating, which involves heating HM in a water bath up to 100 °C, and immediately cooling it to 37.0°C [426], seems to be effective in reducing HIV infectivity, although the domestic nature of these methods makes it difficult to draw unambiguous conclusions (211). Hoque et al. [427] have recently demonstrated that heating milk at 65°C for 5 s (VSTLT) in a pan over a stove inhibits HIV transmission and helps to retain the key nutritional elements of the milk.

Ando et al. [[428], [429], [430]] demonstrated that HTLV transmission was prevented by freezing HM overnight. Hamilton Spence et al. [431] demonstrated that the HoP procedure eradicated EBOV and MARV inoculated into donor HM [432]. Donalisio et al. [433] verified that the HoP procedure eliminated both high-risk and low-risk HPV from donor HM. de Oliveira et al. [434] proved that HoP was not able to eliminate HBV infectious from HM, thus supporting the recommendation for serologic screening for HBV in HM Banks.

**FIGURE 4:**
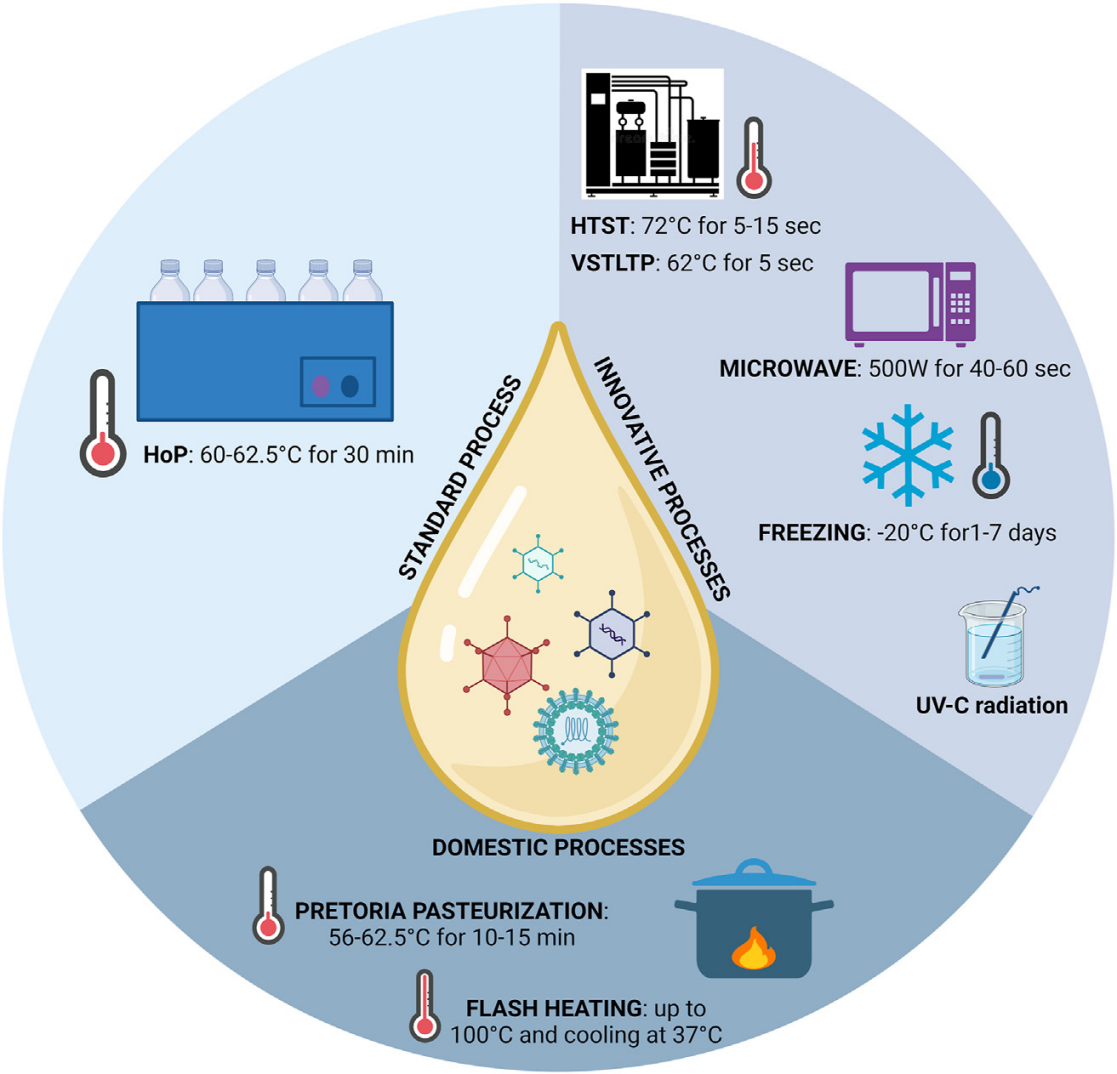
Overview of human milk pasteurization processes.

In the last 2 y, many studies performed on SARS-CoV-2 have revealed that when control samples that were spiked with the replication-competent SARS-CoV-2 virus were treated with HoP, no replication-competent virus or vRNA was detectable [72,[435], [436], [437], [438]]. Table 4 [439,440] and Figure 4 schematically summarize the analyzed viral inactivation methods.

**TABLE 4:** Impact of pasteurization and storage methods on the inactivation of viruses in HM and on the milk value

Virus	Inactivation method	Efficacy	Preservation of the milk value	References on efficacy
HCMV	HoP (60–62.5°C for 30 min)	**+**	**+/−**	[110,395,[278], [279], [280], [402], [403],^404^,^405^,[282], [283], [408], [409], [410], [411], [412],[418], [419], [420],^422^,^439^]
	HTST (72°C for 5–15 s)	**+**	**+/−**	
	Freezing (−20°C, for 3–7 d)	**+/−**	**+**	
	Microwave (500 W, 40–60 s)	**+/−**	nd	[414,415]
	UV-C radiation	**+**	nd	[416,417]
	VSTLTP (62°C for 5 s)	**+**	+	[421]
HIV	HoP (62.5°C for 30 min)	**+**	nd	[423]
	Domestic Pretoria Pastorization (56-62.5°C for 10–15 min)	+/−	nd	[424,440]
	Domestic flash heating (up to 100°C and immediate cooling at 37°C)	**+/−**	+/−	[425,426]
	Domestic VSTLTP (62°C for 5 s)	+/−	+	[427]
HTLV	Freezing (−20°C, overnight)	+	nd	[[428], [429], [430]]
EBOV and MARV	HoP (62.5°C for 30 min)	+	nd	[431,432]
HPV	HoP (62.5°C for 30 min)	+	nd	[433]
HBV	HoP (62.5°C for 30 min)	−	nd	[434]
SARS-CoV-2	HoP (62.5°C for 30 min)	+	nd	[72,[435], [436], [437], [438]]

+, total inactivation; +/−, partial inactivation; −, not active; nd, not determined; EBOV, Ebola virus;

HBV, hepatitis B virus; HCMV, human cytomegalovirus; HM, human milk; HPV, human papilloma virus; HTLV, human T-lymphotropic virus; MARV, Marburg virus; SARS-CoV-2, severe acute respiratory syndrome coronavirus 2.

Notes: References include English articles with full-text available*.* In vitro and in vivo studies were considered.

#### Impact of pasteurization and storage methods on the antiviral action of HM

In 1993, Brüssow et al. [441] demonstrated that the antirotavirus activity of HM was maintained after heating at 80°C for 10 min. Orloff et al. [423] suggested that the inactivating activity of HIV-1 was not removed by the pasteurization process, whereas Giles et al. [442] reported that both a heat treatment at 60 °C for 30 min and HoP significantly reduced the IgA, LF, lysozyme, vitamin C and B6 contents. Chantry et al. [443], in 2012, considering HIV-positive mothers in developing countries, demonstrated that flash heating protocols could preserve most of the IgA and IgG in HM, and suggested that this method could be immunologically better than boiling milk or using breastmilk substitutes. Verd et al. [444], demonstrated that the antibodies against VZV in HM are stable, even after 12 mo at −20°C, and that the oral administration of immunoglobulin can provide passive immunity as it is resistant to proteolytic digestion.

Pfaender et al. [370] and Conzelmann et al. [445] showed that pasteurized or stored HM, but not fresh HM, was able to inactivate ZIKV, and that this effect seems to be dependent on the free fatty acids released by milk lipases. Conzelmann et al. found that the antiviral activity was in the fat-containing cream fraction of HM and resulted in the destruction of the structural integrity of viral particles.

In 2018, Donalisio et al. [407] showed that HM treated by means of HTST preserved its antiviral activity against HCMV, RSV, HRoV, and HSV-2, whereas HTST and HoP both reduced the antiviral activity against HRV and HSV-1 to a great extent Unexpectedly, they found that HoP can even improve the anti-HRoV activity in HM, but, overall, HTST was better at preserving the biological properties of HM. The anti-HCMV activity of colostrum has resulted to be reduced to a great extent by HoP [395].

In 2021, Perez et al. [446] demonstrated that pasteurization did not affect IgG or the neutralizing activity against SARS-CoV-2, but it did affect the IgM and IgA levels). Moreover, donor milk from vaccinated mothers seemed to retain IgG and its neutralizing activity. Francese et al. [361] with the aim of elucidating different mechanisms, other than immunoglobulin-mediated one, demonstrated that HM glycosaminoglycans could contribute to the overall antiviral activity of HM by exerting a synergic action with other antiviral agents, and that there was no reduction after a HoP treatment

Van Keulen et al. [447] confirmed that, after HoP, the total anti-SARS-CoV-2 IgA levels decreased, whereas HM maintained its neutralization capacity after a high-pressure pasteurization (HPP) treatment. Kothari et al. [448] observed that HPP-treated milk maintained an anti-HCMV activity that was comparable with that of raw milk and HoP milk.

## Discussion and Conclusion

Considering the high frequency of the detection of viral pathogens in HM and, at the same time, the plethora of antiviral factors, what is reasonable to conclude? Is breast milk a biofluid that transmits viruses or protects against their infection?

The following concepts can be highlighted from the considered literature:

- From a long list of viruses, only viral nucleic acids have been detected in HM till now; among these, just a few viruses have shown the ability to replicate in a cell culture (Table 1). The detection of viral components and/or infected cells in HM may be a consequence of an effective viral replication in this biofluid in the mammary gland or, alternatively, may reflect an origin from the vascular compartment with the spreading of viruses/infected cells to the mammary gland or milk.
- HCMV, HIV, HTLV, and EBOV are the only viruses for which an MTCT through breastfeeding has been demonstrated, but the rate of transmission, especially for HIV and HCMV, is generally lower than expected. Transmission through HM is rare or still uncertain for most other viruses. Demonstrating that HM is the route of transmission of clinically relevant viral pathogens is in fact a challenging task in the neonatal period, and it requires the exclusion of other potential and/or highly effective routes of transmission.
- Several antiviral components, which are still partially undiscovered, mediate the protective activity of HM. The most frequent antiviral mechanism of action is the inhibition of the early stages of viral replication. These HM components are frequently endowed with the ability to bind viral or cellular receptors, thus preventing virus binding and blocking entry into the host cell. Most of the conducted studies have been performed in vitro, and the specific in vivo antiviral action of numerous compounds still remains to be validated. In addition, we have observed that the antiviral activity of the reported biofactors is usually partial, that is, they are not able to completely inhibit a viral infection. This suggests that they may cooperate in mediating an antiviral action in HM.
- Although it has clearly been stated that the antiviral properties of HM are inactivated by extreme heating, conflicting evidence has emerged on the pasteurization effect (HoP and HTST). Several studies on HIV and HCMV have shown that freezing reduces the infectivity of HM, but it does not completely prevent viral transmission. However, pasteurization is able to inactivate these pathogens. Only a few studies have been published on the other viruses.

Overall, the knowledge on the potential transmission of viral pathogens though breastfeeding and on the antiviral properties of HM is currently at an advanced stage. Nevertheless, further studies are needed to clarify risk/benefit ratio of breastfeeding for some known or emerging maternal infections and to analyze how the bioactive components cooperate in mediating the antiviral action of HM. The current evidence suggests that, in most cases, it is unnecessarily to deprive an infant of this high-quality nourishment and that the continuation of breastfeeding is in the best interest of the infant and the mother.

### Acknowledgments

This review is dedicated to Rebecca. We thank Dr Marguerite Jones for English revision. Figures were created with Biorender.com.

### Author contributions

The authors’ responsibilities were as follows—DL, GM, EB, LC, AC, PT: conceived the project and supervised the study; RF, CP, MD, NC: collected the data; NC: provided the essential materials; all authors: analyzed the data; RF, CP, MD, CL, SC, PT, GM: wrote the paper; DL, GM, EB, LC, AC: had primary responsibility of the final content; and all authors: read and approved the final version of the manuscript.

### Conflict of interest

The authors report no conflicts of interest.

### Funding

This investigation was supported by a research grant from the Fondazione Iolanda Minoli Onlus.

### Data availability

Data described in the manuscript are publicly and freely available without restriction.

## References

[1] Section on Breastfeeding (2012). Breastfeeding and the use of human milk. Pediatrics.

[2] Mazurier E., Rigourd V., Perez P., Buffin R., Couedelo L., Vaysse C. (2017). Effects of maternal supplementation with Omega-3 precursors on human milk composition. J. Hum. Lact..

[3] Smilowitz J.T., O’Sullivan A., Barile D., German J.B., Lönnerdal B., Slupsky C.M. (2013). The human milk metabolome reveals diverse oligosaccharide profiles. J. Nutr..

[4] Global strategy for infant and young child feeding [Internet] [cited October 19, 2022]. Available from: https://www.who.int/publications-detail-redirect/9241562218.

[5] Chantry C.J., Eglash A., Labbok M. (2015). ABM position on breastfeeding-revised 2015. Breastfeed Med.

[6] Butts C.A., Hedderley D.I., Herath T.D., Paturi G., Glyn-Jones S., Wiens F. (2018). Human milk composition and dietary intakes of breastfeeding women of different ethnicity from the manawatu-wanganui region of New Zealand. Nutrients.

[7] Bernardo H., Cesar V., Organization W.H. (2013). https://apps.who.int/iris/handle/10665/79198.

[8] de Silva A., Jones P.W., Spencer S.A. (2004). Does human milk reduce infection rates in preterm infants? A systematic review. Arch. Dis. Child Fetal. Neonatal. Ed..

[9] Walker A. (2010). Breast milk as the gold standard for protective nutrients. J. Pediatr..

[10] Van de Perre P., Molès J.P., Nagot N., Tuaillon E., Ceccaldi P.E., Goga A. (2021). Revisiting Koch’s postulate to determine the plausibility of viral transmission by human milk. Pediatr. Allergy Immunol..

[11] Li H.C., Biggar R.J., Miley W.J., Maloney E.M., Cranston B., Hanchard B. (2004). Provirus load in breast milk and risk of mother -to- child transmission of human T lymphotropic virus type I. J. Infect. Dis..

[12] Takeuchi H., Takahashi M., Norose Y., Takeshita T., Fukunaga Y., Takahashi H. (2010). Transformation of breast milk macrophages by HTLV-I: implications for HTLV-I transmission via breastfeeding. Biomed. Res..

[13] Diaz S., Boulle N., Molès J.P., Peries M., Rutagwera D., Kankasa C. (2018). Human papillomavirus (HPV) shedding in breast milk from African women living with HIV. J. Clin. Virol..

[14] Lanzieri T.M., Dollard S.C., Josephson C.D., Schmid D.S., Bialek S.R. (2013). Breast milk-acquired cytomegalovirus infection and disease in VLBW and premature infants. Pediatrics.

[15] Kurath S., Halwachs-Baumann G., Müller W., Resch B. (2010). Transmission of cytomegalovirus via breast milk to the prematurely born infant: a systematic review. Clin. Microbiol. Infect..

[16] Henrick B.M., Yao X.D., Nasser L., Roozrogousheh A., Rosenthal K.L. (2017). Breastfeeding behaviors and the innate immune system of human milk: working together to protect infants against inflammation, HIV-1, and other infections. Front Immunol.

[17] Ballard O., Morrow A.L. (2013). Human milk composition: nutrients and bioactive factors. Pediatr. Clin. North Am..

[18] Davis N.L., Miller W.C., Hudgens M.G., Chasela C.S., Sichali D., Kayira D. (2016). Maternal and breastmilk viral load: impacts of adherence on peripartum HIV infections averted-the breastfeeding, antiretrovirals, and nutrition study. J. Acquir. Immune. Defic. Syndr..

[19] Van de Perre P. (2000). Breast milk transmission of HIV-1. Laboratory and clinical studies. Ann. N. Y. Acad. Sci..

[20] Rutagwera D.G., Molès J.P., Kankasa C., Mwiya M., Tuaillon E., Peries M. (2019). Prevalence and determinants of HIV shedding in breast milk during continued breastfeeding among Zambian mothers not on antiretroviral treatment (ART): a cross-sectional study. Medicine (Baltimore).

[21] Southern S., Southern P. (2002). Cellular mechanism for milk-borne transmission of HIV and HTLV. Adv. Exp. Med. Biol..

[22] Van de Perre P., Rubbo P.A., Viljoen J., Nagot N., Tylleskär T., Lepage P. (2012). HIV-1 reservoirs in breast milk and challenges to elimination of breast-feeding transmission of HIV-1. Sci. Transl. Med..

[23] Rousseau C.M., Nduati R.W., Richardson B.A., Steele M.S., John-Stewart G.C., Mbori-Ngacha D.A. (2003). Longitudinal analysis of human immunodeficiency virus type 1 RNA in breast milk and of its relationship to infant infection and maternal disease. J. Infect. Dis..

[24] Hoffman I.F., Martinson F.E.A., Stewart P.W., Chilongozi D.A., Leu S.Y., Kazembe P.N. (2003). Human immunodeficiency virus type 1 RNA in breast-milk components. J. Infect. Dis..

[25] Becquart P., Petitjean G., Tabaa Y.A., Valéa D., Huguet M.F., Tuaillon E. (2006). Detection of a large T-cell reservoir able to replicate HIV-1 actively in breast milk. AIDS.

[26] Lewis P., Nduati R., Kreiss J.K., John G.C., Richardson B.A., Mbori-Ngacha D. (1998). Cell-free human immunodeficiency virus type 1 in breast milk. J. Infect. Dis..

[27] Petitjean G., Becquart P., Tuaillon E., Al Tabaa Y., Valea D., Huguet M.F. (2007). Isolation and characterization of HIV-1-infected resting CD4+ T lymphocytes in breast milk. J. Clin. Virol..

[28] Gray R.R., Salemi M., Lowe A., Nakamura K.J., Decker W.D., Sinkala M. (2011). Multiple independent lineages of HIV-1 persist in breast milk and plasma. AIDS.

[29] Goga A.E., Van de Perre P., Ngandu N., Nagot N., Abrams E.J., Moodley D. (2021). Eliminating HIV transmission through breast milk from women taking antiretroviral drugs. BMJ.

[30] Buranasin P., Kunakorn M., Petchclai B., Raksakait K., Wichukchinda N., Jirapinyo M. (1993). Detection of human immunodeficiency virus type 1 (HIV-1) proviral DNA in breast milk and colostrum of seropositive mothers. J. Med. Assoc. Thai..

[31] Guay L.A., Hom D.L., Mmiro F., Piwowar E.M., Kabengera S., Parsons J. (1996). Detection of human immunodeficiency virus type 1 (HIV-1) DNA and p24 antigen in breast milk of HIV-1-infected Ugandan women and vertical transmission. Pediatrics.

[32] Kinoshita K., Hino S., Amagaski T., Ikeda S., Yamada Y., Suzuyama J. (1984). Demonstration of adult T-cell leukemia virus antigen in milk from three sero-positive mothers. Gan.

[33] Ando Y., Nakano S., Saito K., Shimamoto I., Ichijo M., Toyama T. (1987). Transmission of adult T-cell leukemia retrovirus (HTLV-I) from mother to child: comparison of bottle- with breast-fed babies. Jpn J. Cancer Res..

[34] Yoshinaga M., Yashiki S., Oki T., Fujiyoshi T., Nagata Y., Sonoda S. (1995). A maternal risk factor for mother-to-child HTLV-I transmission: viral antigen-producing capacities in culture of peripheral blood and breast milk cells. Jpn J. Cancer Res..

[35] Matsubara F., Haraguchi K., Harada K., Koizumi A. (2012). Screening for antibodies to human T-cell leukemia virus type I in Japanese breast milk. Biol. Pharm. Bull..

[36] Nagamine M., Nakashima Y., Uemura S., Takei H., Toda T., Maehama T. (1991). DNA Amplification of Human T Lymphotropic Virus Type 1 (HTLV-I) Proviral DNA in breast milk of HTLV-I carriers. J. Infect. Dis..

[37] Heneine W., Woods T., Green D., Fukuda K., Giusti R., Castillo L. (1992). Detection of HTLV-II in breastmilk of HTLV-II infected mothers. Lancet.

[38] Pimenta F.C.F., Kashima Haddad S., de Medeiros Filho J.G., Costa M.J.C., Diniz M.F.M., Fernandes M.P. (2008). Prevalence ratio of HTLV-1 in nursing mothers from the state of Paraiba, Northeastern Brazil. J. Hum. Lact..

[39] Millen S., Thoma-Kress A.K. (2022). Milk transmission of HTLV-1 and the need for innovative prevention strategies. Front. Med. (Lausanne)..

[40] Nartey T., Moran H., Marin T., Arcaro K.F., Anderton D.L., Etkind P. (2014). Human mammary tumor virus (HMTV) sequences in human milk. Infect. Agent. Cancer..

[41] Hayes K., Danks D.M., Gibas H., Jack I. (1972). Cytomegalovirus in human milk. N. Engl. J. Med..

[42] Hotsubo T., Nagata N., Shimada M., Yoshida K., Fujinaga K., Chiba S. (1994). detection of human cytomegalovirus DNA in breast milk by means of polymerase chain reaction. Microbiol. Immunol..

[43] Schleiss M.R. (2006). Role of breast milk in acquisition of cytomegalovirus infection: recent advances. Curr. Opin. Pediatr..

[44] Bardanzellu F., Fanos V., Reali A. (2019). Human breast milk-acquired cytomegalovirus infection: certainties, doubts and perspectives. Curr. Pediatr. Rev..

[45] Meier J., Lienicke U., Tschirch E., Krüger D.H., Wauer R.R., Prösch S. (2005). Human cytomegalovirus reactivation during lactation and mother-to-child transmission in preterm infants. J. Clin. Microbiol..

[46] Lazar K., Rabe T., Goelz R., Hamprecht K. (2020). Human cytomegalovirus reactivation during lactation: impact of antibody kinetics and neutralization in blood and breast milk. Nutrients.

[47] Hamprecht K., Witzel S., Maschmann J., Dietz K., Baumeister A., Mikeler E. (2003). Rapid detection and quantification of cell free cytomegalovirus by a high-speed centrifugation-based microculture assay: comparison to longitudinally analyzed viral DNA load and pp67 late transcript during lactation. J. Clin. Virol..

[48] Hamprecht K., Goelz R., Maschmann J. (2005). Breast milk and cytomegalovirus infection in preterm infants. Early Hum. Dev..

[49] Hamprecht K., Maschmann J., Vochem M., Dietz K., Speer C.P., Jahn G. (2001). Epidemiology of transmission of cytomegalovirus from mother to preterm infant by breastfeeding. Lancet.

[50] Maschmann J., Goelz R., Witzel S., Strittmatter U., Steinmassl M., Jahn G. (2015). Characterization of human breast milk leukocytes and their potential role in cytomegalovirus transmission to newborns. Neonatology.

[51] Hernandez-Alvarado N., Shanley R., Schleiss M.R., Ericksen J., Wassenaar J., Webo L. (2021). Clinical, virologic and immunologic correlates of breast milk acquired cytomegalovirus (CMV) infections in very low birth weight (VLBW) infants in a newborn intensive care unit (NICU) setting. Viruses.

[52] Hamprecht K., Goelz R. (2017). Postnatal cytomegalovirus infection through human milk in preterm infants: transmission, clinical presentation, and prevention. Clin. Perinatol..

[53] Lanari M., Capretti M.G., Lazzarotto T., Gabrielli L., Pignatelli S., Monte P.D. (2008). Cytomegalovirus infection via mother’s milk: could distinct virus strains determine different disease patterns in preterm twins?. New Microbiol.

[54] Maqsood R., Reus J.B., Wu L.I., Holland L.A., Nduati R., Mbori-Ngacha D. (2021). Breast milk virome and bacterial microbiome resilience in kenyan women living with HIV. mSystems.

[55] Suárez N.M., Musonda K.G., Escriva E., Njenga M., Agbueze A., Camiolo S. (2019). Multiple- strain infections of human cytomegalovirus with high genomic diversity are common in breast milk from human immunodeficiency virus – infected women in zambia. J. Infect. Dis..

[56] Götting J., Lazar K., Suárez N.M., Steinbrück L., Rabe T., Goelz R. (2021). Human cytomegalovirus genome diversity in longitudinally collected breast milk samples. Front Cell. Infect. Microbiol..

[57] Min X., Wang L., Cui A., Zhang C., Wang D., Liu Y. (2020). The nucleic acid positive rate and genotype distribution of human cytomegalovirus in human milk banks in China. Arch. Virol..

[58] Lawrence R.M., Lawrence R.A. (2004). Breast milk and infection. Clin. Perinatol..

[59] Kotronias D., Kapranos N. (1999). Detection of herpes simplex virus DNA in maternal breast milk by in situ hybridization with tyramide signal amplification. In Vivo.

[60] Pietrasanta C., Ghirardi B., Manca M.F., Uccella S., Gualdi C., Tota E. (2014). [Herpesviruses and breast milk]. Pediatr. Med. Chir..

[61] Joshi P.J., Merchant R.H., Pokharankar S.L., Damania K.S., Gilada I.S., Mukhopadhyaya R. (2000). Perinatally cotransmitted human herpesvirus 6 is activated in children born with human immunodeficiency virus infection. J. Hum. Virol..

[62] Fujisaki H., Tanaka-Taya K., Tanabe H., Hara T., Miyoshi H., Okada S. (1998). Detection of human herpesvirus 7 (HHV-7) DNA in breast milk by polymerase chain reaction and prevalence of HHV-7 antibody in breast-fed and bottle-fed children. J. Med. Virol..

[63] Brayfield B.P., Kankasa C., West J.T., Muyanga J., Bhat G., Klaskala W. (2004). Distribution of Kaposi sarcoma-associated herpesvirus/human herpesvirus 8 in maternal saliva and breast milk in Zambia: implications for transmission. J. Infect. Dis..

[64] Dedicoat M., Newton R., Alkharsah K.R., Sheldon J., Szabados I., Ndlovu B. (2004). Mother-to-child transmission of human herpesvirus-8 in South Africa. J. Infect. Dis..

[65] Junker A.K., Thomas E.E., Radcliffe A., Forsyth R.B., Davidson A.G.F., Rymo L. (1991). Epstein- barr virus shedding in breast milk. Am. J. Med. Sci..

[66] Gantt S., Carlsson J., Shetty A.K., Seidel K.D., Qin X., Mutsvangwa J. (2008). Cytomegalovirus and Epstein-Barr virus in breast milk are associated with HIV-1 shedding but not with mastitis. AIDS.

[67] Glenn W.K., Whitaker N.J., Lawson J.S. (2012). High risk human papillomavirus and Epstein Barr virus in human breast milk. BMC Res. Notes..

[68] Daud I.I., Coleman C.B., Smith N.A., Ogolla S., Simbiri K., Bukusi E.A. (2015). Breast milk as a potential source of Epstein-Barr virus transmission among infants living in a malaria - endemic region of Kenya. J. Infect. Dis..

[69] Viljoen J., Tuaillon E., Nagot N., Danaviah S., Peries M., Padayachee P. (2015). Cytomegalovirus, and possibly Epstein–Barr virus, shedding in breast milk is associated with HIV-1 transmission by breastfeeding. AIDS.

[70] Sanosyan A., Rutagwera D.G., Molès J.P., Bollore K., Peries M., Kankasa C. (2016). Increased Epstein–Barr virus in breast milk occurs with subclinical mastitis and HIV shedding. Medicine (Baltimore).

[71] Montoya-Ferrer A., Sanosyan A., Fayd’herbe de Maudave A., Pisoni A., Bollore K., Molès J.P. (2021). Clinical and biological factors associated with early Epstein-Barr virus infection in human immunodeficiency virus-exposed uninfected infants in Eastern Uganda. Clin. Infect. Dis..

[72] Chambers C., Krogstad P., Bertrand K., Contreras D., Tobin N.H., Bode L. (2020). Evaluation for SARS-CoV-2 in breast milk from 18 infected women. JAMA.

[73] Krogstad P., Contreras D., Ng H., Tobin N., Chambers C.D., Bertrand K. (2022). No infectious SARS-CoV-2 in breast milk from a cohort of 110 lactating women. Pediatr. Res..

[74] Ribeiro A., de Brasil L.M.C., Prada R., Nogueira J., Maeda A., Sztajnbok J. (2020). Detection of wild-type yellow fever virus in breast milk. Pediatr. Infect. Dis. J..

[75] Arragain L., Dupont-Rouzeyrol M., O’Connor O., Sigur N., Grangeon J.P., Huguon E. (2017). Vertical transmission of dengue virus in the peripartum period and viral kinetics in newborns and breast milk: new data. J. Pediatr. Infect. Dis. Soc..

[76] Barthel A., Gourinat A.C., Cazorla C., Joubert C., Dupont-Rouzeyrol M., Descloux E. (2013). Breast milk as a possible route of vertical transmission of dengue virus?. Clin. Infect. Dis..

[77] Centers for Disease Control and Prevention (CDC) (2002). Possible West Nile virus transmission to an infant through breast-feeding--Michigan. MMWR Morb. Mortal Wkly Rep..

[78] Hinckley A.F., O’Leary D.R., Hayes E.B. (2007). Transmission of west Nile virus through human breast milk seems to be rare. Pediatrics.

[79] Cavalcanti M.G., Cabral-Castro M.J., Gonçalves J.L.S., Santana L.S., Pimenta E.S., Peralta J.M. (2017). Zika virus shedding in human milk during lactation: an unlikely source of infection?. Int. J. Infect. Dis..

[80] Colt S., Garcia-Casal M.N., Peña-Rosas J.P., Finkelstein J.L., Rayco-Solon P., Weise Prinzo Z.C. (2017). Transmission of Zika virus through breast milk and other breastfeeding-related bodily-fluids: a systematic review. PLOS Negl. Trop. Dis..

[81] Sotelo J.R., Sotelo A.B., Sotelo F.J.B., Doi A.M., Pinho J.R.R., Oliveira R de C. (2017). Persistence of Zika virus in breast milk after infection in late stage of pregnancy. Emerg. Infect. Dis..

[82] Dupont-Rouzeyrol M., Biron A., O’Connor O., Huguon E., Descloux E. (2016). Infectious Zika viral particles in breastmilk. Lancet.

[83] Desgraupes S., Jeannin P., Gessain A., Ceccaldi P.E., Vidy A. (2022). Excretion of cell-free and cell-associated Zika virus into breast milk of infected dams and identification of antiviral factors. Viruses.

[84] Centeno-Tablante E., Medina-Rivera M., Finkelstein J.L., Rayco-Solon P., Garcia-Casal M.N., Rogers L. (2020). Transmission of SARS-CoV-2 through breast milk and breastfeeding: a living systematic review. Ann. N. Y. Acad. Sci..

[85] Lin H.H., Kao J.H., Hsu H.Y., Ni Y.H., Chang M.H., Huang S.C. (1995). Absence of infection in breast-fed infants born to hepatitis C virus-infected mothers. J. Pediatr..

[86] Kumar R.M., Shahul S. (1998). Role of breast-feeding in transmission of hepatitis C virus to infants of HCV-infected mothers. J. Hepatol..

[87] Daudi N., Shouval D., Stein-Zamir C., Ackerman Z. (2012). Breastmilk Hepatitis A virus RNA in nursing mothers with acute Hepatitis A virus infection. Breastfeeding Med.

[88] Chang M.L., Tsao K.C., Huang C.C., Yen M.H., Huang C.G., Lin T.Y. (2006). Coxsackievirus B3 in human milk. Pediatr. Infect. Dis. J..

[89] Maus M.V., Posencheg M.A., Geddes K., Elkan M., Peñaranda S., Oberste M.S. (2008). Detection of Echovirus 18 in human breast milk. J. Clin. Microbiol..

[90] Rivero-Juarez A., Frias M., Rodriguez-Cano D., Cuenca-López F., Rivero A. (2016). Isolation of Hepatitis E virus from breast milk during acute infection. Clin. Infect. Dis..

[91] Louvanto K., Sarkola M., Rintala M., Syrjänen K., Grenman S., Syrjänen S. (2017). Breast milk is a potential vehicle for human papillomavirus transmission to oral mucosa of the spouse. Pediatr. Infect. Dis. J..

[92] Nordenstedt H., Bah E.I., de la Vega M.A., Barry M., N’Faly M., Barry M. (2016). Ebola virus in breast milk in an ebola virus–positive mother with twin babies, Guinea. Emerg. Infect. Dis..

[93] Ververs M., Arya A. (2019). Ebola virus disease and breastfeeding: time for attention. Lancet.

[94] Medina-Rivera M., Centeno-Tablante E., Finkelstein J.L., Rayco-Solon P., Peña-Rosas J.P., Garcia-Casal M.N. (2021). Presence of ebola virus in breast milk and risk of mother-to-child transmission: synthesis of evidence. Ann. N. Y. Acad. Sci..

[95] Sissoko D., Keïta M., Diallo B., Aliabadi N., Fitter D.L., Dahl B.A. (2017). Ebola virus persistence in breast milk after no reported illness: a likely source of virus transmission from mother to child. Clin. Infect. Dis..

[96] Campos G., Bandeira A.A., Rocha V.D., Dias J., Carvalho R., Sardi S. (2017). First detection of chikungunya virus in breast milk. Pediatr. Infect. Dis. J..

[97] Ferrés M., Martínez-Valdebenito C., Angulo J., Henríquez C., Vera-Otárola J., Vergara M.J. (2020 Aug). Mother-to-child transmission of andes virus through breast milk, Chile. Emerg Infect. Dis..

[98] Toyoda H., Naruse M., Yokozaki S., Morita K., Nakano I., Itakura A. (1999). Prevalence of infection with TT virus (TTV), a novel DNA virus, in healthy japanese subjects, newborn infants, cord blood and breast milk. J. Infect..

[99] Buimovici-Klein E., Hite R.L., Byrne T., Cooper L.Z. (1977). Isolation of rubella virus in milk after postpartum immunization. J. Pediatr..

[100] Stinson L.F., Sindi A.S.M., Cheema A.S., Lai C.T., Mühlhäusler B.S., Wlodek M.E. (2021). The human milk microbiome: who, what, when, where, why, and how?. Nutr. Rev..

[101] Young G.R., Yew W.C., Nelson A., Bridge S.H., Berrington J.E., Embleton N.D. (2022). Optimisation and application of a novel method to identify bacteriophages in maternal milk and infant stool identifies host - phage communities within preterm infant gut. Front Pediatr.

[102] Dinleyici M., Pérez-Brocal V., Arslanoglu S., Aydemir O., Sevuk Ozumut S., Tekin N. (2021). Human milk virome analysis: changing pattern regarding mode of delivery, birth weight, and lactational stage. Nutrients.

[103] Southern S., Southern P., Davis M.K., Isaacs C.E., Hanson L.Å., Wright A.L. (2002). Integrating Population Outcomes, Biological Mechanisms and Research Methods in the Study of Human Milk and Lactation [Internet].

[104] Yoshida M., Tezuka T., Hiruma M. (1995). Detection of varicella-zoster virus DNA in maternal breast milk from a mother with herpes zoster. Clin. Diagn. Virol..

[105] Lackey K.A., Pace R.M., Williams J.E., Bode L., Donovan S.M., Järvinen K.M. (2020). SARS-CoV-2 and human milk: what is the evidence?. Matern. Child Nutr..

[106] Linnemann C.C., Goldberg S. (1974). Letter: HBAg in breast milk. Lancet.

[107] Zheng Y., Lu Y., Ye Q., Xia Y., Zhou Y., Yao Q. (2011). Should chronic hepatitis B mothers breastfeed? a meta analysis. BMC Public Health.

[108] Lee A.K., Ip H.M., Wong V.C. (1978). Mechanisms of maternal-fetal transmission of hepatitis B virus. J. Infect. Dis..

[109] Lin H.H., Hsu H.Y., Chang M.H., Chen P.J., Chen D.S. (1993). Hepatitis B virus in the colostra of HBeAg-positive carrier mothers. J. Pediatr. Gastroenterol. Nutr..

[110] Huang H., Ning M., Feng J., Chen J., Dai Y., Hu Y. (2022). Hepatitis B viral markers in the human milk of HBsAg-positive mothers: an observational study. J. Hum. Lact..

[111] Montoya-Ferrer A., Zorrilla A., Viljoen J., Molès J., Newell M., Van de Perre P. (2015). High level of HBV DNA virus in the breast milk seems not to contraindicate breastfeeding. Mediterr. J. Hematol. Infect. Dis..

[112] Yoshida K., Furumoto H., Abe A., Kato T., Nishimura M., Kuwahara A. (2011). The possibility of vertical transmission of human papillomavirus through maternal milk. J. Obstetr. Gynaecol..

[113] Cazzaniga M., Gheit T., Casadio C., Khan N., Macis D., Valenti F. (2009). Analysis of the presence of cutaneous and mucosal papillomavirus types in ductal lavage fluid, milk and colostrum to evaluate its role in breast carcinogenesis. Breast Cancer Res. Treat..

[114] Becquet R., Bland R., Leroy V., Rollins N.C., Ekouevi D.K., Coutsoudis A. (2009). Duration, pattern of breastfeeding and postnatal transmission of HIV: pooled analysis of individual data from West and South African cohorts. PLOS ONE.

[115] Bulterys M., Ellington S., Kourtis A.P. (2010). HIV-1 and breastfeeding: biology of transmission and advances in prevention. Clin. Perinatol..

[116] Chang T.S., Wiener J., Dollard S.C., Amin M.M., Ellington S., Chasela C. (2015). Effect of cytomegalovirus infection on breastfeeding transmission of HIV and on the health of infants born to HIV-infected mothers. AIDS.

[117] Coovadia H.M., Rollins N.C., Bland R.M., Little K., Coutsoudis A., Bennish M.L. (2007). Mother-to-child transmission of HIV-1 infection during exclusive breastfeeding in the first 6 months of life: an intervention cohort study. Lancet.

[118] Fiscus S.A., Aldrovandi G.M., Kourtis A.P., Bulterys M. (2012). Human Immunodeficiency Virus type 1 (HIV-1) and Breastfeeding: Science, Research Advances, and Policy [Internet].

[119] Fouda G.G., Mahlokozera T., Salazar-Gonzalez J.F., Salazar M.G., Learn G., Kumar S.B. (2013). Postnatally-transmitted HIV-1 envelope variants have similar neutralization-sensitivity and function to that of nontransmitted breast milk variants. Retrovirology.

[120] Fowler M.G., Kourtis A.P., Aizire J., Onyango-Makumbi C., Bulterys M., Kourtis A.P., Bulterys M. (2012). Human Immunodeficiency Virus type 1 (HIV-1) and Breastfeeding: Science, Research Advances, and Policy [Internet].

[121] Gross M.S., Taylor H.A., Tomori C., Coleman J.S. (2019). Breastfeeding with HIV: an evidence - based case for new policy. J. Law Med. Ethics..

[122] Humphrey J.H., Marinda E., Mutasa K., Moulton L.H., Iliff P.J., Ntozini R. (2010). Mother to child transmission of HIV among Zimbabwean women who seroconverted postnatally: prospective cohort study. BMJ.

[123] John-Stewart G.C. (2007). Breast-feeding and HIV-1 transmission: how risky for how long?. J. Infect. Dis..

[124] John-Stewart G., Nduati R., Kourtis A.P., Bulterys M. (2012). Human Immunodeficiency Virus type 1 (Hiv-1) and Breastfeeding: Science, Research Advances, and Policy [Internet].

[125] Koulinska I.N., Villamor E., Chaplin B., Msamanga G., Fawzi W., Renjifo B. (2006). Transmission of cell-free and cell-associated HIV-1 through breast-feeding. J. Acquir. Immune. Defic. Syndr..

[126] Koulinska I.N., Villamor E., Msamanga G., Fawzi W., Blackard J., Renjifo B. (2006). Risk of Hiv-1 transmission by breastfeeding among mothers infected with recombinant and non-recombinant Hiv-1 genotypes. Virus Res.

[127] Kourtis A.P., Butera S., Ibegbu C., Belec L., Duerr A. (2003). Breast milk and Hiv-1: vector of transmission or vehicle of protection?. Lancet Infect. Dis..

[128] Kourtis A.P., de Vincenzi I., Jamieson D.J., Bulterys M., Kourtis A.P., Bulterys M. (2012). Human Immunodeficiency Virus type 1 (HIV-1) and Breastfeeding: Science, Research Advances, and Policy [Internet].

[129] Kourtis A.P., Ibegbu C.C., Wiener J., King C.C., Tegha G., Kamwendo D. (2013). Role of intestinal mucosal integrity in HIV transmission to infants through breast-feeding: the BAN study. J. Infect. Dis..

[130] Lunney K.M., Iliff P., Mutasa K., Ntozini R., Magder L.S., Moulton L.H. (2010). Associations between breast milk viral load, mastitis, exclusive breast-feeding, and postnatal transmission of HIV. Clin. Infect. Dis..

[131] Mangé A., Tuaillon E., Viljoen J., Nagot N., Bendriss S., Bland R.M. (2013). Elevated concentrations of milk β2-microglobulin are associated with increased risk of breastfeeding transmission of HIV-1 (Vertical Transmission Study). J. Proteome. Res..

[132] Manji K.P., Duggan C., Liu E., Bosch R., Kisenge R., Aboud S. (2016). Exclusive breast -feeding protects against mother -to- child transmission of HIV-1 through 12 Months of age in Tanzania. J. Trop. Pediatr..

[133] Mofenson L.M. (2010). Antiretroviral drugs to prevent breastfeeding HIV transmission. Antivir. Ther. (Lond)..

[134] Moseholm E., Weis N. (2020). Women living with HIV in high-income settings and breastfeeding. J. Intern. Med..

[135] Ndirangu J., Viljoen J., Bland R.M., Danaviah S., Thorne C., Van de Perre P. (2012). Cell-free (RNA) and cell-associated (DNA) HIV-1 and postnatal transmission through breastfeeding. PLOS ONE.

[136] Nduati R. (2000). Breastfeeding and HIV-1 infection. A review of current literature. Adv. Exp. Med. Biol..

[137] Njom Nlend A.E., Motaze A.C.N., Sandie A., Fokam J. (2018). HIV-1 transmission and survival according to feeding options in infants born to HIV-infected women in Yaoundé, Cameroon. BMC Pediatr.

[138] de Perre P.V., Rubbo P.A., Viljoen J., Nagot N., Tylleskär T., Lepage P. (2012). HIV-1 reservoirs in breast milk and challenges to elimination of breast - feeding transmission of HIV-1. Science Transl. Med..

[139] Perre P.V.D. (2000). Breast milk transmission of HIV-1 laboratory and clinical studies. Ann. N. Y. Acad. Sci..

[140] Potty R.S., Sinha A., Sethumadhavan R., Isac S., Washington R. (2019). Incidence, prevalence and associated factors of mother-to-child transmission of HIV, among children exposed to maternal HIV, in Belgaum district, Karnataka, India. BMC Public Health.

[141] Rollins N., Coovadia H.M. (2013). Breastfeeding and HIV transmission in the developing world: past, present, future. Curr. Opin. HIV AIDS..

[142] Rousseau C.M., Nduati R.W., Richardson B.A., John-Stewart G.C., Mbori-Ngacha D.A., Kreiss J.K. (2004). Association of levels of HIV-1-infected breast milk cells and risk of mother-to-child transmission. J. Infect. Dis..

[143] Shetty A.K., Maldonado Y. (2013). Antiretroviral drugs to prevent mother-to-child transmission of HIV during breastfeeding. Curr. HIV Res..

[144] Waitt C., Low N., Van de Perre P., Lyons F., Loutfy M., Aebi-Popp K. (2018). Does U=U for breastfeeding mothers and infants? Breastfeeding by mothers on effective treatment for HIV infection in high-income settings. Lancet HIV.

[145] White A.B., Mirjahangir J.F., Horvath H., Anglemyer A., Read J.S. (2014). Antiretroviral interventions for preventing breast milk transmission of HIV. Cochrane Database Syst. Rev..

[146] Ziegler J.B., Cooper D.A., Johnson R.O., Gold J. (1985). Postnatal transmission of AIDS-associated retrovirus from mother to infant. Lancet.

[147] Marazzi M.C., Nielsen-Saines K., Buonomo E., Scarcella P., Germano P., Majid N.A. (2009). Increased infant human immunodeficiency virus-type one free survival at one year of age in sub-saharan Africa with maternal use of highly active antiretroviral therapy during breast-feeding. Pediatr. Infect. Dis. J..

[148] Msellati P., Meda N., Leroy V., Likikouët R., Van de Perre P., Cartoux M. (1999). Safety and acceptability of vaginal disinfection with benzalkonium chloride in HIV infected pregnant women in west Africa: ANRS 049b phase II randomized, double blinded placebo controlled trial. DITRAME Study Group. Sex Transm. Infect..

[149] Ngoma M.S., Misir A., Mutale W., Rampakakis E., Sampalis J.S., Elong A. (2015). Efficacy of WHO recommendation for continued breastfeeding and maternal cART for prevention of perinatal and postnatal HIV transmission in Zambia. J. Int. AIDS Soc..

[150] Peltier C.A., Ndayisaba G.F., Lepage P., van Griensven J., Leroy V., Pharm C.O. (2009). Breastfeeding with maternal antiretroviral therapy or formula feeding to prevent HIV postnatal mother-to-child transmission in Rwanda. AIDS.

[151] Petra Study Team (2002). Efficacy of three short-course regimens of zidovudine and lamivudine in preventing early and late transmission of HIV-1 from mother to child in Tanzania, South Africa, and Uganda (Petra study): a randomised, double-blind, placebo-controlled trial. Lancet.

[152] Read J.S. (2003). American Academy of Pediatrics Committee on Pediatric AIDS. Human milk, breastfeeding, and transmission of human immunodeficiency virus type 1 in the United States. American Academy of Pediatrics Committee on Pediatric AIDS. Pediatrics.

[153] Thakwalakwa C., Phiri A., Rollins N., Heikens G.T., Barnell E.K., Manary M. (2014). Growth and HIV-free survival of HIV-exposed infants in Malawi: a randomized trial of two complementary feeding interventions in the context of maternal antiretroviral therapy. J. Acquir. Immune. Defic. Syndr..

[154] Thior I., Lockman S., Smeaton L.M., Shapiro R.L., Wester C., Heymann S.J. (2006). Breastfeeding plus infant zidovudine prophylaxis for 6 months vs formula feeding plus infant zidovudine for 1 month to reduce mother-to-child HIV transmission in Botswana: a randomized trial: the Mashi Study. JAMA.

[155] Thomas T.K., Masaba R., Borkowf C.B., Ndivo R., Zeh C., Misore A. (2011). Triple-antiretroviral prophylaxis to prevent mother-to-child HIV transmission through breastfeeding--the Kisumu Breastfeeding Study, Kenya: a clinical trial. PLOS Med.

[156] Wiktor S.Z., Ekpini E., Karon J.M., Nkengasong J., Maurice C., Severin S.T. (1999). Short-course oral zidovudine for prevention of mother-to-child transmission of HIV-1 in Abidjan, Côte d’Ivoire: a randomised trial. Lancet.

[157] Alvarez-Uria G., Midde M., Pakam R., Bachu L., Naik P.K. (2012). Effect of formula feeding and breastfeeding on child growth, infant mortality, and HIV transmission in children born to HIV- infected pregnant women who received triple antiretroviral therapy in a resource - limited setting: data from an HIV Cohort Study in India. ISRN Pediatr.

[158] Bertolli J., St Louis M.E., Simonds R.J., Nieburg P., Kamenga M., Brown C. (1996). Estimating the timing of mother-to-child transmission of human immunodeficiency virus in a breast-feeding population in Kinshasa, Zaire. J. Infect. Dis..

[159] Bispo S., Chikhungu L., Rollins N., Siegfried N., Newell M.L. (2017). Postnatal HIV transmission in breastfed infants of HIV-infected women on ART: a systematic review and meta-analysis. J. Int. AIDS Soc..

[160] Coutsoudis A., Pillay K., Spooner E., Kuhn L., Coovadia H.M. (1999). Influence of infant-feeding patterns on early mother-to-child transmission of HIV-1 in Durban, South Africa: a prospective cohort study. South African Vitamin A Study Group. Lancet.

[161] Dabis F., Msellati P., Meda N., Welffens-Ekra C., You B., Manigart O. (1999). 6-month efficacy, tolerance, and acceptability of a short regimen of oral zidovudine to reduce vertical transmission of HIV in breastfed children in Côte d’Ivoire and Burkina Faso: a double-blind placebo-controlled multicentre trial. DITRAME Study Group. DIminution de la Transmission Mère-Enfant. Lancet.

[162] Datta P., Embree J.E., Kreiss J.K., Ndinya-Achola J.O., Braddick M., Temmerman M. (1994). Mother-to-child transmission of human immunodeficiency virus type 1: report from the Nairobi Study. J. Infect. Dis..

[163] Dunn D.T., Newell M.L., Ades A.E., Peckham C.S. (1992). Risk of human immunodeficiency virus type 1 transmission through breastfeeding. Lancet.

[164] Ekpini E.R., Wiktor S.Z., Satten G.A., Adjorlolo-Johnson G.T., Sibailly T.S., Ou C.Y. (1997). Late postnatal mother-to-child transmission of HIV-1 in Abidjan, Côte d’Ivoire. Lancet.

[165] Giuliano M., Andreotti M., Liotta G., Jere H., Sagno J.B., Maulidi M. (2013). Maternal antiretroviral therapy for the prevention of mother-to-child transmission of HIV in Malawi: maternal and infant outcomes two years after delivery. PLOS One.

[166] Humphrey J.H., Iliff P.J., Marinda E.T., Mutasa K., Moulton L.H., Chidawanyika H. (2006). Effects of a single large dose of vitamin A, given during the postpartum period to HIV-positive women and their infants, on child HIV infection, HIV-free survival, and mortality. J. Infect. Dis..

[167] Jackson D.J., Chopra M., Doherty T.M., Colvin M.S., Levin J.B., Willumsen J.F. (2007). Operational effectiveness and 36 week HIV-free survival in the South African programme to prevent mother-to-child transmission of HIV-1. AIDS.

[168] Jamieson D.J., Chasela C.S., Hudgens M.G., King C.C., Kourtis A.P., Kayira D. (2012). Maternal and infant antiretroviral regimens to prevent postnatal HIV-1 transmission: 48-week follow-up of the BAN randomised controlled trial. Lancet.

[169] Kilewo C., Karlsson K., Ngarina M., Massawe A., Lyamuya E., Swai A. (2009). Prevention of mother-to-child transmission of HIV-1 through breastfeeding by treating mothers with triple antiretroviral therapy in Dar es Salaam, Tanzania: the Mitra Plus study. J. Acquir. Immune. Defic. Syndr..

[170] Bittencourt A.L. (1998). Vertical transmission of HTLV-I/II: a review. Rev. Inst. Med. Trop. Sao. Paulo..

[171] Furnia A., Lal R., Maloney E., Wiktor S., Pate E., Rudolph D. (1999). Estimating the time of HTLV-I infection following mother-to-child transmission in a breast-feeding population in Jamaica. J. Med. Virol..

[172] Hino S. (1989). Milk-borne transmission of HTLV-I as a major route in the endemic cycle. Acta. Paediatr. Jpn..

[173] Kinoshita K., Amagasaki T., Hino S., Doi H., Yamanouchi K., Ban N. (1987). Milk-borne transmission of HTLV-I from carrier mothers to their children. Jpn J. Cancer Res..

[174] Lal R.B., Gongora-Biachi R.A., Pardi D., Switzer W.M., Goldman I., Lal A.F. (1993). Evidence for mother-to-child transmission of human T lymphotropic virus type II. J. Infect. Dis..

[175] Leal F.E., Michniowski M., Nixon D.F. (2015). Human T-lymphotropic virus 1, breastfeeding, and antiretroviral therapy. AIDS Res. Hum. Retroviruses..

[176] Oki T., Yoshinaga M., Otsuka H., Miyata K., Sonoda S., Nagata Y. (1992). A sero-epidemiological study on mother-to-child transmission of HTLV-I in southern Kyushu, Japan, Asia Oceania. J. Obstet. Gynaecol..

[177] Paiva A.M., Assone T., Haziot M.E.J., Smid J., Fonseca L.A.M., Luiz O do C. (2018). Risk factors associated with HTLV-1 vertical transmission in Brazil: longer breastfeeding, higher maternal proviral load and previous HTLV-1-infected offspring. Sci. Rep..

[178] Ribeiro M.A., Martins M.L., Teixeira C., Ladeira R., Oliveira M. de F., Januário J.N. (2012). Blocking vertical transmission of human T cell lymphotropic virus type 1 and 2 through breastfeeding interruption. Pediatr. Infect. Dis. J..

[179] Weiss R.A. (1993). Milk-borne transmission of HTLV-1. Jpn J. Cancer Res..

[180] Wiktor S.Z., Pate E.J., Rosenberg P.S., Barnett M., Palmer P., Medeiros D. (1997). Mother-to-child transmission of human T-cell lymphotropic virus type I associated with prolonged breast-feeding. J. Hum. Virol..

[181] Fujiyama C., Fujiyoshi T., Miura T., Yashiki S., Matsumoto D., Zaninovic V. (1993). A new endemic focus of human T lymphotropic virus type II carriers among Orinoco natives in Colombia. J. Infect. Dis..

[182] Ishak R., Harrington W.J., Azevedo V.N., Eiraku N., Ishak M.O., Guerreiro J.F. (1995). Identification of human T cell lymphotropic virus type IIa infection in the Kayapo, an indigenous population of Brazil. AIDS Res. Hum. Retroviruses..

[183] Nyambi P.N., Ville Y., Louwagie J., Bedjabaga I., Glowaczower E., Peeters M. (1996). Mother-to-child transmission of human T-cell lymphotropic virus types I and II (HTLV-I/II) in Gabon: a prospective follow-up of 4 years. J. Acquir. Immune. Defic. Syndr. Hum. Retrovirol..

[184] Takahashi K., Takezaki T., Oki T., Kawakami K., Yashiki S., Fujiyoshi T. (1991). Inhibitory effect of maternal antibody on mother-to-child transmission of human T-lymphotropic virus type I. The Mother-to-Child Transmission Study Group. Int J. Cancer..

[185] Takezaki T., Tajima K., Ito M., Ito S., Kinoshita K., Tachibana K. (1997). Short-term breast-feeding may reduce the risk of vertical transmission of HTLV-I. The Tsushima ATL Study Group. Leukemia.

[186] Van Dyke R.B., Heneine W., Perrin M.E., Rudolph D., Starszak E., Woods T. (1995). Mother-to-child transmission of human T-lymphotropic virus type II. J. Pediatr..

[187] Vitek C.R., Gracia F.I., Giusti R., Fukuda K., Green D.B., Castillo L.C. (1995). Evidence for sexual and mother-to-child transmission of human T lymphotropic virus type II among Guaymi Indians, Panama. J. Infect. Dis..

[188] Hino S., Katamine S., Miyata H., Tsuji Y., Yamabe T., Miyamoto T. (1996). Primary prevention of HTLV-I in Japan, J. Acquir. Immune. Defic. Syndr. Hum. Retrovirol..

[189] Hino S., Sugiyama H., Doi H., Ishimaru T., Yamabe T., Tsuji Y. (1987). Breaking the cycle of HTLV-I transmission via carrier mothers’ milk. Lancet.

[190] Hino S., Katamine S., Kawase K., Miyamoto T., Doi H., Tsuji Y. (1994). Intervention of maternal transmission of HTLV-1 in Nagasaki, Japan, Leukemia.

[191] Bevot A., Hamprecht K., Krägeloh-Mann I., Brosch S., Goelz R., Vollmer B. (2012). Long-term outcome in preterm children with human cytomegalovirus infection transmitted via breast milk. Acta. Paediatr..

[192] Bryant P., Morley C., Garland S., Curtis N. (2002). Cytomegalovirus transmission from breast milk in premature babies: does it matter?. Arch. Dis. Child Fetal Neonatal. Ed..

[193] Capretti M.G., Lanari M., Lazzarotto T., Gabrielli L., Pignatelli S., Corvaglia L. (2009). Very low birth weight infants born to cytomegalovirus-seropositive mothers fed with their mother’s milk: a prospective study. J. Pediatr..

[194] Doctor S., Friedman S., Dunn M.S., Asztalos E.V., Wylie L., Mazzulli T. (2005). Cytomegalovirus transmission to extremely low-birthweight infants through breast milk. Acta. Paediatr..

[195] Dworsky M., Yow M., Stagno S., Pass R.F., Alford C. (1983). Cytomegalovirus infection of breast milk and transmission in infancy. Pediatrics.

[196] Hamprecht K., Witzel S., Maschmann J., Speer C.P., Jahn G. (2000). Transmission of cytomegalovirus infection through breast milk in term and preterm infants. The role of cell free milk whey and milk cells. Adv. Exp. Med. Biol..

[197] Hamprecht K., Maschmann J., Jahn G., Poets C.F., Goelz R. (2008). Cytomegalovirus transmission to preterm infants during lactation. J. Clin. Virol..

[198] Jim W.T., Shu C.H., Chiu N.C., Kao H.A., Hung H.Y., Chang J.H. (2004). Transmission of cytomegalovirus from mothers to preterm infants by breast milk. Pediatr. Infect. Dis. J..

[199] Josephson C.D., Caliendo A.M., Easley K.A., Knezevic A., Shenvi N., Hinkes M.T. (2014). Blood transfusion and breast milk transmission of cytomegalovirus in very low-birth-weight infants: a prospective cohort study. JAMA Pediatr.

[200] Lawrence R.M. (2006). Cytomegalovirus in human breast milk: risk to the premature infant. Breastfeed. Med..

[201] Lombardi G., Garofoli F., Manzoni P., Stronati M. (2012). Breast milk-acquired cytomegalovirus infection in very low birth weight infants. J. Matern. Fetal Neonatal. Med..

[202] Maschmann J., Hamprecht K., Dietz K., Jahn G., Speer C.P. (2001). Cytomegalovirus infection of extremely low-birth weight infants via breast milk. Clin. Infect. Dis..

[203] Mehler K., Oberthuer A., Lang-Roth R., Kribs A. (2014). High rate of symptomatic cytomegalovirus infection in extremely low gestational age preterm infants of 22–24 weeks’ gestation after transmission via breast milk. Neonatology.

[204] Minamishima I., Ueda K., Minematsu T., Minamishima Y., Umemoto M., Take H. (1994). Role of breast milk in acquisition of cytomegalovirus infection. Microbiol. Immunol..

[205] Miron D., Brosilow S., Felszer K., Reich D., Halle D., Wachtel D. (2005). Incidence and clinical manifestations of breast milk-acquired Cytomegalovirus infection in low birth weight infants. J. Perinatol..

[206] Mosca F., Pugni L., Barbi M., Binda S. (2001). Transmission of cytomegalovirus. Lancet.

[207] Musonda K.G., Nyonda M., Filteau S., Kasonka L., Monze M., Gompels U.A. (2016). Increased cytomegalovirus secretion and risks of infant infection by breastfeeding duration from maternal human immunodeficiency virus positive compared to negative mothers in sub - saharan africa. J. Pediatric. Infect. Dis. Soc..

[208] Neuberger P., Hamprecht K., Vochem M., Maschmann J., Speer C.P., Jahn G. (2006). Case-control study of symptoms and neonatal outcome of human milk-transmitted cytomegalovirus infection in premature infants. J. Pediatr..

[209] Resch B. (2012). Breast milk-acquired cytomegalovirus infection: possible long-term sequelae in preterm infants are still in dispute. Acta. Paediatr..

[210] Richardson B.A., John-Stewart G., Atkinson C., Nduati R., Ásbjörnsdóttir K., Boeckh M. (2016). Vertical cytomegalovirus transmission from HIV- infected women randomized to formula - feed or breastfeed their infants. J. Infect. Dis..

[211] Schleiss M.R. (2006). Acquisition of human cytomegalovirus infection in infants via breast milk: natural immunization or cause for concern?. Rev. Med. Virol..

[212] Stagno S., Reynolds D.W., Pass R.F., Alford C.A. (1980). Breast milk and the risk of cytomegalovirus infection. N. Engl. J. Med..

[213] Takahashi R., Tagawa M., Sanjo M., Chiba H., Ito T., Yamada M. (2007). Severe postnatal cytomegalovirus infection in a very premature infant. Neonatology.

[214] Vochem M., Hamprecht K., Jahn G., Speer C.P. (1998). Transmission of cytomegalovirus to preterm infants through breast milk. Pediatr. Infect. Dis. J..

[215] Wu J., Tang Z.Y., Wu Y.X., Li W.R. (1989). Acquired cytomegalovirus infection of breast milk in infancy. Chin. Med. J..

[216] Croly-Labourdette S., Vallet S., Gagneur A., Gremmo-Feger G., Legrand-Quillien M.C., Ansquer H. (2006). [Pilot epidemiologic study of transmission of cytomegalovirus from mother to preterm infant by breastfeeding]. Arch. Pediatr..

[217] Mussi-Pinhata M.M., Yamamoto A.Y., do Carmo Rego M.A., Pinto P.C.G., da Motta M.S.F., Calixto C. (2004). Perinatal or early-postnatal cytomegalovirus infection in preterm infants under 34 weeks gestation born to CMV-seropositive mothers within a high-seroprevalence population. J. Pediatr..

[218] Narvaez-Arzate R.V., Olguin-Mexquitic L., Lima-Rogel V., Noyola D.E., Barrios-Compean L.M., Villegas-Alvarez C. (2013). Cytomegalovirus infection in infants admitted to a neonatal intensive care unit. J. Matern. Fetal Neonatal. Med..

[219] Patel R.M., Shenvi N., Knezevic A., Hinkes M., Bugg G.W., Stowell S.R. (2019). Observational study of cytomegalovirus from breast milk and necrotising enterocolitis. Arch. Dis. Child Fetal Neonatal. Ed..

[220] Romero-Gómez M.P., Cabrera M., Montes-Bueno M.T., Cendejas-Bueno E., Segovia C., Pastrana N. (2015). Evaluation of cytomegalovirus infection in low-birth weight children by breast milk using a real-time polymerase chain reaction assay. J. Med. Virol..

[221] Yasuda A., Kimura H., Hayakawa M., Ohshiro M., Kato Y., Matsuura O. (2003). Evaluation of cytomegalovirus infections transmitted via breast milk in preterm infants with a real-time polymerase chain reaction assay. Pediatrics.

[222] Dunkle L.M., Schmidt R.R., O’Connor D.M. (1979). Neonatal herpes simplex infection possibly acquired via maternal breast milk. Pediatrics.

[223] Sullivan-Bolyai J.Z., Fife K.H., Jacobs R.F., Miller Z., Corey L. (1983). Disseminated neonatal herpes simplex virus type 1 from a maternal breast lesion. Pediatrics.

[224] Quinn P.T., Lofberg J.V. (1978). Maternal herpetic breast infection: another hazard of neonatal herpes simplex. Med. J. Aust..

[225] World Health Organization (2020). https://apps.who.int/iris/handle/10665/332639.

[226] Coronavirus Disease (COVID-19): What parents should know | UNICEF Pacific Islands [Internet] [cited March 29, 2023]. Available from: https://www.unicef.org/pacificislands/stories/coronavirus-disease-covid-19-what-parents-should-know.

[227] CDC (2021). https://www.cdc.gov/breastfeeding/breastfeeding-special-circumstances/maternal-or-infant-illnesses/covid-19-and-breastfeeding.html.

[228] ABM Statement Coronavirus [Internet] [cited March 29, 2023]. Available from: https://www.bfmed.org/abm-statement-coronavirus.

[229] COVID-19: EMBA Position Statement | EMBA [Internet] [cited March 29, 2023]. Available from: https://europeanmilkbanking.com/covid-19-emba-position-statement.

[230] Davanzo R., Moro G., Sandri F., Agosti M., Moretti C., Mosca F. (2020). Breastfeeding and coronavirus disease-2019: ad interim indications of the Italian Society of Neonatology endorsed by the Union of European Neonatal & Perinatal Societies. Matern. Child. Nutr..

[231] Kuhn S., Twele-Montecinos L., MacDonald J., Webster P., Law B. (2011). Case report: probable transmission of vaccine strain of yellow fever virus to an infant via breast milk. CMAJ.

[232] Traiber C., Coelho-Amaral P., Ritter V.R.F., Winge A. (2011). Infant meningoencephalitis caused by yellow fever vaccine virus transmitted via breastmilk. J. Pediatr. (Rio J)..

[233] Boussemart T., Babe P., Sibille G., Neyret C., Berchel C. (2001). Prenatal transmission of dengue: two new cases. J. Perinatol..

[234] Halstead S.B., Lan N.T., Myint T.T., Shwe T.N., Nisalak A., Kalyanarooj S. (2002). Dengue hemorrhagic fever in infants: research opportunities ignored, Emerg. Infect. Dis..

[235] Hinckley A.F., O’Leary D.R., Hayes E.B. (2007). Transmission of West Nile virus through human breast milk seems to be rare. Pediatrics.

[236] Blohm G.M., Lednicky J.A., Márquez M., White S.K., Loeb J.C., Pacheco C.A. (2018). Evidence for mother -to- child transmission of Zika virus through breast milk. Clin. Infect. Dis..

[237] Hemachudha P., Wacharapluesadee S., Buathong R., Petcharat S., Bunprakob S., Ruchiseesarod C. (2019). Lack of transmission of Zika virus infection to breastfed infant. Clin. Med. Insights Case Rep..

[238] Mann T.Z., Haddad L.B., Williams T.R., Hills S.L., Read J.S., Dee D.L. (2018). Breast milk transmission of flaviviruses in the context of zika virus: a systematic review. Paediatr. Perinat. Epidemiol..

[239] Mello A.S., Pascalicchio Bertozzi A.P.A., Rodrigues M.M.D., Gazeta R.E., Moron A.F., Soriano-Arandes A. (2019). Development of secondary microcephaly after delivery: possible consequence of mother - baby transmission of Zika virus in breast milk. Am J. Case Rep..

[240] Sampieri C.L., Montero H. (2019). Breastfeeding in the time of Zika: a systematic literature review. PeerJ.

[241] Besnard M., Lastere S., Teissier A., Cao-Lormeau V., Musso D. (2014). Evidence of perinatal transmission of Zika virus, French Polynesia. Euro. Surveill..

[242] Besnard M., Dub T., Gérardin P. (2017). Outcomes for 2 children after peripartum acquisition of Zika virus infection, French Polynesia. Emerg. Infect. Dis..

[243] Desclaux A., de Lamballerie X., Leparc-Goffart I., Vilain-Parcé A., Coatleven F., Fleury H. (2018). Probable sexually transmitted Zika virus infection in a pregnant woman. N. Engl. J. Med..

[244] Giovanetti M., Goes de Jesus J., Lima de Maia M., Junior J.X., Castro Amarante M.F., Viana P. (2018). Genetic evidence of Zika virus in mother’s breast milk and body fluids of a newborn with severe congenital defects. Clin. Microbiol. Infect..

[245] Rodó C., Suy A., Sulleiro E., Soriano-Arandes A., Maiz N., García-Ruiz I. (2019). Pregnancy outcomes after maternal Zika virus infection in a non-endemic region: prospective cohort study. Clin. Microbiol. Infect..

[246] Tozetto-Mendoza T.R., Avelino-Silva V.I., Fonseca S., Claro I.M., Paula de A.V., Levin A.S. (2019). Zika virus infection among symptomatic patients from two healthcare centers in Sao Paulo State, Brazil: prevalence, clinical characteristics, viral detection in body fluids and serodynamics. Rev. Inst. Med. Trop. Sao Paulo..

[247] Villamil-Gómez W.E., Guijarro E., Castellanos J., Rodríguez-Morales A.J. (2017). Congenital Zika syndrome with prolonged detection of Zika virus RNA. J. Clin. Virol..

[248] (2007). Information NC for B, Pike USNL of M 8600 R, MD B, USA 20894. Article. Arch. Dis. Child..

[249] Mast E.E. (2004). Mother-to-infant hepatitis C virus transmission and breastfeeding. Adv. Exp. Med. Biol..

[250] Polywka S., Schröter M., Feucht H.H., Zöllner B., Laufs R. (1999). Low risk of vertical transmission of hepatitis C virus by breast milk. Clin. Infect. Dis..

[251] Voyer M., Nobre R., Magny J.F. (2001). [Breastfeeding and hepatitis C virus (HCV): the need for a careful appraisal]. Arch. Pediatr..

[252] Dal Molin G., D’Agaro P., Ansaldi F., Ciana G., Fertz C., Alberico S. (2002). Mother-to-infant transmission of hepatitis C virus: rate of infection and assessment of viral load and IgM anti-HCV as risk factors. J. Med. Virol..

[253] Gibb D.M., Goodall R.L., Dunn D.T., Healy M., Neave P., Cafferkey M. (2000). Mother-to-child transmission of hepatitis C virus: evidence for preventable peripartum transmission. Lancet.

[254] Manzini P., Saracco G., Cerchier A., Riva C., Musso A., Ricotti E. (1995). Human immunodeficiency virus infection as risk factor for mother-to-child hepatitis C virus transmission; persistence of anti-hepatitis C virus in children is associated with the mother’s anti-hepatitis C virus immunoblotting pattern. Hepatology.

[255] Moriya T., Sasaki F., Mizui M., Ohno N., Mohri H., Mishiro S. (1995). Transmission of hepatitis C virus from mothers to infants: its frequency and risk factors revisited. Biomed. Pharmacother..

[256] Ohto H., Terazawa S., Sasaki N., Sasaki N., Hino K., Ishiwata C. (1994). Transmission of hepatitis C virus from mothers to infants. The vertical transmission of hepatitis C virus collaborative study group. N. Engl. J. Med..

[257] Townsend C.L., Peckham C.S., Thorne C., Kourtis A.P., Bulterys M. (2012). Human Immunodeficiency Virus type 1 (HIV-1) and Breastfeeding: Science, Research Advances, and Policy [Internet].

[258] Hepatitis B., breastfeeding (1997). Indian Pediatr.

[259] Hill J.B., Sheffield J.S., Kim M.J., Alexander J.M., Sercely B., Wendel G.D. (2002). Risk of hepatitis B transmission in breast-fed infants of chronic hepatitis B carriers. Obstet. Gynecol..

[260] Petrova M., Kamburov V. (2010). Breastfeeding and chronic HBV infection: clinical and social implications. World J. Gastroenterol..

[261] Pirillo M.F., Scarcella P., Andreotti M., Jere H., Buonomo E., Sagno J.B. (2015). Hepatitis B virus mother-to-child transmission among HIV-infected women receiving lamivudine-containing antiretroviral regimens during pregnancy and breastfeeding. J. Viral Hepat..

[262] Wang T., Wang M., Duan G., Chen X., He Y. (2015). Discrepancy in impact of maternal milk on vertical transmission between Hepatitis B virus and Human cytomegalovirus. Int. J. Infect. Dis..

[263] Xiao F., Lan A., Mo W. (2017). Breastfeeding from mothers carrying HBV would not increase the risk of HBV infection in infants after proper immunoprophylaxis. Minerva. Pediatr..

[264] Zhang L., Gui X., Fan J., Wang B., Ji H., Yisilafu R. (2014). Breast feeding and immunoprophylaxis efficacy of mother-to-child transmission of hepatitis B virus. J. Matern. Fetal Neonatal. Med..

[265] Watson J.C., Fleming D.W., Borella A.J., Olcott E.S., Conrad R.E., Baron R.C. (1993). Vertical transmission of hepatitis A resulting in an outbreak in a neonatal intensive care unit. J. Infect. Dis..

[266] (2006). Human Papillomavirus Vaccines. Drugs and Lactation Database (LactMed®) [Internet].

[267] Bower H., Johnson S., Bangura M.S., Kamara A.J., Kamara O., Mansaray S.H. (2016). Effects of mother’s illness and breastfeeding on risk of ebola virus disease in a cohort of very young children. PLOS Negl. Trop. Dis..

[268] Bausch D.G., Towner J.S., Dowell S.F., Kaducu F., Lukwiya M., Sanchez A. (2007). Assessment of the risk of Ebola virus transmission from bodily fluids and fomites. J. Infect. Dis..

[269] Caluwaerts S., Fautsch T., Lagrou D., Moreau M., Modet Camara A., Günther S. (2016). Dilemmas in managing pregnant women with ebola: 2 case reports. Clin. Infect. Dis..

[270] Losonsky G.A., Fishaut J.M., Strussenberg J., Ogra P.L. (1982). Effect of immunization against rubella on lactation products. II. Maternal-neonatal interactions. J. Infect. Dis..

[271] Puchalski Ritchie L.M., van Lettow M., Pham B., Straus S.E., Hosseinipour M.C., Rosenberg N.E. (2019). What interventions are effective in improving uptake and retention of HIV-positive pregnant and breastfeeding women and their infants in prevention of mother to child transmission care programmes in low-income and middle-income countries? a systematic review and meta-analysis. BMJ Open.

[272] Becquet R., Marston M., Dabis F., Moulton L.H., Gray G., Coovadia H.M. (2012). Children who acquire HIV infection perinatally are at higher risk of early death than those acquiring infection through breastmilk: a meta-analysis. PLOS ONE.

[273] Boostani R., Sadeghi R., Sabouri A., Ghabeli-Juibary A. (2018). Human T-lymphotropic virus type I and breastfeeding; systematic review and meta-analysis of the literature. Iran J. Neurol..

[274] Ando Y., Matsumoto Y., Nakano S., Saito K., Kakimoto K., Tanigawa T. (2003). Long-term follow-up study of HTLV-I infection in bottle-fed children born to seropositive mothers. J. Infect..

[275] Hu X., Hu W., Sun X., Chen L., Luo X. (2021). Transmission of cytomegalovirus via breast milk in low birth weight and premature infants: a systematic review and meta-analysis. BMC Pediatr.

[276] Park H.W., Cho M.H., Bae S.H., Lee R., Kim K.S. (2021). Incidence of postnatal CMV infection among breastfed preterm infants: a systematic review and meta -analysis. J. Korean Med. Sci..

[277] Martins-Celini F.P., Yamamoto A.Y., Passos D.M., do Nascimento S.D., Lima E.V., Di Giovanni C.M. (2016). Incidence, risk factors, and morbidity of acquired postnatal cytomegalovirus infection among preterm infants fed maternal milk in a highly seropositive population. Clin. Infect. Dis..

[278] Jim W.T., Shu C.H., Chiu N.C., Chang J.H., Hung H.Y., Peng C.C. (2009). High cytomegalovirus load and prolonged virus excretion in breast milk increase risk for viral acquisition by very low birth weight infants, Pediatr. Infect. Dis J..

[279] Omarsdottir S., Casper C., Zweygberg Wirgart B., Grillner L., Vanpée M. (2007). Transmission of cytomegalovirus to extremely preterm infants through breast milk. Acta. Paediatr..

[280] Buxmann H., Miljak A., Fischer D., Rabenau H.F., Doerr H.W., Schloesser R.L. (2009). Incidence and clinical outcome of cytomegalovirus transmission via breast milk in preterm infants </=31 weeks. Acta. Paediatr..

[281] Yoo H.S., Sung S.I., Jung Y.J., Lee M.S., Han Y.M., Ahn S.Y. (2015). Prevention of cytomegalovirus transmission via breast milk in extremely low birth weight infants. Yonsei. Med. J..

[282] Chiavarini M., Bragetti P., Sensini A., Cenci E., Castronari R., Rossi M.J. (2011). Breastfeeding and transmission of cytomegalovirus to preterm infants. Case report and kinetic of CMV-DNA in breast milk. Ital J. Pediatr..

[283] Hayashi S., Kimura H., Oshiro M., Kato Y., Yasuda A., Suzuki C. (2011). Transmission of cytomegalovirus via breast milk in extremely premature infants. J. Perinatol..

[284] D’Andrea M.A., Spatz D.L. (2019). Maintaining Breastfeeding during severe infant and maternal HSV-1 infection: a case report. J. Hum Lact..

[285] Field S.S. (2016). Fatal neonatal herpes simplex infection likely from unrecognized breast lesions. J. Hum Lact..

[286] Yoshida M., Yamagami N., Tezuka T., Hondo R. (1992). Case report: detection of varicella-zoster virus DNA in maternal breast milk. J. Med. Virol..

[287] Liu W., Wang J., Li W., Zhou Z., Liu S., Rong Z. (2020). Clinical characteristics of 19 neonates born to mothers with COVID-19. Front Med.

[288] Wu Y., Liu C., Dong L., Zhang C., Chen Y., Liu J. (2020). Coronavirus disease 2019 among pregnant Chinese women: case series data on the safety of vaginal birth and breastfeeding. BJOG.

[289] Dong L., Tian J., He S., Zhu C., Wang J., Liu C. (2020). Possible vertical transmission of SARS-CoV-2 from an infected mother to her newborn. JAMA.

[290] Zhu F., Zozaya C., Zhou Q., De Castro C., Shah P.S. (2021). SARS-CoV-2 genome and antibodies in breastmilk: a systematic review and meta-analysis. Arch. Dis. Child Fetal Neonatal. Ed..

[291] Low J.M., Low Y.W., Zhong Y., Lee C.Y.C., Chan M., Ng N.B.H. (2022). Titres and neutralising capacity of SARS-CoV-2-specific antibodies in human milk: a systematic review. Arch. Dis. Child Fetal Neonatal. Ed..

[292] Ribeiro A.F., Guedes B.F., Sulleiman J.M.A.H., de Oliveira F.T.M., de Souza I.O.M., Nogueira J.S. (2021). Neurologic disease after yellow fever vaccination, São Paulo, Brazil, 2017–2018. Emerg Infect. Infect. Dis..

[293] Centers for Disease Control and Prevention (CDC) (2010). Transmission of yellow fever vaccine virus through breast-feeding - Brazil, 2009. MMWR Morb Mortal Wkly Rep.

[294] (2002). Possible West Nile Virus Transmission to an Infant Through Breast-Feeding—Michigan. JAMA.

[295] Centeno-Tablante E., Medina-Rivera M., Finkelstein J.L., Herman H.S., Rayco-Solon P., Garcia-Casal M.N. (2021). Update on the transmission of zika virus through breast milk and breastfeeding: a systematic review of the evidence. Viruses.

[296] de Quental O.B., França E.L., Honório-França A.C., Morais T.C., Daboin B.E.G., Bezerra I.M.P. (2019). Zika virus alters the viscosity and cytokines profile in human colostrum. J. Immunol. Res..

[297] Ruiz-Extremera A., Salmerón J., Torres C., De Rueda P.M., Giménez F., Robles C. (2000). Follow-up of transmission of hepatitis C to babies of human immunodeficiency virus-negative women: the role of breast-feeding in transmission, Pediatr. Infect. Dis J..

[298] Shi Z., Yang Y., Wang H., Ma L., Schreiber A., Li X. (2011). Breastfeeding of newborns by mothers carrying hepatitis B virus: a meta-analysis and systematic review. Arch. Pediatr. Adolesc. Med..

[299] Chibber R.M., Usmani M.A., Al-Sibai M.H. (2004). Should HEV infected mothers breast feed?. Arch. Gynecol. Obstet..

[300] Klein E.B., Byrne T., Cooper L.Z. (1980). Neonatal rubella in a breast-fed infant after postpartum maternal infection. J. Pediatr..

[301] (2006). Drugs and Lactation Database.

[302] Stark A., Cantrell S., Greenberg R.G., Permar S.R., Weimer K.E.D. (2021). Long-term outcomes after postnatal cytomegalovirus infection in low birthweight preterm infants: a systematic review. Pediatr Infect. Dis. J..

[303] Osterholm E.A., Schleiss M.R. (2020). Impact of breast milk-acquired cytomegalovirus infection in premature infants: pathogenesis, prevention, and clinical consequences?. Rev. Med. Virol..

[304] Chen H., Guo J., Wang C., Luo F., Yu X., Zhang W. (2020). Clinical characteristics and intrauterine vertical transmission potential of COVID-19 infection in nine pregnant women: a retrospective review of medical records. Lancet.

[305] Vivanti A.J., Vauloup-Fellous C., Prevot S., Zupan V., Suffee C., Do Cao J. (2020). Transplacental transmission of SARS-CoV-2 infection. Nat. Commun..

[306] Zeng H., Xu C., Fan J., Tang Y., Deng Q., Zhang W. (2020). Antibodies in infants born to mothers with COVID-19 pneumonia. JAMA.

[307] Bode L. (2015). The functional biology of human milk oligosaccharides. Early Hum. Dev..

[308] Bode L., Kuhn L., Kim H.Y., Hsiao L., Nissan C., Sinkala M. (2012). Human milk oligosaccharide concentration and risk of postnatal transmission of HIV through breastfeeding. Am. J. Clin. Nutr..

[309] Etzold S., Bode L. (2014). Glycan-dependent viral infection in infants and the role of human milk oligosaccharides. Curr. Opin. Virol..

[310] Hanisch F.G., Hansman G.S., Morozov V., Kunz C., Schroten H. (2018). Avidity of α-fucose on human milk oligosaccharides and blood group-unrelated oligo/polyfucoses is essential for potent norovirus-binding targets. J. Biol. Chem..

[311] Hester S.N., Chen X., Li M., Monaco M.H., Comstock S.S., Kuhlenschmidt T.B. (2013). Human milk oligosaccharides inhibit rotavirus infectivity in vitro and in acutely infected piglets. Br. J. Nutr..

[312] Koromyslova A., Tripathi S., Morozov V., Schroten H., Hansman S. (2017). Human norovirus inhibition by a human milk oligosaccharide. Virology.

[313] Krammer E.M., Bouckaert J.M.J. (2018). Norovirus devours human milk oligosaccharides rich in α-fucose. J. Biol. Chem..

[314] Laucirica D.R., Triantis V., Schoemaker R., Estes M.K., Ramani S. (2017). Milk oligosaccharides inhibit human rotavirus infectivity in MA104 cells. J. Nutr..

[315] Li M., Monaco M.H., Wang M., Comstock S.S., Kuhlenschmidt T.B., Fahey G.C. (2014). Human milk oligosaccharides shorten rotavirus-induced diarrhea and modulate piglet mucosal immunity and colonic microbiota. ISME J.

[316] Morozov V., Hansman G., Hanisch F.G., Schroten H., Kunz C. (2018). Human milk oligosaccharides as promising antivirals. Mol. Nutr. Food Res..

[317] Morrow A.L., Ruiz-Palacios G.M., Altaye M., Jiang X., Guerrero M.L., Meinzen-Derr J.K. (2004). Human milk oligosaccharide blood group epitopes and innate immune protection against campylobacter and calicivirus diarrhea in breastfed infants. Adv. Exp. Med. Biol..

[318] Newburg D.S. (2009). Neonatal protection by an innate immune system of human milk consisting of oligosaccharides and glycans. J. Anim. Sci..

[319] Newburg D.S., Ruiz-Palacios G.M., Morrow L. (2005). Human milk glycans protect infants against enteric pathogens. Ann. Rev. Nutr..

[320] Pandey R.P., Kim D.H., Woo J., Song J., Jang S.H., Kim J.B. (2018). Broad-spectrum neutralization of avian influenza viruses by sialylated human milk oligosaccharides: in vivo assessment of 3’-sialyllactose against H9N2 in chickens. Sci. Rep..

[321] Ramani S., Stewart C.J., Laucirica D.R., Ajami N.J., Robertson B., Autran C.A. (2018). Human milk oligosaccharides, milk microbiome and infant gut microbiome modulate neonatal rotavirus infection. Nat. Commun..

[322] Schroten H., Hanisch F.G., Hansman G.S. (2016). Human norovirus interactions with histo - blood group antigens and human milk oligosaccharides. J. Virol..

[323] Shang J., Piskarev V.E., Xia M., Huang P., Jiang X., Likhosherstov L.M. (2013). Identifying human milk glycans that inhibit norovirus binding using surface plasmon resonance. Glycobiology.

[324] Konlee M. (1998). Sulfated polysaccharides (chondroitin sulfate and carrageenan) plus glucosamine sulfate are potent inhibitors of HIV. Posit. Health News..

[325] Portelli J., Gordon A., May J.T. (1998). Effect of compounds with antibacterial activities in human milk on respiratory syncytial virus and cytomegalovirus in vitro. J. Med. Microbiol..

[326] Viveros-Rogel M., Soto-Ramirez L., Chaturvedi P., Newburg D.S., Ruiz-Palacios G.M. (2004). Inhibition of HIV-1 infection in vitro by human milk sulfated glycolipids and glycosaminoglycans. Adv. Exp. Med. Biol..

[327] Francese R., Civra A., Donalisio M., Volpi N., Capitani F., Sottemano S. (2020). Anti-Zika virus and anti-Usutu virus activity of human milk and its components. PLOS Negl. Trop. Dis..

[328] Chang R., Ng T.B., Sun W.Z. (2020). Lactoferrin as potential preventative and adjunct treatment for COVID-19. Int J. Antimicrob. Agents..

[329] Giansanti F., Panella G., Leboffe L., Antonini G. (2016). Lactoferrin from milk: nutraceutical and pharmacological properties. Pharmaceuticals (Basel).

[330] Grover M., Giouzeppos O., Schnagl R.D., May J.T. (1997). Effect of human milk prostaglandins and lactoferrin on respiratory syncytial virus and rotavirus. Acta. Paediatr..

[331] Harmsen M.C., Swart P.J., de Béthune M.P., Pauwels R., De Clercq E., The T.H. (1995). Antiviral effects of plasma and milk proteins: lactoferrin shows potent activity against both human immunodeficiency virus and human cytomegalovirus replication in vitro. J. Infect. Dis..

[332] Henrick B.M., Yao X.D., Nasser L., Roozrogousheh A., Rosenthal K.L. (2017). Breastfeeding behaviors and the innate immune system of human milk: working together to protect infants against Inflammation, HIV-1, and other infections. Front. Immunol..

[333] Jenssen H., Hancock R.E.W. (2009). Antimicrobial properties of lactoferrin. Biochimie.

[334] Kanyshkova T.G., Babina S.E., Semenov D.V., Isaeva N., Vlassov A.V., Neustroev K.N. (2003). Multiple enzymic activities of human milk lactoferrin. Eur. J. Biochem..

[335] Kazmi S.H., Naglik J.R., Sweet S.P., Evans R.W., O’Shea S., Banatvala J.E. (2006). Comparison of human immunodeficiency virus type 1-specific inhibitory activities in saliva and other human mucosal fluids. Clin. Vaccine Immunol..

[336] Parrón J.A., Ripollés D., Ramos S.J., Pérez M.D., Semen Z., Rubio P. (2018). Antirotaviral potential of lactoferrin from different origin: effect of thermal and high pressure treatments. Biometals.

[337] Sharma D., Shastri S., Sharma P. (2017). Role of lactoferrin in neonatal care: a systematic review. J. Matern. Fetal Neonatal. Med..

[338] Swart P.J., Kuipers E.M., Smit C., Van Der Strate B.W., Harmsen M.C., Meijer D.K. (1998). Lactoferrin. Antiviral activity of lactoferrin. Adv. Exp. Med. Biol..

[339] Wang H., Ye X., Ng T.B. (2000). First demonstration of an inhibitory activity of milk proteins against human immunodeficiency virus-1 reverse transcriptase and the effect of succinylation. Life Sci.

[340] Yi M., Kaneko S., Yu D.Y., Murakami S. (1997). Hepatitis C virus envelope proteins bind lactoferrin. J. Virol..

[341] Weimer K.E.D., Roark H., Fisher K., Cotten C.M., Kaufman D.A., Bidegain M. (2021). Breast milk and saliva lactoferrin levels and postnatal cytomegalovirus infection. Am. J. Perinatol..

[342] Fan H., Hong B., Luo Y., Peng Q., Wang L., Jin X. (2020). The effect of whey protein on viral infection and replication of SARS-CoV-2 and pangolin coronavirus in vitro. Signal Transduct. Target Ther..

[343] Lai X., Yu Y., Xian W., Ye F., Ju X., Luo Y. (2022). Identified human breast milk compositions effectively inhibit SARS-CoV-2 and variants infection and replication. iScience.

[344] Hamosh M., Peterson J.A., Henderson T.R., Scallan C.D., Kiwan R., Ceriani R.L. (1999). Protective function of human milk: the milk fat globule. Semin. Perinatol..

[345] Kvistgaard A.S., Pallesen L.T., Arias C.F., López S., Petersen T.E., Heegaard C.W. (2004). Inhibitory effects of human and bovine milk constituents on rotavirus infections. J. Dairy Sci..

[346] Newburg D.S., Peterson J.A., Ruiz-Palacios G.M., Matson D.O., Morrow A.L., Shults J. (1998). Role of human-milk lactadherin in protection against symptomatic rotavirus infection. Lancet.

[347] Habte H.H., Kotwal G.J., Lotz Z.E., Tyler M.G., Abrahams M., Rodriques J. (2007). Antiviral activity of purified human breast milk mucin. Neonatology.

[348] Habte H.H., de Beer C., Lotz Z.E., Tyler M.G., Kahn D., Mall A.S. (2008). Inhibition of human immunodeficiency virus type 1 activity by purified human breast milk mucin (MUC1) in an inhibition assay. Neonatology.

[349] Mthembu Y., Lotz Z., Tyler M., de Beer C., Rodrigues J., Schoeman L. (2014). Purified human breast milk MUC1 and MUC4 inhibit human immunodeficiency virus. Neonatology.

[350] Ruvoën-Clouet N., Mas E., Marionneau S., Guillon P., Lombardo D., Le Pendu J. (2006). Bile-salt-stimulated lipase and mucins from milk of «secretor» mothers inhibit the binding of Norwalk virus capsids to their carbohydrate ligands. Biochem. J..

[351] Yolken R.H., Peterson J.A., Vonderfecht S.L., Fouts E.T., Midthun K., Newburg D.S. (1992). Human milk mucin inhibits rotavirus replication and prevents experimental gastroenteritis. J. Clin. Invest..

[352] Pfaender S., Heyden J., Friesland M., Ciesek S., Ejaz A., Steinmann J. (2013). Inactivation of hepatitis C virus infectivity by human breast milk. J. Infect. Dis..

[353] Thormar H., Isaacs C.E., Brown H.R., Barshatzky M.R., Pessolano T. (1987). Inactivation of enveloped viruses and killing of cells by fatty acids and monoglycerides. Antimicrob. Agents Chemother..

[354] Falkler W.A., Diwan A.R., Halstead S.B. (1975). A lipid inhibitor of dengue virus in human colostrum and milk; with a note on the absence of anti-dengue secretory antibody. Arch. Virol..

[355] Civra A., Francese R., Donalisio M., Tonetto P., Coscia A., Sottemano S. (2021). Human colostrum and derived extracellular vesicles prevent infection by human rotavirus and respiratory syncytial virus in vitro. J. Hum. Lact..

[356] Donalisio M., Cirrincione S., Rittà M., Lamberti C., Civra A., Francese R. (2020). Extracellular vesicles in human preterm colostrum inhibit infection by human cytomegalovirus In Vitro. Microorganisms.

[357] Wahl S.M., McNeely T.B., Janoff E.N., Shugars D., Worley P., Tucker C. (1997). Secretory leukocyte protease inhibitor (SLPI) in mucosal fluids inhibits HIV-I. Oral Dis.

[358] Naarding M.A., Ludwig I.S., Groot F., Berkhout B., Geijtenbeek T.B.H., Pollakis G. (2005). Lewis X component in human milk binds DC-SIGN and inhibits HIV-1 transfer to CD4+ T lymphocytes. J. Clin. Invest..

[359] Ng T.B., Lam T.L., Au T.K., Ye X.Y., Wan C.C. (2001). Inhibition of human immunodeficiency virus type 1 reverse transcriptase, protease and integrase by bovine milk proteins. Life Sci.

[360] Weichert S., Koromyslova A., Singh B.K., Hansman S., Jennewein S., Schroten H. (2016). Structural basis for norovirus inhibition by human milk oligosaccharides. J. Virol..

[361] Francese R., Donalisio M., Rittà M., Capitani F., Mantovani V., Maccari F. (2022). Human milk glycosaminoglycans inhibit cytomegalovirus and respiratory syncytial virus infectivity by impairing cell binding. Pediatr. Res..

[362] Moriuchi M., Moriuchi H. (2001). A milk protein lactoferrin enhances human T cell leukemia virus type I and suppresses HIV-1 infection. J. Immunol..

[363] Andersen J.H., Jenssen H., Sandvik K., Gutteberg T.J. (2004). Anti-HSV activity of lactoferrin and lactoferricin is dependent on the presence of heparan sulphate at the cell surface. J. Med. Virol..

[364] Peterson J.A., Patton S., Hamosh M. (1998). Glycoproteins of the human milk fat globule in the protection of the breast-fed infant against infections. Biol. Neonate..

[365] Saeland E., de Jong M.A.W.P., Nabatov A.A., Kalay H., Geijtenbeek T.B.H., van Kooyk Y. (2009). MUC1 in human milk blocks transmission of human immunodeficiency virus from dendritic cells to T cells. Mol. Immunol..

[366] Civra A., Leoni V., Caccia C., Sottemano S., Tonetto P., Coscia A. (2019). Antiviral oxysterols are present in human milk at diverse stages of lactation. J. Steroid. Biochem. Mol. Biol..

[367] Fouda G.G., Jaeger F.H., Amos J.D., Ho C., Kunz E.L., Anasti K. (2013). Tenascin-C is an innate broad-spectrum, HIV-1-neutralizing protein in breast milk. Proc. Natl. Acad. Sci. USA..

[368] Mangan R.J., Stamper L., Ohashi T., Eudailey J.A., Go E.P., Jaeger F.H. (2019). Determinants of Tenascin-C and HIV-1 envelope binding and neutralization. Mucosal. Immunol..

[369] Mansour R.G., Stamper L., Jaeger F., McGuire E., Fouda G., Amos J. (2016). The presence and anti -HIV-1 function of tenascin C in breast milk and genital fluids. PLOS ONE.

[370] Pfaender S., Vielle N.J., Ebert N., Steinmann E., Alves M.P., Thiel V. (2017). Inactivation of Zika virus in human breast milk by prolonged storage or pasteurization. Virus Res.

[371] Näslund T.I., Paquin-Proulx D., Paredes P.T., Vallhov H., Sandberg J.K., Gabrielsson S. (2014). Exosomes from breast milk inhibit HIV-1 infection of dendritic cells and subsequent viral transfer to CD4+ T cells. AIDS.

[372] Hosea Blewett H.J., Cicalo M.C., Holland C.D., Field C.J. (2008). The immunological components of human milk. Adv. Food Nutr. Res..

[373] Morniroli D., Consales A., Crippa B.L., Vizzari G., Ceroni F., Cerasani J. (2021). The antiviral properties of human milk: a multitude of defence tools from mother nature. Nutrients.

[374] Gila-Diaz A., Arribas S.M., Algara A., Martín-Cabrejas M.A., López de Pablo A.L., Sáenz de Pipaón M. (2019). A review of bioactive factors in human breastmilk: a focus on prematurity. Nutrients.

[375] Juncker H.G., Romijn M., Loth V.N., Ruhé E.J.M., Bakker S., Kleinendorst S. (2021). Antibodies against Sars-Cov-2 in human milk: milk conversion rates in the Netherlands. J. Hum. Lact..

[376] Collier A.R.Y., McMahan K., Yu J., Tostanoski L.H., Aguayo R., Ansel J. (2021). Immunogenicity of COVID-19 mRNA vaccines in pregnant and lactating women. JAMA.

[377] Gray K.J., Bordt E.A., Atyeo C., Deriso E., Akinwunmi B., Young N. (2021). Coronavirus disease 2019 vaccine response in pregnant and lactating women: a cohort study. Am. J. Obstet Gynecol..

[378] Perl S.H., Uzan-Yulzari A., Klainer H., Asiskovich L., Youngster M., Rinott E. (2021). SARS-CoV-2- specific antibodies in breast milk after COVID-19 vaccination of breastfeeding women. JAMA.

[379] Young B.E., Seppo A.E., Diaz N., Rosen-Carole C., Nowak-Wegrzyn A., Cruz Vasquez J.M. (2022). Association of human milk antibody induction, persistence, and neutralizing capacity with Sars-Cov-2 infection vs mrna vaccination. JAMA Pediatr.

[380] Sprenger N., Tytgat H.L.P., Binia A., Austin S., Singhal A. (2022). Biology of human milk oligosaccharides: from basic science to clinical evidence. J. Hum. Nutr. Diet..

[381] Wang J., Chen M.S., Wang R.S., Hu J.Q., Liu S., Wang Y.Y.F. (2022). Current advances in structure - function relationships and dose - dependent effects of human milk oligosaccharides. J. Agric. Food Chem..

[382] Coppa G.V., Gabrielli O., Buzzega D., Zampini L., Galeazzi T., Maccari F. (2011). Composition and structure elucidation of human milk glycosaminoglycans. Glycobiology.

[383] Coppa G.V., Gabrielli O., Bertino E., Zampini L., Galeazzi T., Padella L. (2013). Human milk glycosaminoglycans: the state of the art and future perspectives. Ital J. Pediatr..

[384] Zhang B., Li L.Q., Liu F., Wu J.Y. (2022). Human milk oligosaccharides and infant gut microbiota: molecular structures, utilization strategies and immune function. Carbohydr. Polym..

[385] Maccari F., Mantovani V., Gabrielli O., Carlucci A., Zampini L., Galeazzi T. (2016). Metabolic fate of milk glycosaminoglycans in breastfed and formula fed newborns. Glycoconj. J..

[386] Chutipongtanate S., Morrow A.L., Newburg D.S. (2022). Human milk oligosaccharides: potential applications in Covid-19. Biomedicines.

[387] Kaplan M., Şahutoğlu A.S., Sarıtaş S., Duman H., Arslan A., Pekdemir B. (2022). Role of milk glycome in prevention, treatment, and recovery of COVID-19. Front Nutr.

[388] Briana D.D., Papadopoulou A., Syridou G., Marchisio E., Kapsabeli E., Daskalaki A. (2022). Early human milk lactoferrin during SARS-CoV-2 infection. J. Matern. Fetal. Neonatal. Med..

[389] Andersen J.H., Osbakk S.A., Vorland L.H., Traavik T., Gutteberg T.J. (2001). Lactoferrin and cyclic lactoferricin inhibit the entry of human cytomegalovirus into human fibroblasts. Antiviral. Res..

[390] Andersen J.H., Jenssen H., Gutteberg T.J. (2003). Lactoferrin and lactoferricin inhibit Herpes simplex 1 and 2 infection and exhibit synergy when combined with acyclovir. Antiviral. Res..

[391] Ohradanova-Repic A., Skrabana R., Gebetsberger L., Tajti G., Baráth P., Ondrovičová G. (2022). Blockade of TMPRSS2-mediated priming of SARS-CoV-2 by lactoferricin. Front Immunol.

[392] Wang W.Y., Wong J.H., Ip D.T.M., Wan D.C.C., Cheung R.C., Ng T.B. (2016). Bovine lactoferrampin, human lactoferricin, and lactoferrin 1-11 inhibit nuclear translocation of HIV integrase. Appl. Biochem. Biotechnol..

[393] Jenssen H., Sandvik K., Andersen J.H., Hancock R.E.W., Gutteberg T.J. (2008). Inhibition of HSV cell-to-cell spread by lactoferrin and lactoferricin. Antiviral. Res..

[394] Zempleni J., Aguilar-Lozano A., Sadri M., Sukreet S., Manca S., Wu D. (2017). Biological activities of extracellular vesicles and their cargos from bovine and human milk in humans and implications for infants. J. Nutr..

[395] Donalisio M., Rittà M., Tonetto P., Civra A., Coscia A., Giribaldi M. (2018). Anti- cytomegalovirus activity in human milk and colostrum from mothers of preterm infants. J. Pediatr. Gastroenterol. Nutr..

[396] Luketic L., Delanghe J., Sobol P.T., Yang P., Frotten E., Mossman K.L. (2007). Antigen presentation by exosomes released from peptide-pulsed dendritic cells is not suppressed by the presence of active CTL. J. Immunol..

[397] Manca S., Upadhyaya B., Mutai E., Desaulniers A.T., Cederberg R.A., White B.R. (2018). Milk exosomes are bioavailable and distinct microRNA cargos have unique tissue distribution patterns. Sci. Rep..

[398] Mall A.S., Habte H., Mthembu Y., Peacocke J., de Beer C. (2017). Mucus and mucins: do they have a role in the inhibition of the human immunodeficiency virus?. Virol. J..

[399] Gardner A.S., Rahman I.A., Lai C.T., Hepworth A., Trengove N., Hartmann P.E. (2017). Changes in fatty acid composition of human milk in response to cold - like symptoms in the lactating mother and infant. Nutrients.

[400] Mabaya L., Matarira H.T., Tanyanyiwa D.M., Musarurwa C., Mukwembi J., Mudluli T.E. (2022). Polyunsaturated fatty acid composition in breast milk plasma of HIV-infected and uninfected mothers in relation to infant clinical outcomes. Nutr. Metab. Insights..

[401] Welsh J.K., Arsenakis M., Coelen R.J., May J.T. (1979). Effect of antiviral lipids, heat, and freezing on the activity of viruses in human milk. J. Infect. Dis..

[402] Goldblum R.M., Dill C.W., Albrecht T.B., Alford E.S., Garza C., Goldman A.S. (1984). Rapid high-temperature treatment of human milk. J. Pediatr..

[403] Hamprecht K., Maschmann J., Müller D., Dietz K., Besenthal I., Goelz R. (2004). Cytomegalovirus (CMV) inactivation in breast milk: reassessment of pasteurization and freeze-thawing. Pediatr. Res..

[404] Curtis N., Chau L., Garland S., Tabrizi S., Alexander R., Morley C.J. (2005). Cytomegalovirus remains viable in naturally infected breast milk despite being frozen for 10 days. Arch. Dis. Child Fetal Neonatal. Ed..

[405] Maschmann J., Hamprecht K., Weissbrich B., Dietz K., Jahn G., Speer C.P. (2006). Freeze-thawing of breast milk does not prevent cytomegalovirus transmission to a preterm infant. Arch. Dis. Child Fetal Neonatal. Ed..

[406] Sam S.S., Ingersoll J., Racsa L.D., Caliendo A.M., Racsa P.N., Igwe D. (2018). Long-term stability of CMV DNA in human breast milk. J. J. Clin. Virol..

[407] Donalisio M., Rittà M., Francese R., Civra A., Tonetto P., Coscia A. (2018). High temperature - short time pasteurization has a lower impact on the antiviral properties of human milk than holder pasteurization. Front Pediatr.

[408] Hosseini M., Esmaili H.A., Abdoli Oskouei S., Gojazadeh M., MokariYamchi Z., Layegh V. (2016). Evaluation of the freeze - thawing method in reducing viral load of cytomegalovirus in breast milk of mothers of preterm infants. Breastfeed. Med..

[409] Balcells C., Botet F., Gayete S., Marcos M.Á., Dorronsoro I., de Alba C. (2016). Vertically transmitted cytomegalovirus infection in newborn preterm infants. J. Perinat. Med..

[410] Volder C., Work B.J., Hoegh S.V., Eckhardt M.C., Zachariassen G. (2021). Transmission of cytomegalovirus in fresh and freeze-thawed mother’s own milk to very preterm infants: a cohort study. J. Perinatol..

[411] Friis H., Andersen H.K. (1982). Rate of inactivation of cytomegalovirus in raw banked milk during storage at -20 degrees C and pasteurisation. Br. Med. J. (Clin. Res. Ed)..

[412] Wakabayashi H., Mizuno K., Kohda C., Negoro T., Maekawa C., Sawato S. (2012). Low HCMV DNA copies can establish infection and result in significant symptoms in extremely preterm infants: a prospective study. Am. J. Perinatol..

[413] Work B.J., Volder C., Fenger-Gron J., Zachariassen G. (2022). Cytomegalovirus transmitted from mother’s own milk to a growth - restricted extremely preterm infant: a case report. Adv. Neonatal. Care..

[414] Mikawa T., Mizuno K., Tanaka K., Kohda C., Ishii Y., Yamamoto K. (2019). Microwave treatment of breast milk for prevention of cytomegalovirus infection. Pediatr. Int..

[415] Yoshida Y., Azuma M., Furukawa K., Mizuno K., Den H., Kamiya T. (2022). Microwave heating of human milk with direct temperature monitoring. J. Hum. Lact..

[416] ben-Shoshan M., Mandel D., Lubetzky R., Dollberg S., Mimouni F.B. (2016). Eradication of cytomegalovirus from human milk by microwave Irradiation: a pilot study. Breastfeed. Med..

[417] Lloyd M.L., Hod N., Jayaraman J., Marchant E.A., Christen L., Chiang P. (2016). Inactivation of cytomegalovirus in breast milk using ultraviolet -C irradiation: opportunities for a new treatment option in breast milk banking. PLOS One.

[418] Terpstra F.G., Rechtman D.J., Lee M.L., Hoeij K.V., Berg H., Van Engelenberg F.A.C. (2007). Antimicrobial and antiviral effect of high-temperature short-time (HTST) pasteurization applied to human milk. Breastfeed. Med..

[419] Klotz D., Schreiner M., Falcone V., Jonas D., Kunze M., Weber A. (2018). High- temperature short - time treatment of human milk for bacterial count reduction. Front Pediatr.

[420] Bapistella S., Hamprecht K., Thomas W., Speer C.P., Dietz K., Maschmann J. (2019). Short-term pasteurization of breast milk to prevent postnatal cytomegalovirus transmission in very preterm infants. Clin. Infect. Dis..

[421] Maschmann J., Müller D., Lazar K., Goelz R., Hamprecht K. (2019). New short-term heat inactivation method of cytomegalovirus (CMV) in breast milk: impact on CMV inactivation, CMV antibodies and enzyme activities. Arch. Dis. Child Fetal Neonatal. Ed..

[422] Gayà A., Rittà M., Lembo D., Tonetto P., Cresi F., Sottemano S. (2021). Analysis of thermal sensitivity of human cytomegalovirus assayed in the conventional conditions of a human milk bank. Front Pediatr.

[423] Orloff S.L., Wallingford J.C., McDougal J.S. (1993). Inactivation of human immunodeficiency virus type I in human milk: effects of intrinsic factors in human milk and of pasteurization. J. Hum. Lact..

[424] Jeffery B.S., Webber L., Mokhondo K.R., Erasmus D. (2001). Determination of the effectiveness of inactivation of human immunodeficiency virus by Pretoria pasteurization. J. Trop. Pediatr..

[425] Israel-Ballard K., Chantry C., Dewey K., Lönnerdal B., Sheppard H., Donovan R. (2005). Viral, nutritional, and bacterial safety of flash-heated and pretoria-pasteurized breast milk to prevent mother-to-child transmission of HIV in resource-poor countries: a pilot study. J. Acquir. Immune. Defic. Syndr..

[426] Israel-Ballard K., Donovan R., Chantry C., Coutsoudis A., Sheppard H., Sibeko L. (2007). Flash-heat inactivation of HIV-1 in human milk: a potential method to reduce postnatal transmission in developing countries. J. Acquir. Immune Defic. Syndr..

[427] Hoque S.A., Hoshino H., Anwar K.S., Tanaka A., Shinagawa M., Hayakawa Y. (2013). Transient heating of expressed breast milk up to 65°C inactivates HIV-1 in milk: a simple, rapid, and cost-effective method to prevent postnatal transmission. J. Med. Virol..

[428] Ando Y., Nakano S., Saito K., Shimamoto I., Ichijo M., Toyama T. (1986). Prevention of HTLV-I transmission through the breast milk by a freeze-thawing process. Jpn. J. Cancer Res..

[429] Ando Y., Kakimoto K., Tanigawa T., Furuki K., Saito K., Nakano S. (1989). Effect of freeze-thawing breast milk on vertical HTLV-I transmission from seropositive mothers to children. Jpn. J. Cancer Res..

[430] Ando Y., Ekuni Y., Matsumoto Y., Nakano S., Saito K., Kakimoto K. (2004). Long-term serological outcome of infants who received frozen-thawed milk from human T-lymphotropic virus type-I positive mothers. J. Obstet. Gynaecol. Res..

[431] Hamilton Spence E., Huff M., Shattuck K., Vickers A., Yun N., Paessler S. (2017). Ebola Virus and marburg virus in human milk are inactivated by holder pasteurization. J. Hum. Lact..

[432] Elimination of Ebola Virus and Marburg Virus in human Milk Through Holder Pasteurization | Pediatrics | American Academy of Pediatrics [Internet] [cited March 15, 2023]. Available from: =https://publications.aap.org/pediatrics/article/141/1_MeetingAbstract/288/1776/Elimination-of-Ebola-Virus-and-Marburg-Virus-in?autologincheck=redirected.

[433] Donalisio M., Cagno V., Vallino M., Moro G.E., Arslanoglu S., Tonetto P. (2014). Inactivation of high-risk human papillomaviruses by Holder pasteurization: implications for donor human milk banking. J. Perinat. Med..

[434] de Oliveira P.R., Yamamoto A.Y., de Souza C.B.S., de Araújo N.M., de Andrade Gomes S., Heck A.R. (2009). Hepatitis B viral markers in banked human milk before and after Holder pasteurization. J. Clin. Virol..

[435] Walker G.J., Clifford V., Bansal N., Stella A.O., Turville S., Stelzer-Braid S. (2020). SARS-CoV-2 in human milk is inactivated by Holder pasteurisation but not cold storage. J. Paediatr. Child Health..

[436] Unger S., Christie-Holmes N., Guvenc F., Budylowski P., Mubareka S., Gray-Owen S.D. (2020). Holder pasteurization of donated human milk is effective in inactivating SARS-CoV-2. CMAJ.

[437] Conzelmann C., Groß R., Meister T.L., Todt D., Krawczyk A., Dittmer U. (2021). Pasteurization inactivates SARS-CoV-2-spiked breast milk. Pediatrics.

[438] Pitino M.A., O’Connor D.L., McGeer A.J., Unger S. (2021). The impact of thermal pasteurization on viral load and detectable live viruses in human milk and other matrices: a rapid review. Appl. Physiol. Nutr. Metab..

[439] Deodhar L., Joshi S. (1991). Microbiological study of breast milk with special reference to its storage in milk bank. J. Postgrad. Med..

[440] Jeffery B.S., Soma-Pillay P., Makin J., Moolman G. (2003). The effect of pretoria pasteurization on bacterial contamination of hand-expressed human breastmilk. J. Trop. Pediatr..

[441] Brüssow H., Benitez O., Uribe F., Sidoti J., Rosa K., Cravioto A. (1993). Rotavirus-inhibitory activity in serial milk samples from Mexican women and rotavirus infections in their children during their first year of life. J. Clin. Microbiol..

[442] Giles M., Mijch A. (2005). Breast milk pasteurisation in developed countries to reduce HIV transmission. Do the benefits outweigh the risks?. Infect. Dis. Obstet. Gynecol..

[443] Chantry C.J., Israel-Ballard K., Moldoveanu Z., Peerson J., Coutsoudis A., Sibeko L. (2009). Effect of flash-heat treatment on immunoglobulins in breast milk. J. Acquir. Immune. Defic. Syndr..

[444] Verd S., López E. (2012). Management of chickenpox with frozen mother’s milk. J. Altern. Complement Med..

[445] Conzelmann C., Zou M., Groß R., Harms M., Röcker A., Riedel C.U. (2019). Storage- dependent generation of potent anti - zikv activity in human breast milk. Viruses.

[446] Perez S.E., Luna Centeno L.D., Cheng W.A., Marentes Ruiz C.J., Lee Y., Congrave-Wilson Z. (2022). Human milk sars - cov -2 antibodies up to 6 months after vaccination. Pediatrics.

[447] van Keulen B.J., Romijn M., Bondt A., Dingess K.A., Kontopodi E., van der Straten K. (2021). Human milk from previously Covid-19- infected mothers: the effect of pasteurization on specific antibodies and neutralization capacity. Nutrients.

[448] Kothari A., Pitino M.A., Unger S., Perreault V., Doyen A., Pouliot Y. (2022). Preservation of anti -cytomegalovirus activity in human milk following high - pressure processing compared to holder pasteurization. Front Nutr.

