# Supplementary material for: Viruses and Human Milk: Transmission or Protection?
Source: Adv Nutr. 2023 Aug 20;14(6):1389–415. doi: 10.1016/j.advnut.2023.08.007 (PMC10721544; doi:10.1016/j.advnut.2023.08.007)
Supplement: Multimedia component 1 [file mmc1.docx]

**Viruses and human milk: transmission or protection?**

Rachele Francese et al.

**Supplementary Materials**

**Search Strings**

[**Viruses detected in human milk** 2](#_Toc117260070)

[PubMed 2](#_Toc117260071)

[Embase 3](#_Toc117260072)

[**Virus transmission through breastfeeding** 6](#_Toc117260073)

[PubMed 6](#_Toc117260074)

[Embase 7](#_Toc117260075)

[**Human milk antiviral activity** 10](#_Toc117260076)

[PubMed 10](#_Toc117260077)

[Embase 12](#_Toc117260078)

[**Human milk pasteurization and storage methods** 16](#_Toc117260079)

[PubMed 16](#_Toc117260080)

[Embase 17](#_Toc117260081)

## **Viruses detected in Human Milk**

## PubMed

#1 “Milk, Human”[Mesh]

#2 "Milk Banks"[Mesh]

#3 “human milk”[tiab] OR “milk bank*”[tiab] OR milkbank*[tiab] OR “donor milk”[tiab] OR “woman milk”[tiab] OR “woman’s milk”[tiab] OR “women milk”[tiab] OR “women’s milk”[tiab]

#4 “Breast Feeding”[Mesh]

#5 “Lactation”[Mesh]

#6 “breast milk”[tiab] OR breastmilk[tiab] OR “breast-feed*”[tiab] OR breastfeed*[tiab] OR breast-fed[tiab] OR breastfed[tiab] OR lactation*[tiab] OR lactating*[tiab] OR “mothers milk”[tiab] OR “mother milk”[tiab] OR “maternal milk”[tiab] OR “nursing mother*”[tiab]

#7 #4 OR #5 OR #6

#8 “Animals”[Mesh]

#9 “Humans”[Mesh]

#10 #8 NOT #9

#11 #7 NOT #10

**#12 #1 OR #2 OR #3 OR #11**

#13 "Viruses"[Mesh]

#14 "Virus Diseases"[Mesh]

#15 viruses[title] OR “virus diseases”[title] OR “viral diseases”[title] OR “virus infections”[title] OR “viral infections”[title]

#16 retrovir*[tiab] OR leukovir*[tiab] OR oncovir*[tiab] OR deltaretrovir*[tiab] OR lentivir*[tiab] OR HIV[tiab] OR HIVs[tiab] OR “human immunodeficiency vir*”[tiab] OR “acquired immunodeficiency syndrome”[tiab] OR AIDS[tiab] OR HTLV[tiab] OR “T lymphotropic vir*”[tiab] OR “T-cell leukemia-lymphoma vir*”[tiab] OR “T-cell leukemia vir*”[tiab] OR “mammary tumor vir*”[tiab] OR HMTV[tiab] OR H-MTV[tiab]

#17 herpesvir*[tiab] OR alphaherpesvir*[tiab] OR simplexvir*[tiab] OR varicellovir*[tiab] OR betaherpesvir*[tiab] OR roseolovir*[tiab] OR rhadinovir*[tiab] OR gammaherpesvir*[tiab] OR lymphocryptovir*[tiab] OR cytomegalovir*[tiab] OR “cytomegalic vir*”[tiab] OR “salivary gland vir*”[tiab] OR CMV[tiab] OR HCMV[tiab] OR “inclusion disease*”[tiab] OR herpes[tiab] OR herpet*[tiab] OR “HHV-5”[tiab] OR HHV5[tiab] OR “HHV-1”[tiab] OR HHV1[tiab] OR “HSV-1”[tiab] OR HSV1[tiab] OR “HHV-2”[tiab] OR HHV2[tiab] OR “HSV-2”[tiab] OR HSV2[tiab] OR “HHV-3”[tiab] OR HHV3[tiab] OR “HSV-3”[tiab] OR HSV3[tiab] OR varicella*[tiab] OR zoster[tiab] OR shingles[tiab] OR “VZ vir*”[tiab] OR VZV[tiab] OR chickenpox[tiab] OR “human B-lymphotropic vir*”[tiab] OR “HHV-6*”[tiab] OR HHV6*[tiab] OR HBLV[tiab] OR roseola[tiab] OR “exanthema subitum”[tiab] OR “HHV-7”[tiab] OR HHV7[tiab] OR “HHV-8”[tiab] OR HHV8[tiab] OR KSHV[tiab] OR “Kaposi*”[tiab] OR “HHV-4”[tiab] OR HHV4[tiab] OR “Epstein-Barr”[tiab] OR “E-B virus*”[tiab] OR EBV[tiab] OR burkitt*[tiab] OR mononucleo*[tiab] OR leukopla*[tiab]

#18 "SARS-CoV-2 variants"[Supplementary Concept]

#19 coronavir*[tiab] OR corona-vir*[tiab] OR alphacoronavir*[tiab] OR betacoronavir*[tiab] OR SARS[tiab] OR MERS[tiab] OR SARS2[tiab] OR “SARS-COV-2”[tiab] OR COVID*[tiab] OR 2019nCoV[tiab] OR "2019-nCoV"[tiab] OR "HCoV-19"[tiab] OR “severe acute respiratory syndrome”[tiab] OR “Middle East respiratory syndrome”[tiab]

#20 flavivir*[tiab] OR arbovir*[tiab] OR “yellow fever”[tiab] OR YFV[tiab] OR dengue[tiab] OR DENV[tiab] OR “breakbone fever”[tiab] OR “West Nile”[tiab] OR WNV[tiab] OR “tick borne”[tiab] OR “Japanese encephalitis”[tiab] OR “Saint Louis”[tiab] OR “St Louis”[tiab] OR zika[tiab] OR zikV[tiab] OR hepacivir*[tiab] OR “hepatitis C”[tiab]

#21 hepadnavir*[tiab] OR orthohepadnavir*[tiab] OR HBV[tiab] OR “hepatitis B”[tiab]

#22 picornavir*[tiab] OR hepatovir*[tiab] OR “hepatitis A”[tiab] OR HAV[tiab] OR enterovir*[tiab] OR echovir*[tiab] OR coxsackie*[tiab] OR CVB3[tiab] OR cardiovir*[tiab] OR herpangin*[tiab]

#23 hepevir*[tiab] OR HEV[tiab] OR “hepatitis E”[tiab]

#24 papillomavir*[tiab] OR alphapapillomavir*[tiab] OR betapapillomavir*[tiab] OR gammapapillomavir*[tiab] OR mupapillomavir*[tiab] OR HPV[tiab] OR wart[tiab]

#25 filovir*[tiab] OR ebola*[tiab] OR EBOV[tiab] OR marburg*[tiab] OR MARV[tiab]

#26 togavir*[tiab] OR alphavir*[tiab] OR “alpha vir*”[tiab] OR chikungunya[tiab] OR CHIKV[tiab] OR “O'nyong-nyong”[tiab] OR “nyong nyong”[tiab] OR “barmah forest”[tiab] OR “ross river”[tiab] OR Sindbis[tiab]

#27 hantavir* OR orthohantavir*[tiab] OR hantaan*[tiab] OR “Andes vir*”[tiab] OR ADNV[tiab] OR puumala[tiab] OR “Seoul vir*”[tiab] OR SEOV[tiab] OR “sin nombre”[tiab] OR SNV[tiab]

#28 anellovir*[tiab] OR alphatorquevir*[tiab] OR “torque teno”[tiab] OR “TT vir*”[tiab] OR TTV[tiab] OR “Transfusion Transmitted vir*”[tiab] OR “SEN vir*”[tiab] OR SENV[tiab] OR SEN-V[tiab]

#29 matonavir*[tiab] OR rubivir*[tiab] OR rubella[tiab] OR “German measles”[tiab] OR RuV[tiab]

**#30 #13 OR #14 OR #15 OR #16 OR #17 OR #18 OR #19 OR #20 OR #21 OR #22 OR #23 OR #24 OR #25 OR #26 OR #27 OR #28 OR #29**

#31 “Milk, Human/virology”[Mesh]

#32 “Milk, Human/microbiology”[Mesh]

#33 "Viruses/isolation and purification"[Mesh]

#34 "Antigens, Viral/isolation and purification"[Mesh]

#35 “Virus Shedding”[Mesh]

#36 “Viral Load”[Mesh]

#37 “DNA, Viral/analysis”[Mesh]

#38 “RNA, Viral/analysis”[Mesh]

#39 "In Vitro Techniques"[Mesh]

#40 "Polymerase Chain Reaction"[Mesh]

#41 “Virus Cultivation”[Mesh]

#42 isolat*[tiab] OR detect*[tiab] OR identif*[tiab] OR found[tiab] OR localised[tiab] OR localized[tiab] OR presence[tiab] OR level[tiab] OR levels[tiab] OR shed[tiab] OR shedding[tiab] OR concentrat*[tiab] OR load[tiab] OR loads[tiab] OR “in vitro”[tiab] OR “polymerase chain reaction”[tiab] OR PCR[tiab] OR culture*[tiab]

**#43 #31 OR #32 OR #33 OR #34 OR #35 OR #36 OR #37 OR #38 OR #39 OR #40 OR #41 OR #42**

#44 #12 AND #30 AND #43

## Embase

#1 'milk bank'/exp

#2 'human milk':ti,ab,kw OR 'milk bank*':ti,ab,kw OR milkbank*:ti,ab,kw OR ‘donor milk’:ti,ab,kw OR ‘woman milk’:ti,ab,kw OR ‘woman s milk’:ti,ab,kw OR ‘women milk’:ti,ab,kw OR ‘women s milk’:ti,ab,kw

#3 'breast milk'/exp

#4 'breast feeding'/exp

#5 'lactation'/exp

#6 'breast milk':ti,ab,kw OR breastmilk:ti,ab,kw OR 'breast-feed*':ti,ab,kw OR 'breast-fed':ti,ab,kw OR breastfed:ti,ab,kw OR lactation*:ti,ab,kw OR lactating:ti,ab,kw OR 'mothers milk':ti,ab,kw OR 'mother milk':ti,ab,kw OR 'maternal milk':ti,ab,kw OR 'nursing mother*':ti,ab,kw

#7 #3 OR #4 OR #5 OR #6

#8 'animal'/de

#9 'nonhuman'/de

#10 'animal experiment'/exp

#11 'human'/de

#12 #8 OR #9 OR #10

#13 #12 NOT #11

#14 #7 NOT #13

**#15 #1 OR #2 OR #14**

#16 'virus'/exp

#17 'virus infection'/exp

#18 viruses:ti OR ‘virus diseases’:ti OR ‘viral diseases’:ti OR ‘virus infections’:ti OR ‘viral infections’:ti

#19 retrovir*:ti,ab,kw OR leukovir*:ti,ab,kw OR oncovir*:ti,ab,kw OR deltaretrovir*:ti,ab,kw OR lentivir*:ti,ab,kw OR HIV:ti,ab,kw OR HIVs:ti,ab,kw OR ‘human immunodeficiency vir*’:ti,ab,kw OR ‘acquired immunodeficiency syndrome’:ti,ab,kw OR AIDS:ti,ab,kw OR HTLV:ti,ab,kw OR ‘T lymphotropic vir*’:ti,ab,kw OR ‘T-cell leukemia-lymphoma vir*’:ti,ab,kw OR ‘T-cell leukemia vir*’:ti,ab,kw OR ‘mammary tumor vir*’:ti,ab,kw OR HMTV:ti,ab,kw OR H-MTV:ti,ab,kw

#20 herpesvir*:ti,ab,kw OR alphaherpesvir*:ti,ab,kw OR simplexvir*:ti,ab,kw OR varicellovir*:ti,ab,kw OR betaherpesvir*:ti,ab,kw OR roseolovir*:ti,ab,kw OR rhadinovir*:ti,ab,kw OR gammaherpesvir*:ti,ab,kw OR lymphocryptovir*:ti,ab,kw OR cytomegalovir*:ti,ab,kw OR ‘cytomegalic vir*’:ti,ab,kw OR ‘salivary gland vir*’:ti,ab,kw OR CMV:ti,ab,kw OR HCMV:ti,ab,kw OR ‘inclusion disease*’:ti,ab,kw OR herpes:ti,ab,kw OR herpet*:ti,ab,kw OR ‘HHV-5’:ti,ab,kw OR HHV5:ti,ab,kw OR ‘HHV-1’:ti,ab,kw OR HHV1:ti,ab,kw OR ‘HSV-1’:ti,ab,kw OR HSV1:ti,ab,kw OR ‘HHV-2’:ti,ab,kw OR HHV2:ti,ab,kw OR ‘HSV-2’:ti,ab,kw OR HSV2:ti,ab,kw OR ‘HHV-3’:ti,ab,kw OR HHV3:ti,ab,kw OR ‘HSV-3’:ti,ab,kw OR HSV3:ti,ab,kw OR varicella*:ti,ab,kw OR zoster:ti,ab,kw OR shingles:ti,ab,kw OR ‘VZ vir*’:ti,ab,kw OR VZV:ti,ab,kw OR chickenpox:ti,ab,kw OR ‘human B-lymphotropic vir*’:ti,ab,kw OR ‘HHV-6*’:ti,ab,kw OR HHV6*:ti,ab,kw OR HBLV:ti,ab,kw OR roseola:ti,ab,kw OR ‘exanthema subitum’:ti,ab,kw OR ‘HHV-7’:ti,ab,kw OR HHV7:ti,ab,kw OR ‘HHV-8’:ti,ab,kw OR HHV8:ti,ab,kw OR KSHV:ti,ab,kw OR ‘Kaposi*’:ti,ab,kw OR ‘HHV-4’:ti,ab,kw OR HHV4:ti,ab,kw OR ‘Epstein-Barr’:ti,ab,kw OR ‘E-B virus*’:ti,ab,kw OR EBV:ti,ab,kw OR burkitt*:ti,ab,kw OR mononucleo*:ti,ab,kw OR leukopla*:ti,ab,kw

#21 coronavir*:ti,ab,kw OR corona-vir*:ti,ab,kw OR alphacoronavir*:ti,ab,kw OR betacoronavir*:ti,ab,kw OR SARS:ti,ab,kw OR MERS:ti,ab,kw OR SARS2:ti,ab,kw OR ‘SARS-COV-2’:ti,ab,kw OR COVID*:ti,ab,kw OR 2019nCoV:ti,ab,kw OR ‘2019-nCoV’:ti,ab,kw OR ‘HCoV-19’:ti,ab,kw OR ‘severe acute respiratory syndrome’:ti,ab,kw OR ‘Middle East respiratory syndrome’:ti,ab,kw

#22 flavivir*:ti,ab,kw OR arbovir*:ti,ab,kw OR ‘yellow fever’:ti,ab,kw OR YFV:ti,ab,kw OR dengue:ti,ab,kw OR DENV:ti,ab,kw OR ‘breakbone fever’:ti,ab,kw OR ‘West Nile’:ti,ab,kw OR WNV:ti,ab,kw OR ‘tick borne’:ti,ab,kw OR ‘Japanese encephalitis’:ti,ab,kw OR ‘Saint Louis’:ti,ab,kw OR ‘St Louis’:ti,ab,kw OR zika:ti,ab,kw OR zikV:ti,ab,kw OR hepacivir*:ti,ab,kw OR ‘hepatitis C’:ti,ab,kw

#23 hepadnavir*:ti,ab,kw OR orthohepadnavir*:ti,ab,kw OR HBV:ti,ab,kw OR ‘hepatitis B’:ti,ab,kw

#24 picornavir*:ti,ab,kw OR hepatovir*:ti,ab,kw OR ‘hepatitis A’:ti,ab,kw OR HAV:ti,ab,kw OR enterovir*:ti,ab,kw OR echovir*:ti,ab,kw OR coxsackie*:ti,ab,kw OR CVB3:ti,ab,kw OR cardiovir*:ti,ab,kw OR herpangin*:ti,ab,kw

#25 hepevir*:ti,ab,kw OR HEV:ti,ab,kw OR ‘hepatitis E’:ti,ab,kw

#26 papillomavir*:ti,ab,kw OR alphapapillomavir*:ti,ab,kw OR betapapillomavir*:ti,ab,kw OR gammapapillomavir*:ti,ab,kw OR mupapillomavir*:ti,ab,kw OR HPV:ti,ab,kw OR wart:ti,ab,kw

#27 filovir*:ti,ab,kw OR ebola*:ti,ab,kw OR EBOV:ti,ab,kw OR marburg*:ti,ab,kw OR MARV:ti,ab,kw

#28 togavir*:ti,ab,kw OR alphavir*:ti,ab,kw OR ‘alpha vir*’:ti,ab,kw OR chikungunya:ti,ab,kw OR CHIKV:ti,ab,kw OR ‘Onyong-nyong’:ti,ab,kw OR ‘nyong nyong’:ti,ab,kw OR ‘barmah forest’:ti,ab,kw OR ‘ross river’:ti,ab,kw OR Sindbis:ti,ab,kw

#29 hantavir* OR orthohantavir*:ti,ab,kw OR hantaan*:ti,ab,kw OR ‘Andes vir*’:ti,ab,kw OR ADNV:ti,ab,kw OR puumala:ti,ab,kw OR ‘Seoul vir*’:ti,ab,kw OR SEOV:ti,ab,kw OR ‘sin nombre’:ti,ab,kw OR SNV:ti,ab,kw

#30 anellovir*:ti,ab,kw OR alphatorquevir*:ti,ab,kw OR ‘torque teno’:ti,ab,kw OR ‘TT vir*’:ti,ab,kw OR TTV:ti,ab,kw OR ‘Transfusion Transmitted vir*’:ti,ab,kw OR ‘SEN vir*’:ti,ab,kw OR SENV:ti,ab,kw OR SEN-V:ti,ab,kw

#31 matonavir*:ti,ab,kw OR rubivir*:ti,ab,kw OR rubella:ti,ab,kw OR ‘German measles’:ti,ab,kw OR RuV:ti,ab,kw

**#32 #16 OR #17 OR #18 OR #19 OR #20 OR #21 OR #22 OR #23 OR #24 OR #25 OR #26 OR #27 OR #28 OR #29 OR #30 OR #31**

#33 'virology'/de

#34 'microbiology'/de

#35 'isolation and purification'/exp

#36 'virus examination'/exp

#37 'virus shedding'/exp

#38 'virus load'/exp

#39 'virus concentration'/exp

#40 'virus transmission'/exp

#41 'virus dna'/exp/dd_an

#42 'virus rna'/exp/dd_an

#43 'in vitro study'/exp

#44 'polymerase chain reaction'/exp

#45 'polymerase chain reaction system'/exp

#46 isolat*:ti,ab,kw OR detect*:ti,ab,kw OR identif*:ti,ab,kw OR found:ti,ab,kw OR localised:ti,ab,kw OR localized:ti,ab,kw OR presence:ti,ab,kw OR level:ti,ab,kw OR levels:ti,ab,kw OR shed:ti,ab,kw OR shedding:ti,ab,kw OR concentrat*:ti,ab,kw OR load:ti,ab,kw OR loads:ti,ab,kw OR ‘in vitro’:ti,ab,kw OR ‘polymerase chain reaction’:ti,ab,kw OR PCR:ti,ab,kw OR culture*:ti,ab,kw

**#47 #33 OR #34 OR #35 OR #36 OR #37 OR #38 OR #39 OR #40 OR #41 OR #42 OR #43 OR #44 OR #45 OR #46**

#48 #15 AND #32 AND 47

#49 #48 AND [embase]/lim

# **Virus transmission through breastfeeding**

## PubMed

#1 “Milk, Human”[Mesh]

#2 "Milk Banks"[Mesh]

#3 “human milk”[tiab] OR “milk bank*”[tiab] OR milkbank*[tiab] OR “donor milk”[tiab] OR “woman milk”[tiab] OR “woman’s milk”[tiab] OR “women milk”[tiab] OR “women’s milk”[tiab]

#4 “Breast Feeding”[Mesh]

#5 “Lactation”[Mesh]

#6 “breast milk”[tiab] OR breastmilk[tiab] OR “breast-feed*”[tiab] OR breastfeed*[tiab] OR breast-fed[tiab] OR breastfed[tiab] OR lactation*[tiab] OR lactating*[tiab] OR “mothers milk”[tiab] OR “mother milk”[tiab] OR “maternal milk”[tiab] OR “nursing mother*”[tiab]

#7 #4 OR #5 OR #6

#8 “Animals”[Mesh]

#9 “Humans”[Mesh]

#10 #8 NOT #9

#11 #7 NOT #10

**#12 #1 OR #2 OR #3 OR #11**

#13 "Viruses"[Mesh]

#14 "Virus Diseases"[Mesh]

#15 viruses[title] OR “virus diseases”[title] OR “viral diseases”[title] OR “virus infections”[title] OR “viral infections”[title]

#16 retrovir*[tiab] OR leukovir*[tiab] OR oncovir*[tiab] OR deltaretrovir*[tiab] OR lentivir*[tiab] OR HIV[tiab] OR HIVs[tiab] OR “human immunodeficiency vir*”[tiab] OR “acquired immunodeficiency syndrome”[tiab] OR AIDS[tiab] OR HTLV[tiab] OR “T lymphotropic vir*”[tiab] OR “T-cell leukemia-lymphoma vir*”[tiab] OR “T-cell leukemia vir*”[tiab] OR “mammary tumor vir*”[tiab] OR HMTV[tiab] OR H-MTV[tiab]

#17 herpesvir*[tiab] OR alphaherpesvir*[tiab] OR simplexvir*[tiab] OR varicellovir*[tiab] OR betaherpesvir*[tiab] OR roseolovir*[tiab] OR rhadinovir*[tiab] OR gammaherpesvir*[tiab] OR lymphocryptovir*[tiab] OR cytomegalovir*[tiab] OR “cytomegalic vir*”[tiab] OR “salivary gland vir*”[tiab] OR CMV[tiab] OR HCMV[tiab] OR “inclusion disease*”[tiab] OR herpes[tiab] OR herpet*[tiab] OR “HHV-5”[tiab] OR HHV5[tiab] OR “HHV-1”[tiab] OR HHV1[tiab] OR “HSV-1”[tiab] OR HSV1[tiab] OR “HHV-2”[tiab] OR HHV2[tiab] OR “HSV-2”[tiab] OR HSV2[tiab] OR “HHV-3”[tiab] OR HHV3[tiab] OR “HSV-3”[tiab] OR HSV3[tiab] OR varicella*[tiab] OR zoster[tiab] OR shingles[tiab] OR “VZ vir*”[tiab] OR VZV[tiab] OR chickenpox[tiab] OR “human B-lymphotropic vir*”[tiab] OR “HHV-6*”[tiab] OR HHV6*[tiab] OR HBLV[tiab] OR roseola[tiab] OR “exanthema subitum”[tiab] OR “HHV-7”[tiab] OR HHV7[tiab] OR “HHV-8”[tiab] OR HHV8[tiab] OR KSHV[tiab] OR “Kaposi*”[tiab] OR “HHV-4”[tiab] OR HHV4[tiab] OR “Epstein-Barr”[tiab] OR “E-B virus*”[tiab] OR EBV[tiab] OR burkitt*[tiab] OR mononucleo*[tiab] OR leukopla*[tiab]

#18 "SARS-CoV-2 variants"[Supplementary Concept]

#19 coronavir*[tiab] OR corona-vir*[tiab] OR alphacoronavir*[tiab] OR betacoronavir*[tiab] OR SARS[tiab] OR MERS[tiab] OR SARS2[tiab] OR “SARS-COV-2”[tiab] OR COVID*[tiab] OR 2019nCoV[tiab] OR "2019-nCoV"[tiab] OR "HCoV-19"[tiab] OR “severe acute respiratory syndrome”[tiab] OR “Middle East respiratory syndrome”[tiab]

#20 flavivir*[tiab] OR arbovir*[tiab] OR “yellow fever”[tiab] OR YFV[tiab] OR dengue[tiab] OR DENV[tiab] OR “breakbone fever”[tiab] OR “West Nile”[tiab] OR WNV[tiab] OR “tick borne”[tiab] OR “Japanese encephalitis”[tiab] OR “Saint Louis”[tiab] OR “St Louis”[tiab] OR zika[tiab] OR zikV[tiab] OR hepacivir*[tiab] OR “hepatitis C”[tiab]

#21 hepadnavir*[tiab] OR orthohepadnavir*[tiab] OR HBV[tiab] OR “hepatitis B”[tiab]

#22 picornavir*[tiab] OR hepatovir*[tiab] OR “hepatitis A”[tiab] OR HAV[tiab] OR enterovir*[tiab] OR echovir*[tiab] OR coxsackie*[tiab] OR CVB3[tiab] OR cardiovir*[tiab] OR herpangin*[tiab]

#23 hepevir*[tiab] OR HEV[tiab] OR “hepatitis E”[tiab]

#24 papillomavir*[tiab] OR alphapapillomavir*[tiab] OR betapapillomavir*[tiab] OR gammapapillomavir*[tiab] OR mupapillomavir*[tiab] OR HPV[tiab] OR wart[tiab]

#25 filovir*[tiab] OR ebola*[tiab] OR EBOV[tiab] OR marburg*[tiab] OR MARV[tiab]

#26 togavir*[tiab] OR alphavir*[tiab] OR “alpha vir*”[tiab] OR chikungunya[tiab] OR CHIKV[tiab] OR “O'nyong-nyong”[tiab] OR “nyong nyong”[tiab] OR “barmah forest”[tiab] OR “ross river”[tiab] OR Sindbis[tiab]

#27 hantavir* OR orthohantavir*[tiab] OR hantaan*[tiab] OR “Andes vir*”[tiab] OR ADNV[tiab] OR puumala[tiab] OR “Seoul vir*”[tiab] OR SEOV[tiab] OR “sin nombre”[tiab] OR SNV[tiab]

#28 anellovir*[tiab] OR alphatorquevir*[tiab] OR “torque teno”[tiab] OR “TT vir*”[tiab] OR TTV[tiab] OR “Transfusion Transmitted vir*”[tiab] OR “SEN vir*”[tiab] OR SENV[tiab] OR SEN-V[tiab]

#29 matonavir*[tiab] OR rubivir*[tiab] OR rubella[tiab] OR “German measles”[tiab] OR RuV[tiab]

**#30 #13 OR #14 OR #15 OR #16 OR #17 OR #18 OR #19 OR #20 OR #21 OR #22 OR #23 OR #24 OR #25 OR #26 OR #27 OR #28 OR #29**

#31 "Virus Diseases/transmission"[Mesh]

#32 "Infectious Disease Transmission, Vertical"[Mesh]

#33 transmissi*[tiab] OR transmit*[tiab] OR MTCT[tiab]

#34 postnatal*[tiab] OR post-natal*[tiab] OR “mother-to-child*”[tiab] OR “mothers-to-child*”[tiab] OR “mother-to-preterm*”[tiab] OR “mothers-to-preterm*”[tiab] OR “mother-to-infant*”[tiab] OR “mothers-to-infant*”[tiab] OR “mother-to-newborn*”[tiab] OR “mothers-to-newborn*”[tiab] OR “mother-bab*”[tiab] OR “mothers-bab*”[tiab] OR vertical*[tiab]

#35 infection*[tiab] OR acquired[tiab]

#36 #34 AND #35

**#37 #31 OR #32 OR #33 OR #36**

#38 #12 AND #30 AND #37

## Embase

#1 'milk bank'/exp

#2 'human milk':ti,ab,kw OR 'milk bank*':ti,ab,kw OR milkbank*:ti,ab,kw OR ‘donor milk’:ti,ab,kw OR ‘woman milk’:ti,ab,kw OR ‘woman s milk’:ti,ab,kw OR ‘women milk’:ti,ab,kw OR ‘women s milk’:ti,ab,kw

#3 'breast milk'/exp

#4 'breast feeding'/exp

#5 'lactation'/exp

#6 'breast milk':ti,ab,kw OR breastmilk:ti,ab,kw OR 'breast-feed*':ti,ab,kw OR 'breast-fed':ti,ab,kw OR breastfed:ti,ab,kw OR lactation*:ti,ab,kw OR lactating:ti,ab,kw OR 'mothers milk':ti,ab,kw OR 'mother milk':ti,ab,kw OR 'maternal milk':ti,ab,kw OR 'nursing mother*':ti,ab,kw

#7 #3 OR #4 OR #5 OR #6

#8 'animal'/de

#9 'nonhuman'/de

#10 'animal experiment'/exp

#11 'human'/de

#12 #8 OR #9 OR #10

#13 #12 NOT #11

#14 #7 NOT #13

**#15 #1 OR #2 OR #14**

#16 'virus'/exp

#17 'virus infection'/exp

#18 viruses:ti OR ‘virus diseases’:ti OR ‘viral diseases’:ti OR ‘virus infections’:ti OR ‘viral infections’:ti

#19 retrovir*:ti,ab,kw OR leukovir*:ti,ab,kw OR oncovir*:ti,ab,kw OR deltaretrovir*:ti,ab,kw OR lentivir*:ti,ab,kw OR HIV:ti,ab,kw OR HIVs:ti,ab,kw OR ‘human immunodeficiency vir*’:ti,ab,kw OR ‘acquired immunodeficiency syndrome’:ti,ab,kw OR AIDS:ti,ab,kw OR HTLV:ti,ab,kw OR ‘T lymphotropic vir*’:ti,ab,kw OR ‘T-cell leukemia-lymphoma vir*’:ti,ab,kw OR ‘T-cell leukemia vir*’:ti,ab,kw OR ‘mammary tumor vir*’:ti,ab,kw OR HMTV:ti,ab,kw OR H-MTV:ti,ab,kw

#20 herpesvir*:ti,ab,kw OR alphaherpesvir*:ti,ab,kw OR simplexvir*:ti,ab,kw OR varicellovir*:ti,ab,kw OR betaherpesvir*:ti,ab,kw OR roseolovir*:ti,ab,kw OR rhadinovir*:ti,ab,kw OR gammaherpesvir*:ti,ab,kw OR lymphocryptovir*:ti,ab,kw OR cytomegalovir*:ti,ab,kw OR ‘cytomegalic vir*’:ti,ab,kw OR ‘salivary gland vir*’:ti,ab,kw OR CMV:ti,ab,kw OR HCMV:ti,ab,kw OR ‘inclusion disease*’:ti,ab,kw OR herpes:ti,ab,kw OR herpet*:ti,ab,kw OR ‘HHV-5’:ti,ab,kw OR HHV5:ti,ab,kw OR ‘HHV-1’:ti,ab,kw OR HHV1:ti,ab,kw OR ‘HSV-1’:ti,ab,kw OR HSV1:ti,ab,kw OR ‘HHV-2’:ti,ab,kw OR HHV2:ti,ab,kw OR ‘HSV-2’:ti,ab,kw OR HSV2:ti,ab,kw OR ‘HHV-3’:ti,ab,kw OR HHV3:ti,ab,kw OR ‘HSV-3’:ti,ab,kw OR HSV3:ti,ab,kw OR varicella*:ti,ab,kw OR zoster:ti,ab,kw OR shingles:ti,ab,kw OR ‘VZ vir*’:ti,ab,kw OR VZV:ti,ab,kw OR chickenpox:ti,ab,kw OR ‘human B-lymphotropic vir*’:ti,ab,kw OR ‘HHV-6*’:ti,ab,kw OR HHV6*:ti,ab,kw OR HBLV:ti,ab,kw OR roseola:ti,ab,kw OR ‘exanthema subitum’:ti,ab,kw OR ‘HHV-7’:ti,ab,kw OR HHV7:ti,ab,kw OR ‘HHV-8’:ti,ab,kw OR HHV8:ti,ab,kw OR KSHV:ti,ab,kw OR ‘Kaposi*’:ti,ab,kw OR ‘HHV-4’:ti,ab,kw OR HHV4:ti,ab,kw OR ‘Epstein-Barr’:ti,ab,kw OR ‘E-B virus*’:ti,ab,kw OR EBV:ti,ab,kw OR burkitt*:ti,ab,kw OR mononucleo*:ti,ab,kw OR leukopla*:ti,ab,kw

#21 coronavir*:ti,ab,kw OR corona-vir*:ti,ab,kw OR alphacoronavir*:ti,ab,kw OR betacoronavir*:ti,ab,kw OR SARS:ti,ab,kw OR MERS:ti,ab,kw OR SARS2:ti,ab,kw OR ‘SARS-COV-2’:ti,ab,kw OR COVID*:ti,ab,kw OR 2019nCoV:ti,ab,kw OR ‘2019-nCoV’:ti,ab,kw OR ‘HCoV-19’:ti,ab,kw OR ‘severe acute respiratory syndrome’:ti,ab,kw OR ‘Middle East respiratory syndrome’:ti,ab,kw

#22 flavivir*:ti,ab,kw OR arbovir*:ti,ab,kw OR ‘yellow fever’:ti,ab,kw OR YFV:ti,ab,kw OR dengue:ti,ab,kw OR DENV:ti,ab,kw OR ‘breakbone fever’:ti,ab,kw OR ‘West Nile’:ti,ab,kw OR WNV:ti,ab,kw OR ‘tick borne’:ti,ab,kw OR ‘Japanese encephalitis’:ti,ab,kw OR ‘Saint Louis’:ti,ab,kw OR ‘St Louis’:ti,ab,kw OR zika:ti,ab,kw OR zikV:ti,ab,kw OR hepacivir*:ti,ab,kw OR ‘hepatitis C’:ti,ab,kw

#23 hepadnavir*:ti,ab,kw OR orthohepadnavir*:ti,ab,kw OR HBV:ti,ab,kw OR ‘hepatitis B’:ti,ab,kw

#24 picornavir*:ti,ab,kw OR hepatovir*:ti,ab,kw OR ‘hepatitis A’:ti,ab,kw OR HAV:ti,ab,kw OR enterovir*:ti,ab,kw OR echovir*:ti,ab,kw OR coxsackie*:ti,ab,kw OR CVB3:ti,ab,kw OR cardiovir*:ti,ab,kw OR herpangin*:ti,ab,kw

#25 hepevir*:ti,ab,kw OR HEV:ti,ab,kw OR ‘hepatitis E’:ti,ab,kw

#26 papillomavir*:ti,ab,kw OR alphapapillomavir*:ti,ab,kw OR betapapillomavir*:ti,ab,kw OR gammapapillomavir*:ti,ab,kw OR mupapillomavir*:ti,ab,kw OR HPV:ti,ab,kw OR wart:ti,ab,kw

#27 filovir*:ti,ab,kw OR ebola*:ti,ab,kw OR EBOV:ti,ab,kw OR marburg*:ti,ab,kw OR MARV:ti,ab,kw

#28 togavir*:ti,ab,kw OR alphavir*:ti,ab,kw OR ‘alpha vir*’:ti,ab,kw OR chikungunya:ti,ab,kw OR CHIKV:ti,ab,kw OR ‘Onyong-nyong’:ti,ab,kw OR ‘nyong nyong’:ti,ab,kw OR ‘barmah forest’:ti,ab,kw OR ‘ross river’:ti,ab,kw OR Sindbis:ti,ab,kw

#29 hantavir* OR orthohantavir*:ti,ab,kw OR hantaan*:ti,ab,kw OR ‘Andes vir*’:ti,ab,kw OR ADNV:ti,ab,kw OR puumala:ti,ab,kw OR ‘Seoul vir*’:ti,ab,kw OR SEOV:ti,ab,kw OR ‘sin nombre’:ti,ab,kw OR SNV:ti,ab,kw

#30 anellovir*:ti,ab,kw OR alphatorquevir*:ti,ab,kw OR ‘torque teno’:ti,ab,kw OR ‘TT vir*’:ti,ab,kw OR TTV:ti,ab,kw OR ‘Transfusion Transmitted vir*’:ti,ab,kw OR ‘SEN vir*’:ti,ab,kw OR SENV:ti,ab,kw OR SEN-V:ti,ab,kw

#31 matonavir*:ti,ab,kw OR rubivir*:ti,ab,kw OR rubella:ti,ab,kw OR ‘German measles’:ti,ab,kw OR RuV:ti,ab,kw

**#32 #16 OR #17 OR #18 OR #19 OR #20 OR #21 OR #22 OR #23 OR #24 OR #25 OR #26 OR #27 OR #28 OR #29 OR #30 OR #31**

#33 'disease transmission'/exp

#34 transmissi*:ti,ab,kw OR transmit*:ti,ab,kw OR MTCT:ti,ab,kw

#35 postnatal*:ti,ab,kw OR post-natal*:ti,ab,kw OR ‘mother-to-child*’:ti,ab,kw OR ‘mothers-to-child*’:ti,ab,kw OR ‘mother-to-preterm*’:ti,ab,kw OR ‘mothers-to-preterm*’:ti,ab,kw OR ‘mother-to-infant*’:ti,ab,kw OR ‘mothers-to-infant*’:ti,ab,kw OR ‘mother-to-newborn*’:ti,ab,kw OR ‘mothers-to-newborn*’:ti,ab,kw OR ‘mother-bab*’:ti,ab,kw OR ‘mothers-bab*’:ti,ab,kw OR vertical*:ti,ab,kw

#36 infection*:ti,ab,kw OR acquired:ti,ab,kw

#37 #35 AND #36

**#38 #33 OR #34 OR #37**

#39 #15 AND #32 AND #38

#40 #39 AND [embase]/lim

# **The antiviral activity of Human Milk**

## PubMed

#1 “Milk, Human”[Mesh]

#2 "Milk Banks"[Mesh]

#3 “human milk”[tiab] OR “milk bank*”[tiab] OR milkbank*[tiab] OR “donor milk”[tiab] OR “woman milk”[tiab] OR “woman’s milk”[tiab] OR “women milk”[tiab] OR “women’s milk”[tiab] OR “breast milk”[tiab] OR breastmilk[tiab] OR “mothers milk”[tiab] OR “mother milk”[tiab] OR “maternal milk”[tiab]

#4 “Breast Feeding”[Mesh]

#5 “breast-feed*”[tiab] OR breastfeed*[tiab] OR breast-fed[tiab] OR breastfed[tiab]

#6 “Lactation”[Mesh]

#7 lactation*[tiab] OR lactating*[tiab] OR “nursing mother*”[tiab]

#8 #1 OR #2 OR #3 OR #4 OR #5 OR #6 OR #7

#9 “antiviral propert*”[tiab] OR “anti-viral propert*”[tiab] OR “anti-infective propert*”[tiab] OR “antiviral function*”[tiab] OR “anti-viral function*”[tiab] OR “anti-infective function*”[tiab] OR "antiviral character*"[tiab] OR "anti-viral character*"[tiab] OR “anti-infective character*”[tiab] OR “antiviral role*”[tiab] OR “anti-viral role*”[tiab] OR “anti-infective role*”[tiab] OR “antiviral qualit*”[tiab] OR “anti-viral qualit*”[tiab] OR “anti-infective qualit*”[tiab] OR “antiviral action*”[tiab] OR “anti-viral action*”[tiab] OR “anti-infective action*”[tiab] OR “antiviral activit*”[tiab] OR “anti-viral activit*”[tiab] OR “anti-infective activit*”[tiab]

**#10 #8 AND #9**

#11 "Viruses"[Mesh]

#12 "virology"[Subheading]

#13 "Virus Diseases"[Mesh]

#14 "Virus Physiological Phenomena"[Mesh]

#15 "Virus Shedding"[Mesh]

#16 virus*[tiab] OR viral*[tiab] OR virol*[tiab]

#17 antiviral*[tiab] OR anti-infective*[tiab]

#18 retrovir*[tiab] OR leukovir*[tiab] OR oncovir*[tiab] OR deltaretrovir*[tiab] OR lentivir*[tiab] OR HIV[tiab] OR HIVs[tiab] OR “acquired immunodeficiency syndrome”[tiab] OR AIDS[tiab] OR HTLV[tiab] OR HMTV[tiab] OR H-MTV[tiab] OR herpesvir*[tiab] OR alphaherpesvir*[tiab] OR simplexvir*[tiab] OR varicellovir*[tiab] OR betaherpesvir*[tiab] OR roseolovir*[tiab] OR rhadinovir*[tiab] OR gammaherpesvir*[tiab] OR lymphocryptovir*[tiab] OR cytomegalovir*[tiab] OR CMV[tiab] OR HCMV[tiab] OR “inclusion disease*”[tiab] OR herpes[tiab] OR herpet*[tiab] OR “HHV-5”[tiab] OR HHV5[tiab] OR “HHV-1”[tiab] OR HHV1[tiab] OR “HSV-1”[tiab] OR HSV1[tiab] OR “HHV-2”[tiab] OR HHV2[tiab] OR “HSV-2”[tiab] OR HSV2[tiab] OR “HHV-3”[tiab] OR HHV3[tiab] OR “HSV-3”[tiab] OR HSV3[tiab] OR varicella*[tiab] OR zoster[tiab] OR shingles[tiab] OR VZV[tiab] OR chickenpox[tiab] OR “HHV-6*”[tiab] OR HHV6*[tiab] OR HBLV[tiab] OR roseola[tiab] OR “exanthema subitum”[tiab] OR “HHV-7”[tiab] OR HHV7[tiab] OR “HHV-8”[tiab] OR HHV8[tiab] OR KSHV[tiab] OR “Kaposi*”[tiab] OR “HHV-4”[tiab] OR HHV4[tiab] OR “Epstein-Barr”[tiab] OR EBV[tiab] OR burkitt*[tiab] OR mononucleo*[tiab] OR leukopla*[tiab] OR coronavir*[tiab] OR alphacoronavir*[tiab] OR betacoronavir*[tiab] OR SARS[tiab] OR MERS[tiab] OR SARS2[tiab] OR “SARS-COV-2”[tiab] OR COVID*[tiab] OR 2019nCoV[tiab] OR "2019-nCoV"[tiab] OR "HCoV-19"[tiab] OR “severe acute respiratory syndrome”[tiab] OR “Middle East respiratory syndrome”[tiab]

#19 flavivir*[tiab] OR arbovir*[tiab] OR “yellow fever”[tiab] OR YFV[tiab] OR dengue[tiab] OR DENV[tiab] OR “breakbone fever”[tiab] OR “West Nile”[tiab] OR WNV[tiab] OR “tick borne”[tiab] OR “Japanese encephalitis”[tiab] OR “Saint Louis”[tiab] OR “St Louis”[tiab] OR zika[tiab] OR zikV[tiab] OR hepacivir*[tiab] OR “hepatitis C”[tiab] OR hepadnavir*[tiab] OR orthohepadnavir*[tiab] OR HBV[tiab] OR “hepatitis B”[tiab] OR picornavir*[tiab] OR hepatovir*[tiab] OR “hepatitis A”[tiab] OR HAV[tiab] OR enterovir*[tiab] OR echovir*[tiab] OR coxsackie*[tiab] OR CVB3[tiab] OR cardiovir*[tiab] OR herpangin*[tiab] OR hepevir*[tiab] OR HEV[tiab] OR “hepatitis E”[tiab] OR papillomavir*[tiab] OR alphapapillomavir*[tiab] OR betapapillomavir*[tiab] OR gammapapillomavir*[tiab] OR mupapillomavir*[tiab] OR HPV[tiab] OR wart[tiab] OR filovir*[tiab] OR ebola*[tiab] OR EBOV[tiab] OR marburg*[tiab] OR MARV[tiab] OR togavir*[tiab] OR alphavir*[tiab] OR chikungunya[tiab] OR CHIKV[tiab] OR “O'nyong-nyong”[tiab] OR “nyong nyong”[tiab] OR “barmah forest”[tiab] OR “ross river”[tiab] OR Sindbis[tiab] OR hantavir* OR orthohantavir*[tiab] OR hantaan*[tiab] OR ADNV[tiab] OR puumala[tiab] OR SEOV[tiab] OR “sin nombre”[tiab] OR SNV[tiab] OR anellovir*[tiab] OR alphatorquevir*[tiab] OR “torque teno”[tiab] OR TTV[tiab] OR SENV[tiab] OR SEN-V[tiab] OR matonavir*[tiab] OR rubivir*[tiab] OR rubella[tiab] OR “German measles”[tiab] OR RuV[tiab]

#20 #11 OR #12 OR #13 OR #14 OR #15 OR #16 OR #17 OR #18 OR #19

#21 "Milk, Human/immunology"[Mesh]

#22 "Immunoglobulins"[Mesh]

#23 immunoglobulin*[tiab] OR globulin*[tiab] OR antibod*[tiab] OR IgA[tiab] OR IgG[tiab] OR IgD[tiab] OR IgE[tiab] OR IgM[tiab]

#24 "Mucins"[Mesh]

#25 mucin*[tiab] OR MUC1[tiab] OR MUC2[tiab] OR MUC3[tiab] OR MUC4[tiab] OR MUC5*[tiab] OR MUC6*[tiab]

#26 "Oxysterols"[Mesh]

#27 oxysterol*[tiab] OR hydroxycholesterol*[tiab] OR ketocholesterol*[tiab] OR 25OHC[tiab] OR 27OHC[tiab] OR 24SOHC[tiab]

#28 "Tenascin"[Mesh]

#29 "TNC protein, human"[Supplementary Concept]

#30 tenascin*[tiab] OR TNC[tiab]

#31 "Lipids"[Mesh:NoExp]

#32 lipid*[tiab] OR fats[tiab] OR fat[tiab] OR fatty[tiab]

#33 "Glycolipids"[Mesh]

#34 glycolipid*[tiab] OR glycosphingolipid*[tiab] OR ceramid*[tiab] OR cerebroside*[tiab] OR galactocerebroside*[tiab] OR glucocerebroside*[tiab] OR globoside*[tiab] OR lactosylceramid*[tiab] OR galactosylceramid*[tiab] OR trihexosylceramid*[tiab] OR glucosylceramid*[tiab] OR globotriaosylceramid*[tiab]

#35 "Polysaccharides"[Mesh:noexp]

#36 glycan*[tiab] OR polysaccharid*[tiab]

#37 "Oligosaccharides"[Mesh]

#38 oligosaccharid*[tiab] OR disaccharid*[tiab] OR monosaccharid*[tiab] OR HMO[tiab] OR HMOs[tiab]

#39 "Glycosaminoglycans"[Mesh]

#40 glycosaminoglycan*[tiab] OR mucopolysaccharid*[tiab] OR GAGs[tiab] OR chondroitin[tiab] OR dermatan[tiab] OR heparin[tiab] OR “hyaluronic acid”[tiab]

#41 "Cytokines"[Mesh]

#42 cytokin*[tiab] OR chemokin*[tiab] OR interleukin*[tiab] OR “tumor necrosis factor*”[tiab] OR “growth factor*”[tiab]

#43 "Extracellular Vesicles"[Mesh]

#44 “extracellular vesicle*”[tiab] OR “extra-cellular vesicle*”[tiab] OR “exovesicle*”[tiab] OR “apoptotic bod*”[tiab] OR exosom*[tiab] OR ectosom*[tiab] OR “cell-derived microparticle*”[tiab] OR “cell membrane microparticle*”[tiab] OR “shedding microvesicle*”[tiab]

#45 "Secretory Leukocyte Peptidase Inhibitor"[Mesh]

#46 "SLPI protein, human"[Supplementary Concept]

#47 “peptidase inhibitor”[tiab] OR “protease inhibitor”[tiab] OR SLPI[tiab] OR “leukoprotease inhibitor”[tiab] OR antileukoprotease[tiab]

#48 "Toll-Like Receptors"[Mesh]

#49 “toll-like receptor*”[tiab] OR sTLR*[tiab] OR TLR1*[tiab] OR TLR2[tiab] OR TLR3[tiab] OR TLR4[tiab] OR TLR5[tiab] OR TLR6[tiab] OR TLR7[tiab] OR TLR8[tiab] OR TLR9[tiab]

#50 "Lewis X Antigen"[Mesh]

#51 “lewis X”[tiab] OR “lewis antigen*”[tiab] OR CD15*[tiab] OR “sialyl-lex”[tiab]

#52 "Tryptophan"[Mesh]

#53 tryptophan*[tiab]

#54 "Prostaglandins"[Mesh]

#55 prostaglandin*[tiab]

#56 "Lactoferrin"[Mesh]

#57 "LTF protein, human"[Supplementary Concept]

#58 "lactoferrin (1-11), human"[Supplementary Concept]

#59 "ITLN1 protein, human"[Supplementary Concept]

#60 lactoferrin*[tiab] OR lactotransferrin*[tiab]

#61 "MFGE8 protein, human"[Supplementary Concept]

#62 "milk fat globule"[Supplementary Concept]

#63 lactadherin*[tiab] OR “milk fat globule*”[tiab] OR “MFG-E8”[tiab] OR MFGE8[tiab] OR MFGM[tiab]

#64 #21 OR #22 OR #23 OR #24 OR #25 OR #26 OR #27 #OR #28 OR #29 OR #30 OR #31 OR #32 OR #33 OR #34 OR #35 OR #36 OR #37 #OR #38 OR #39 OR #40 OR #41 OR #42 OR #43 OR #44 OR #45 OR #46 OR #47 #OR #48 OR #49 OR #50 OR #51 OR #52 OR #53 OR #54 OR #55 OR #56 OR #57 #OR #58 OR #59 OR #60 OR #61 OR #62 OR #63

**#65 #8 AND #20 AND #64**

#66 #10 OR #65

## Embase

#1 'milk bank'/exp

#2 'breast milk'/exp

#3 'human milk':ti,ab,kw OR 'milk bank*':ti,ab,kw OR milkbank*:ti,ab,kw OR ‘donor milk’:ti,ab,kw OR ‘woman milk’:ti,ab,kw OR ‘woman s milk’:ti,ab,kw OR ‘women milk’:ti,ab,kw OR ‘women s milk’:ti,ab,kw OR 'breast milk':ti,ab,kw OR breastmilk:ti,ab,kw OR 'mothers milk':ti,ab,kw OR 'mother milk':ti,ab,kw OR 'maternal milk':ti,ab,kw

#4 'breast feeding'/exp

#5 'breast-feed*':ti,ab,kw OR 'breast-fed':ti,ab,kw OR breastfed:ti,ab,kw

#6 'lactation'/exp

#7 lactation*:ti,ab,kw OR lactating:ti,ab,kw OR 'nursing mother*':ti,ab,kw

#8 #1 OR #2 OR #3 OR #4 OR #5 OR #6 OR #7

#9 ‘antiviral propert*’:ti,ab,kw OR ‘anti-viral propert*’:ti,ab,kw OR ‘anti-infective propert*’:ti,ab,kw OR ‘antiviral function*’:ti,ab,kw OR ‘anti-viral function*’:ti,ab,kw OR ‘anti-infective function*’:ti,ab,kw OR ‘antiviral character*’:ti,ab,kw OR ‘anti-viral character*’:ti,ab,kw OR ‘anti-infective character*’:ti,ab,kw OR ‘antiviral role*’:ti,ab,kw OR ‘anti-viral role*’:ti,ab,kw OR ‘anti-infective role*’:ti,ab,kw OR ‘antiviral qualit*’:ti,ab,kw OR ‘anti-viral qualit*’:ti,ab,kw OR ‘anti-infective qualit*’:ti,ab,kw OR ‘antiviral action*’:ti,ab,kw OR ‘anti-viral action*’:ti,ab,kw OR ‘anti-infective action*’:ti,ab,kw OR ‘antiviral activit*’:ti,ab,kw OR ‘anti-viral activit*’:ti,ab,kw OR ‘anti-infective activit*’:ti,ab,kw

**#10 #8 AND #9**

#11 'virus'/exp

#12 'virus infection'/exp

#13 'virus examination'/exp

#14 'viral phenomena and functions'/exp

#15 'virus load'/exp

#16 virus*:ti,ab,kw OR viral*:ti,ab,kw OR virol*:ti,ab,kw

#17 antiviral*:ti,ab,kw OR anti-infective*:ti,ab,kw

#18 retrovir*:ti,ab,kw OR leukovir*:ti,ab,kw OR oncovir*:ti,ab,kw OR deltaretrovir*:ti,ab,kw OR lentivir*:ti,ab,kw OR HIV:ti,ab,kw OR HIVs:ti,ab,kw OR ‘acquired immunodeficiency syndrome’:ti,ab,kw OR AIDS:ti,ab,kw OR HTLV:ti,ab,kw OR HMTV:ti,ab,kw OR H-MTV:ti,ab,kw OR herpesvir*:ti,ab,kw OR alphaherpesvir*:ti,ab,kw OR simplexvir*:ti,ab,kw OR varicellovir*:ti,ab,kw OR betaherpesvir*:ti,ab,kw OR roseolovir*:ti,ab,kw OR rhadinovir*:ti,ab,kw OR gammaherpesvir*:ti,ab,kw OR lymphocryptovir*:ti,ab,kw OR cytomegalovir*:ti,ab,kw OR CMV:ti,ab,kw OR HCMV:ti,ab,kw OR ‘inclusion disease*’:ti,ab,kw OR herpes:ti,ab,kw OR herpet*:ti,ab,kw OR ‘HHV-5’:ti,ab,kw OR HHV5:ti,ab,kw OR ‘HHV-1’:ti,ab,kw OR HHV1:ti,ab,kw OR ‘HSV-1’:ti,ab,kw OR HSV1:ti,ab,kw OR ‘HHV-2’:ti,ab,kw OR HHV2:ti,ab,kw OR ‘HSV-2’:ti,ab,kw OR HSV2:ti,ab,kw OR ‘HHV-3’:ti,ab,kw OR HHV3:ti,ab,kw OR ‘HSV-3’:ti,ab,kw OR HSV3:ti,ab,kw OR varicella*:ti,ab,kw OR zoster:ti,ab,kw OR shingles:ti,ab,kw OR VZV:ti,ab,kw OR chickenpox:ti,ab,kw OR ‘HHV-6*’:ti,ab,kw OR HHV6*:ti,ab,kw OR HBLV:ti,ab,kw OR roseola:ti,ab,kw OR ‘exanthema subitum’:ti,ab,kw OR ‘HHV-7’:ti,ab,kw OR HHV7:ti,ab,kw OR ‘HHV-8’:ti,ab,kw OR HHV8:ti,ab,kw OR KSHV:ti,ab,kw OR ‘Kaposi*’:ti,ab,kw OR ‘HHV-4’:ti,ab,kw OR HHV4:ti,ab,kw OR ‘Epstein-Barr’:ti,ab,kw OR EBV:ti,ab,kw OR burkitt*:ti,ab,kw OR mononucleo*:ti,ab,kw OR leukopla*:ti,ab,kw OR coronavir*:ti,ab,kw OR alphacoronavir*:ti,ab,kw OR betacoronavir*:ti,ab,kw OR SARS:ti,ab,kw OR MERS:ti,ab,kw OR SARS2:ti,ab,kw OR ‘SARS-COV-2’:ti,ab,kw OR COVID*:ti,ab,kw OR 2019nCoV:ti,ab,kw OR ‘2019-nCoV’:ti,ab,kw OR ‘HCoV-19’:ti,ab,kw OR ‘severe acute respiratory syndrome’:ti,ab,kw OR ‘Middle East respiratory syndrome’:ti,ab,kw

#19 flavivir*:ti,ab,kw OR arbovir*:ti,ab,kw OR ‘yellow fever’:ti,ab,kw OR YFV:ti,ab,kw OR dengue:ti,ab,kw OR DENV:ti,ab,kw OR ‘breakbone fever’:ti,ab,kw OR ‘West Nile’:ti,ab,kw OR WNV:ti,ab,kw OR ‘tick borne’:ti,ab,kw OR ‘Japanese encephalitis’:ti,ab,kw OR ‘Saint Louis’:ti,ab,kw OR ‘St Louis’:ti,ab,kw OR zika:ti,ab,kw OR zikV:ti,ab,kw OR hepacivir*:ti,ab,kw OR ‘hepatitis C’:ti,ab,kw OR hepadnavir*:ti,ab,kw OR orthohepadnavir*:ti,ab,kw OR HBV:ti,ab,kw OR ‘hepatitis B’:ti,ab,kw OR picornavir*:ti,ab,kw OR hepatovir*:ti,ab,kw OR ‘hepatitis A’:ti,ab,kw OR HAV:ti,ab,kw OR enterovir*:ti,ab,kw OR echovir*:ti,ab,kw OR coxsackie*:ti,ab,kw OR CVB3:ti,ab,kw OR cardiovir*:ti,ab,kw OR herpangin*:ti,ab,kw OR hepevir*:ti,ab,kw OR HEV:ti,ab,kw OR ‘hepatitis E’:ti,ab,kw OR papillomavir*:ti,ab,kw OR alphapapillomavir*:ti,ab,kw OR betapapillomavir*:ti,ab,kw OR gammapapillomavir*:ti,ab,kw OR mupapillomavir*:ti,ab,kw OR HPV:ti,ab,kw OR wart:ti,ab,kw OR filovir*:ti,ab,kw OR ebola*:ti,ab,kw OR EBOV:ti,ab,kw OR marburg*:ti,ab,kw OR MARV:ti,ab,kw OR togavir*:ti,ab,kw OR alphavir*:ti,ab,kw OR chikungunya:ti,ab,kw OR CHIKV:ti,ab,kw OR ‘Onyong-nyong’:ti,ab,kw OR ‘nyong nyong’:ti,ab,kw OR ‘barmah forest’:ti,ab,kw OR ‘ross river’:ti,ab,kw OR Sindbis:ti,ab,kw OR hantavir* OR orthohantavir*:ti,ab,kw OR hantaan*:ti,ab,kw OR ADNV:ti,ab,kw OR puumala:ti,ab,kw OR SEOV:ti,ab,kw OR ‘sin nombre’:ti,ab,kw OR SNV:ti,ab,kw OR anellovir*:ti,ab,kw OR alphatorquevir*:ti,ab,kw OR ‘torque teno’:ti,ab,kw OR TTV:ti,ab,kw OR SENV:ti,ab,kw OR SEN-V:ti,ab,kw OR matonavir*:ti,ab,kw OR rubivir*:ti,ab,kw OR rubella:ti,ab,kw OR ‘German measles’:ti,ab,kw OR RuV:ti,ab,kw

#20 #11 OR #12 OR #13 OR #14 OR #15 OR #16 OR #17 OR #18 OR #19

#21 'immunoglobulin'/exp

#22 'antibody'/exp

#23 immunoglobulin*:ti,ab,kw OR globulin*:ti,ab,kw OR antibod*:ti,ab,kw OR IgA:ti,ab,kw OR IgG:ti,ab,kw OR IgD:ti,ab,kw OR IgE:ti,ab,kw OR IgM:ti,ab,kw

#24 'mucin'/exp

#25 mucin*:ti,ab,kw OR MUC1:ti,ab,kw OR MUC2:ti,ab,kw OR MUC3:ti,ab,kw OR MUC4:ti,ab,kw OR MUC5*:ti,ab,kw OR MUC6*:ti,ab,kw

#26 'oxysterol'/exp

#27 oxysterol*:ti,ab,kw OR hydroxycholesterol*:ti,ab,kw OR ketocholesterol*:ti,ab,kw OR 25OHC:ti,ab,kw OR 27OHC:ti,ab,kw OR 24SOHC:ti,ab,kw

#28 'tenascin'/exp

#29 tenascin*:ti,ab,kw OR TNC:ti,ab,kw

#30 'lipid'/de

#31 lipid*:ti,ab,kw OR fats:ti,ab,kw OR fat:ti,ab,kw OR fatty:ti,ab,kw

#32 'glycolipid'/exp

#33 glycolipid*:ti,ab,kw OR glycosphingolipid*:ti,ab,kw OR ceramid*:ti,ab,kw OR cerebroside*:ti,ab,kw OR galactocerebroside*:ti,ab,kw OR glucocerebroside*:ti,ab,kw OR globoside*:ti,ab,kw OR lactosylceramid*:ti,ab,kw OR galactosylceramid*:ti,ab,kw OR trihexosylceramid*:ti,ab,kw OR glucosylceramid*:ti,ab,kw OR globotriaosylceramid*:ti,ab,kw

#34 'glycan'/de

#35 glycan*:ti,ab,kw OR polysaccharid*:ti,ab,kw

#36 'oligosaccharide'/exp

#37 oligosaccharid*:ti,ab,kw OR disaccharid*:ti,ab,kw OR monosaccharid*:ti,ab,kw OR HMO:ti,ab,kw OR HMOs:ti,ab,kw

#38 'glycosaminoglycan'/exp

#39 glycosaminoglycan*:ti,ab,kw OR mucopolysaccharid*:ti,ab,kw OR GAGs:ti,ab,kw OR chondroitin:ti,ab,kw OR dermatan:ti,ab,kw OR heparin:ti,ab,kw OR ‘hyaluronic acid’:ti,ab,kw

#40 'cytokine'/exp

#41 cytokin*:ti,ab,kw OR chemokin*:ti,ab,kw OR interleukin*:ti,ab,kw OR ‘tumor necrosis factor*’:ti,ab,kw OR ‘growth factor*’:ti,ab,kw

#42 'exosome'/exp

#43 ‘extracellular vesicle*’:ti,ab,kw OR ‘extra-cellular vesicle*’:ti,ab,kw OR ‘exovesicle*’:ti,ab,kw OR ‘apoptotic bod*’:ti,ab,kw OR exosom*:ti,ab,kw OR ectosom*:ti,ab,kw OR ‘cell-derived microparticle*’:ti,ab,kw OR ‘cell membrane microparticle*’:ti,ab,kw OR ‘shedding microvesicle*’:ti,ab,kw

#44 'secretory leukocyte proteinase inhibitor'/exp

#45 ‘peptidase inhibitor’:ti,ab,kw OR ‘protease inhibitor’:ti,ab,kw OR SLPI:ti,ab,kw OR ‘leukoprotease inhibitor’:ti,ab,kw OR antileukoprotease:ti,ab,kw

#46 'toll like receptor'/exp

#47 ‘toll-like receptor*’:ti,ab,kw OR sTLR*:ti,ab,kw OR TLR1*:ti,ab,kw OR TLR2:ti,ab,kw OR TLR3:ti,ab,kw OR TLR4:ti,ab,kw OR TLR5:ti,ab,kw OR TLR6:ti,ab,kw OR TLR7:ti,ab,kw OR TLR8:ti,ab,kw OR TLR9:ti,ab,kw

#48 'CD15 antigen'/exp

#49 ‘lewis X’:ti,ab,kw OR ‘lewis antigen*’:ti,ab,kw OR CD15*:ti,ab,kw OR ‘sialyl-lex’:ti,ab,kw

#50 'tryptophan'/exp

#51 tryptophan*:ti,ab,kw

#52 'prostaglandin'/exp

#53 prostaglandin*:ti,ab,kw

#54 'lactoferrin'/exp

#55 lactoferrin*:ti,ab,kw OR lactotransferrin*:ti,ab,kw

#56 'lactadherin'/exp

#57 lactadherin*:ti,ab,kw OR ‘milk fat globule*’:ti,ab,kw OR ‘MFG-E8’:ti,ab,kw OR MFGE8:ti,ab,kw OR MFGM:ti,ab,kw

#58 #21 OR #22 OR #23 OR #24 OR #25 OR #26 OR #27 #OR #28 OR #29 OR #30 OR #31 OR #32 OR #33 OR #34 OR #35 OR #36 OR #37 #OR #38 OR #39 OR #40 OR #41 OR #42 OR #43 OR #44 OR #45 OR #46 OR #47 #OR #48 OR #49 OR #50 OR #51 OR #52 OR #53 OR #54 OR #55 OR #56 OR #57

**#59 #8 AND #20 AND #58**

#60 #10 OR #59

#61 #60 AND [embase]/lim

# **The pasteurization and storage methods of Human Milk**

## PubMed

#1 “Milk, Human”[Mesh]

#2 "Milk Banks"[Mesh]

#3 “human milk”[tiab] OR “milk bank*”[tiab] OR milkbank*[tiab] OR “donor milk”[tiab] OR “woman milk”[tiab] OR “woman’s milk”[tiab] OR “women milk”[tiab] OR “women’s milk”[tiab] OR “breast milk”[tiab] OR breastmilk[tiab] OR “mothers milk”[tiab] OR “mother milk”[tiab] OR “maternal milk”[tiab]

**#4 #1 OR #2 OR #3**

#5 "Viruses"[Mesh]

#6 "virology"[Subheading]

#7 "Virus Diseases"[Mesh]

#8 "Virus Physiological Phenomena"[Mesh]

#9 "Virus Shedding"[Mesh]

#10 virus*[tiab] OR viral*[tiab] OR virol*[tiab]

#11 antiviral*[tiab] OR anti-infective*[tiab]

#12 retrovir*[tiab] OR leukovir*[tiab] OR oncovir*[tiab] OR deltaretrovir*[tiab] OR lentivir*[tiab] OR HIV[tiab] OR HIVs[tiab] OR “acquired immunodeficiency syndrome”[tiab] OR AIDS[tiab] OR HTLV[tiab] OR HMTV[tiab] OR H-MTV[tiab] OR herpesvir*[tiab] OR alphaherpesvir*[tiab] OR simplexvir*[tiab] OR varicellovir*[tiab] OR betaherpesvir*[tiab] OR roseolovir*[tiab] OR rhadinovir*[tiab] OR gammaherpesvir*[tiab] OR lymphocryptovir*[tiab] OR cytomegalovir*[tiab] OR CMV[tiab] OR HCMV[tiab] OR “inclusion disease*”[tiab] OR herpes[tiab] OR herpet*[tiab] OR “HHV-5”[tiab] OR HHV5[tiab] OR “HHV-1”[tiab] OR HHV1[tiab] OR “HSV-1”[tiab] OR HSV1[tiab] OR “HHV-2”[tiab] OR HHV2[tiab] OR “HSV-2”[tiab] OR HSV2[tiab] OR “HHV-3”[tiab] OR HHV3[tiab] OR “HSV-3”[tiab] OR HSV3[tiab] OR varicella*[tiab] OR zoster[tiab] OR shingles[tiab] OR VZV[tiab] OR chickenpox[tiab] OR “HHV-6*”[tiab] OR HHV6*[tiab] OR HBLV[tiab] OR roseola[tiab] OR “exanthema subitum”[tiab] OR “HHV-7”[tiab] OR HHV7[tiab] OR “HHV-8”[tiab] OR HHV8[tiab] OR KSHV[tiab] OR “Kaposi*”[tiab] OR “HHV-4”[tiab] OR HHV4[tiab] OR “Epstein-Barr”[tiab] OR EBV[tiab] OR burkitt*[tiab] OR mononucleo*[tiab] OR leukopla*[tiab] OR coronavir*[tiab] OR alphacoronavir*[tiab] OR betacoronavir*[tiab] OR SARS[tiab] OR MERS[tiab] OR SARS2[tiab] OR “SARS-COV-2”[tiab] OR COVID*[tiab] OR 2019nCoV[tiab] OR "2019-nCoV"[tiab] OR "HCoV-19"[tiab] OR “severe acute respiratory syndrome”[tiab] OR “Middle East respiratory syndrome”[tiab]

#13 flavivir*[tiab] OR arbovir*[tiab] OR “yellow fever”[tiab] OR YFV[tiab] OR dengue[tiab] OR DENV[tiab] OR “breakbone fever”[tiab] OR “West Nile”[tiab] OR WNV[tiab] OR “tick borne”[tiab] OR “Japanese encephalitis”[tiab] OR “Saint Louis”[tiab] OR “St Louis”[tiab] OR zika[tiab] OR zikV[tiab] OR hepacivir*[tiab] OR “hepatitis C”[tiab] OR hepadnavir*[tiab] OR orthohepadnavir*[tiab] OR HBV[tiab] OR “hepatitis B”[tiab] OR picornavir*[tiab] OR hepatovir*[tiab] OR “hepatitis A”[tiab] OR HAV[tiab] OR enterovir*[tiab] OR echovir*[tiab] OR coxsackie*[tiab] OR CVB3[tiab] OR cardiovir*[tiab] OR herpangin*[tiab] OR hepevir*[tiab] OR HEV[tiab] OR “hepatitis E”[tiab] OR papillomavir*[tiab] OR alphapapillomavir*[tiab] OR betapapillomavir*[tiab] OR gammapapillomavir*[tiab] OR mupapillomavir*[tiab] OR HPV[tiab] OR wart[tiab] OR filovir*[tiab] OR ebola*[tiab] OR EBOV[tiab] OR marburg*[tiab] OR MARV[tiab] OR togavir*[tiab] OR alphavir*[tiab] OR chikungunya[tiab] OR CHIKV[tiab] OR “O'nyong-nyong”[tiab] OR “nyong nyong”[tiab] OR “barmah forest”[tiab] OR “ross river”[tiab] OR Sindbis[tiab] OR hantavir* OR orthohantavir*[tiab] OR hantaan*[tiab] OR ADNV[tiab] OR puumala[tiab] OR SEOV[tiab] OR “sin nombre”[tiab] OR SNV[tiab] OR anellovir*[tiab] OR alphatorquevir*[tiab] OR “torque teno”[tiab] OR TTV[tiab] OR SENV[tiab] OR SEN-V[tiab] OR matonavir*[tiab] OR rubivir*[tiab] OR rubella[tiab] OR “German measles”[tiab] OR RuV[tiab]

**#14 #5 OR #6 OR #7 OR #8 OR #9 OR #10 OR #11 OR #12 OR #13**

#15 “Pasteurization”[Mesh]

#16 pasteuri*[tiab] OR holder[tiab] OR HoP[tiab] OR “low temperature long time”[tiab] OR LTLT[tiab] OR flash[tiab] OR “high temperature short time”[tiab] OR HTST[tiab] OR thermal[tiab]

#17 “Hot Temperature”[Mesh]

#18 heat[tiab] OR heating[tiab] OR nonthermal[tiab]

#19 “Hydrostatic Pressure”[Mesh]

#20 “high hydrostatic pressure”[tiab] OR HHP[tiab] OR “high-pressure processing”[tiab] OR HPP[tiab]

#21 “Ultraviolet Rays”[Mesh]

#22 ultraviolet[tiab] OR UVC[tiab] OR UV-C[tiab]

#23 "Gamma Rays"[Mesh]

#24 “gamma-irradiat*”[tiab] OR “gamma-radiat*”[tiab]

#25 "Sonication"[Mesh]

#26 sonicat*[tiab] OR sonificat*[tiab] OR ultrasound[tiab] OR ultrasonic[tiab] OR thermoultrasound[tiab] OR processing[tiab] OR irradiation*[tiab] OR inactivation*[tiab]

#27 "Freeze Drying"[Mesh]

#28 “Freezing”[Mesh]

#29 “Cold Temperature”[Mesh]

#30 “Refrigeration”[Mesh]

#31 freez*[tiab] OR frozen[tiab] OR refrigerat*[tiab] OR thaw*[tiab]

#32 “Food Preservation”[Mesh:no exp]

#33 “Food Storage”[Mesh]

#34 storag*[tiab]

**#35 #15 OR #16 OR #17 OR #18 OR #19 OR #20 OR #21 OR #22 OR #23 OR #24 OR #25 OR #26 OR à27 OR #28 OR #29 OR #30 OR #31 OR #32 OR #33 OR #34**

#36 #4 AND #14 AND #35

## Embase

#1 'milk bank'/exp

#2 'breast milk'/exp

#3 'human milk':ti,ab,kw OR 'milk bank*':ti,ab,kw OR milkbank*:ti,ab,kw OR ‘donor milk’:ti,ab,kw OR ‘woman milk’:ti,ab,kw OR ‘woman s milk’:ti,ab,kw OR ‘women milk’:ti,ab,kw OR ‘women s milk’:ti,ab,kw OR 'breast milk':ti,ab,kw OR breastmilk:ti,ab,kw OR 'mothers milk':ti,ab,kw OR 'mother milk':ti,ab,kw OR 'maternal milk':ti,ab,kw

**#4 #1 OR #2 OR #3**

#5 'virus'/exp

#6 'virus infection'/exp

#7 'virus examination'/exp

#8 'viral phenomena and functions'/exp

#9 'virus load'/exp

#10 virus*:ti,ab,kw OR viral*:ti,ab,kw OR virol*:ti,ab,kw

#11 antiviral*:ti,ab,kw OR anti-infective*:ti,ab,kw

#12 retrovir*:ti,ab,kw OR leukovir*:ti,ab,kw OR oncovir*:ti,ab,kw OR deltaretrovir*:ti,ab,kw OR lentivir*:ti,ab,kw OR HIV:ti,ab,kw OR HIVs:ti,ab,kw OR ‘acquired immunodeficiency syndrome’:ti,ab,kw OR AIDS:ti,ab,kw OR HTLV:ti,ab,kw OR HMTV:ti,ab,kw OR H-MTV:ti,ab,kw OR herpesvir*:ti,ab,kw OR alphaherpesvir*:ti,ab,kw OR simplexvir*:ti,ab,kw OR varicellovir*:ti,ab,kw OR betaherpesvir*:ti,ab,kw OR roseolovir*:ti,ab,kw OR rhadinovir*:ti,ab,kw OR gammaherpesvir*:ti,ab,kw OR lymphocryptovir*:ti,ab,kw OR cytomegalovir*:ti,ab,kw OR CMV:ti,ab,kw OR HCMV:ti,ab,kw OR ‘inclusion disease*’:ti,ab,kw OR herpes:ti,ab,kw OR herpet*:ti,ab,kw OR ‘HHV-5’:ti,ab,kw OR HHV5:ti,ab,kw OR ‘HHV-1’:ti,ab,kw OR HHV1:ti,ab,kw OR ‘HSV-1’:ti,ab,kw OR HSV1:ti,ab,kw OR ‘HHV-2’:ti,ab,kw OR HHV2:ti,ab,kw OR ‘HSV-2’:ti,ab,kw OR HSV2:ti,ab,kw OR ‘HHV-3’:ti,ab,kw OR HHV3:ti,ab,kw OR ‘HSV-3’:ti,ab,kw OR HSV3:ti,ab,kw OR varicella*:ti,ab,kw OR zoster:ti,ab,kw OR shingles:ti,ab,kw OR VZV:ti,ab,kw OR chickenpox:ti,ab,kw OR ‘HHV-6*’:ti,ab,kw OR HHV6*:ti,ab,kw OR HBLV:ti,ab,kw OR roseola:ti,ab,kw OR ‘exanthema subitum’:ti,ab,kw OR ‘HHV-7’:ti,ab,kw OR HHV7:ti,ab,kw OR ‘HHV-8’:ti,ab,kw OR HHV8:ti,ab,kw OR KSHV:ti,ab,kw OR ‘Kaposi*’:ti,ab,kw OR ‘HHV-4’:ti,ab,kw OR HHV4:ti,ab,kw OR ‘Epstein-Barr’:ti,ab,kw OR EBV:ti,ab,kw OR burkitt*:ti,ab,kw OR mononucleo*:ti,ab,kw OR leukopla*:ti,ab,kw OR coronavir*:ti,ab,kw OR alphacoronavir*:ti,ab,kw OR betacoronavir*:ti,ab,kw OR SARS:ti,ab,kw OR MERS:ti,ab,kw OR SARS2:ti,ab,kw OR ‘SARS-COV-2’:ti,ab,kw OR COVID*:ti,ab,kw OR 2019nCoV:ti,ab,kw OR ‘2019-nCoV’:ti,ab,kw OR ‘HCoV-19’:ti,ab,kw OR ‘severe acute respiratory syndrome’:ti,ab,kw OR ‘Middle East respiratory syndrome’:ti,ab,kw

#13 flavivir*:ti,ab,kw OR arbovir*:ti,ab,kw OR ‘yellow fever’:ti,ab,kw OR YFV:ti,ab,kw OR dengue:ti,ab,kw OR DENV:ti,ab,kw OR ‘breakbone fever’:ti,ab,kw OR ‘West Nile’:ti,ab,kw OR WNV:ti,ab,kw OR ‘tick borne’:ti,ab,kw OR ‘Japanese encephalitis’:ti,ab,kw OR ‘Saint Louis’:ti,ab,kw OR ‘St Louis’:ti,ab,kw OR zika:ti,ab,kw OR zikV:ti,ab,kw OR hepacivir*:ti,ab,kw OR ‘hepatitis C’:ti,ab,kw OR hepadnavir*:ti,ab,kw OR orthohepadnavir*:ti,ab,kw OR HBV:ti,ab,kw OR ‘hepatitis B’:ti,ab,kw OR picornavir*:ti,ab,kw OR hepatovir*:ti,ab,kw OR ‘hepatitis A’:ti,ab,kw OR HAV:ti,ab,kw OR enterovir*:ti,ab,kw OR echovir*:ti,ab,kw OR coxsackie*:ti,ab,kw OR CVB3:ti,ab,kw OR cardiovir*:ti,ab,kw OR herpangin*:ti,ab,kw OR hepevir*:ti,ab,kw OR HEV:ti,ab,kw OR ‘hepatitis E’:ti,ab,kw OR papillomavir*:ti,ab,kw OR alphapapillomavir*:ti,ab,kw OR betapapillomavir*:ti,ab,kw OR gammapapillomavir*:ti,ab,kw OR mupapillomavir*:ti,ab,kw OR HPV:ti,ab,kw OR wart:ti,ab,kw OR filovir*:ti,ab,kw OR ebola*:ti,ab,kw OR EBOV:ti,ab,kw OR marburg*:ti,ab,kw OR MARV:ti,ab,kw OR togavir*:ti,ab,kw OR alphavir*:ti,ab,kw OR chikungunya:ti,ab,kw OR CHIKV:ti,ab,kw OR ‘Onyong-nyong’:ti,ab,kw OR ‘nyong nyong’:ti,ab,kw OR ‘barmah forest’:ti,ab,kw OR ‘ross river’:ti,ab,kw OR Sindbis:ti,ab,kw OR hantavir* OR orthohantavir*:ti,ab,kw OR hantaan*:ti,ab,kw OR ADNV:ti,ab,kw OR puumala:ti,ab,kw OR SEOV:ti,ab,kw OR ‘sin nombre’:ti,ab,kw OR SNV:ti,ab,kw OR anellovir*:ti,ab,kw OR alphatorquevir*:ti,ab,kw OR ‘torque teno’:ti,ab,kw OR TTV:ti,ab,kw OR SENV:ti,ab,kw OR SEN-V:ti,ab,kw OR matonavir*:ti,ab,kw OR rubivir*:ti,ab,kw OR rubella:ti,ab,kw OR ‘German measles’:ti,ab,kw OR RuV:ti,ab,kw

**#14 #5 OR #6 OR #7 OR #8 OR #9 OR #10 OR #11 OR #12 OR #13**

#15 'pasteurization'/exp

#16 pasteuri*:ti,ab,kw OR holder:ti,ab,kw OR HoP:ti,ab,kw OR ‘low temperature long time’:ti,ab,kw OR LTLT:ti,ab,kw OR flash:ti,ab,kw OR ‘high temperature short time’:ti,ab,kw OR HTST:ti,ab,kw OR thermal:ti,ab,kw

#17 'high temperature'/exp

#18 heat:ti,ab,kw OR heating:ti,ab,kw OR nonthermal:ti,ab,kw

#19 'hydrostatic pressure'/exp

#20 ‘high hydrostatic pressure’:ti,ab,kw OR HHP:ti,ab,kw OR ‘high-pressure processing’:ti,ab,kw OR HPP:ti,ab,kw

#21 'ultraviolet radiation'/exp

#22 ultraviolet:ti,ab,kw OR UVC:ti,ab,kw OR UV-C:ti,ab,kw

#23 'gamma radiation'/exp

#24 ‘gamma-irradiat*’:ti,ab,kw OR ‘gamma-radiat*’:ti,ab,kw

#25 'ultrasound'/exp

#26 sonicat*:ti,ab,kw OR sonificat*:ti,ab,kw OR ultrasound:ti,ab,kw OR ultrasonic:ti,ab,kw OR thermoultrasound:ti,ab,kw OR processing:ti,ab,kw OR irradiation*:ti,ab,kw OR inactivation*:ti,ab,kw

#27 'freeze drying'/exp

#28 'freezing'/exp

#29 'cold'/exp

#30 'refrigeration'/exp

#31 'freeze thawing'/exp

#32 'thawing'/exp

#33 freez*:ti,ab,kw OR frozen:ti,ab,kw OR refrigerat*:ti,ab,kw OR thaw*:ti,ab,kw

#34 'food preservation'/exp

#35 'food storage'/exp

#36 storag*:ti,ab,kw

**#37 #15 OR #16 OR #17 OR #18 OR #19 OR #20 OR #21 OR #22 OR #23 OR #24 OR #25 OR #26 OR #27 OR #28 OR #29 OR #30 OR #31 OR #32 OR #33 OR #34 OR #35 OR #36**

#38 #4 AND #14 AND #37

#39 #38 AND [embase]/lim
